# Health literacy in the context of child health promotion: a scoping review of conceptualizations and descriptions

**DOI:** 10.1186/s12889-024-17955-7

**Published:** 2024-03-14

**Authors:** Wieke Van Boxtel, Katarina Jerković-Ćosić, Linda J. Schoonmade, Mai J. M. Chinapaw

**Affiliations:** 1 Research group Innovation in Preventive Healthcare, HU University of Applied Sciences Utrecht, Heidelberglaan 7, Utrecht, 3584 CS The Netherlands; 2 Medical Library, Vrije Universiteit Amsterdam, De Boelelaan 1117, Amsterdam, 1007 MB The Netherlands; 3 Amsterdam UMC location Vrije Universiteit Amsterdam, Public and Occupational Health, De Boelelaan 1117, Amsterdam, The Netherlands; 4 Health Behaviour and Chronic Diseases, Methodology, Amsterdam Public Health, Amsterdam, The Netherlands

**Keywords:** Health literacy, Children, Health Promotion, Learning outcomes, School health, Scoping review

## Abstract

**Background:**

Increasing health literacy (HL) in children could be an opportunity for a more health literate future generation. The aim of this scoping review is to provide an overview of how HL is conceptualized and described in the context of health promotion in 9–12-year-old children.

**Methods:**

A systematic and comprehensive search for ‘health literacy’ and ‘children’ and ‘measure’ was performed in accordance with PRISMA ScR in PubMed, Embase.com and via Ebsco in CINAHL, APA PsycInfo and ERIC. Two reviewers independently screened titles and abstracts and evaluated full-text publications regarding eligibility. Data was extracted systematically, and the extracted descriptions of HL were analyzed qualitatively using deductive analysis based on previously published HL definitions.

**Results:**

The search provided 5,401 original titles, of which 26 eligible publications were included. We found a wide variation of descriptions of learning outcomes as well as competencies for HL. Most HL descriptions could be linked to commonly used definitions of HL in the literature, and some combined several HL dimensions. The descriptions varied between HL dimensions and were not always relevant to health promotion. The educational setting plays a prominent role in HL regarding health promotion.

**Conclusion:**

The description of HL is truly diverse and complex encompassing a wide range of topics. We recommend adopting a comprehensive and integrated approach to describe HL dimensions, particularly in the context of health promotion for children. By considering the diverse dimensions of HL and its integration within educational programs, children can learn HL skills and competencies from an early age.

**Supplementary Information:**

The online version contains supplementary material available at 10.1186/s12889-024-17955-7.

## Background

Health literacy (HL) can be defined as “the knowledge, motivation and competences to access, understand, appraise and apply health information to make informed decisions about health” [1]. In children, explicit attention is needed to stimulate HL and thereby learn healthy behaviors [2, 3]. The focus should be on increasing the individuals’ potential for health opportunities [4], as inadequate HL sustains or increases socio-economic health inequity [5, 6].

Most school-aged children encounter the context of health promotion and disease prevention through primary prevention. HL is relevant on many occasions and contexts of children’s daily life that have a potential impact on the health and well-being beyond the clinical setting [7]. However, a smaller proportion of children experience health care utilization or disease management activities due to illness or a chronic condition, requiring specific HL needs. This paper focuses on HL for the general population of children aged 9–12 years within health promotion contexts, for which explicit attention is needed to stimulate HL and thereby promote healthy behaviors.

Adequate HL skills are needed to engage in health promotion and prevention. Children are developing cognitively, physically, as well as gradually gaining responsibility in health-related decisions, specifically, children aged 9–12 years old develop independence, while they transition from playful learning towards emphasizing academics in school and gaining information from peers and media [8, 9]. Using this development stage for advancing HL might help this population become health-literate adults.

HL is a complex construct, with multiple determinants interacting in different contexts *(*e.g., health promotion or health care) and settings (e.g., schools or hospitals). Multiple variants or types of HL have emerged around health themes such as Nutritional/Food Literacy [10], Physical (Health) Literacy [11] and, Mental Health Literacy [12]. Additionally, some HL themes and measures are related to a specific disease or to information sources, for example: Diabetes Literacy [13] and Media Literacy [14].

Furthermore, various definitions of HL are used worldwide. Bröder and colleagues [7] found 12 definitions and 21 models in their systematic review on HL in children and adolescents up to 18 years old. In 2008, Nutbeam concluded that HL is a multi-dimensional construct of functional, cognitive/interactional, and critical literacy skills [15]. In 2012, Sørensen et al. [1] described the core of their HL model as “the competencies related to the process of accessing, understanding, appraising and applying health-related information” (p.8). In the same year Paakkari and Paakkari [16] considered HL as a learning outcome of education. They described [16] that “HL comprises a broad range of knowledge and competencies that people seek to encompass, evaluate, construct and use…” (p5).

In the literature as well as in practice, HL is conceptualized in multiple ways due to the complexity of the construct, variety of the contexts and settings in which it is used, and its various definitions worldwide. The latest reviews indicate a need for defining the construct and its dimensions to be tailored to the specific target population, setting and context [7, 17, 18]. In this review we refer to HL in various conceptualizations with multiple dimensions.

The aim of this scoping review is to provide an overview of how HL is conceptualized and described in the context of health promotion in children aged 9–12 years. This may gain valuable insights for health professionals, (health) education professionals and researchers, into how HL is used in this age group and context, ultimately informing future attempts to promote HL in children. By means of a scoping review the following research question will be answered: How is HL described for children aged 9–12 years in the context of health promotion? This scoping review summarizes the findings from a heterogeneous body of knowledge regarding the methods and disciplines in which HL is described for children [19] specifically in the context of health promotion, which adds to the existing reviews based on scientific literature only.

## Methods

### Search methods

This scoping review was planned, conducted, and reported in accordance with PRISMA ScR [20] and Standards for Reporting Qualitative Research (SRQR) [21] to limit the risk of bias within this research design. The study was registered in the Open Science Framework (10.17605/OSF.IO/R469X).

A comprehensive search was performed in the bibliographic databases PubMed, Embase.com, CINAHL (via Ebsco), APA PsycInfo (via Ebsco) and ERIC (via Ebsco) from inception to July 2020 and after 2 years a search update was performed till August 8th 2022, in collaboration with medical librarians (MM and LS, respectively). Search terms included controlled terms (MeSH in PubMed and Emtree in Embase, thesaurus terms in PsycInfo and ERIC, and CINAHL Subject Headings) as well as free text terms. The following terms were used (including synonyms and closely related words) as index terms or free text words: ‘health literacy’ and ‘children’ and ‘measure’. The search was performed without date or language restrictions. Duplicate publications were excluded. The full search strategies can be found in Supplementary material 4.

### Selection process

Two reviewers (WvB and KC) independently screened all potentially relevant titles and abstracts for eligibility using Rayyan [22]. Eligibility criteria were:

1. Construct:
  - indication of descriptions of HL dimensions, and/or;
  - functional, cognitive, interactive, and critical HL skills, and/or;
  - related variants described in conjunction with HL such as physical (health) literacy or food/nutritional literacy.
2. Context:
  - the context of health, and/or;
  - health promotion, and/or;
  - disease prevention (without risk factors).
3. Population: children aged 9–12 years and;
4. Document Criteria:
  - peer reviewed research papers;
  - government reports, or;
  - educational standards, and;
  - all written in English.

For the updated search performed in August 2022, new titles and abstracts were screened in ASReview Lab v1.0 (2022) using the default settings by one reviewer (WvB). ASReview prioritizes the found publications on eligibility using active learning, based on prior knowledge on inclusion and exclusion decisions from the original search which the reviewer indicated. New publications were selected by the reviewer in ASReview following the stopping rule for screening [23]. In Supplementary material 4 the complete process of selection in ASReview is described.

Two reviewers (WvB and IS) independently evaluated the full text for the eligibility criteria using Covidence Systematic Review Software (2021). Differences in judgement were resolved through a consensus procedure. The full text review for the updated search was evaluated by WvB.

### Data extraction

One reviewer (WvB) performed data extraction in Covidence with data extraction 2.0 (2022). The data was extracted in three groups. The first group contained general characteristics of the included studies: (1) year of publication; (2) country; (3) aim of study; (4) study design; (5) study methods/data collection; (6) data preparation/analysis; (7) population description; (8) recruitment of participants; and (9) total number of participants. Secondly, data on the HL construct for the target population was extracted: (10) construct HL; (11) levels in HL (based on Nutbeam, 2008); (21) context; (22) setting; (23) topics of descriptions; and (24) description of HL dimensions. Lastly, for included studies on measuring HL, data was extracted for (12) measurement instrument name; (13) mode of administration; (14) target population; (15) population age; (16) N items; (17) response options; (18) range of scoring; (19) language; and (20) validity/reliability. The data extraction template can be found in Appendix B.

### Data analysis

The extracted data of the descriptions of HL dimensions was qualitatively analyzed (by WvB) using ATLAS.ti (version 22) following three steps. First, suitability for deductive coding based on commonly used definitions by Sorenson et al. [1], Paakkari and Paakkari [16], and Nutbeam et al. [15] was checked. Frequently used words in the descriptions were searched through word lists to identify a match with descriptions of definitions. After confirming suitability, segments were read and deductively coded with the most applicable code from the definitions and HL skills (Table 1). Three automated analyses were performed on coded data: concept analysis, occurrence of codes/code groups, and segment analysis for code co-occurrence. Concept analysis was utilized to identify topics used in the descriptions of HL dimensions based on noun phrases. The occurrence of codes or code groups was analyzed to explore distribution of definitions and skills in documents, per setting and for measurement instruments. Finally, we analyzed segments which included one or more codes from different HL definitions using the operators ‘And’, ‘Within’, ‘Encloses’, ‘Overlaps’ and ‘Overlapped by’ to explore code co-occurrence. Segments which could not be deductively coded, and revealed relevant new information, were open-coded based on the subject of the segment. Rich examples were identified to illustrate the description of HL and deductive coding was used to ground the descriptions in the theory of HL definitions.

**Table 1:** Code groups and codes used for deductive analysis

Code Groups based on HL definitions		Codes
**HL Competencies based** on Sørensen et al. (2012)		• Accessing • Understanding • Appraising • Applying
**HL Learning outcome** based on Paakkari & Paakkari (2012)		• Theoretical knowledge • Practical knowledge • Critical thinking • Self-awareness • Citizenship
**HL Skills** based on Nutbeam (2000)	**Functional skills**	• Reading • Pronunciation • Writing • Numeracy
	**Cognitive skills**	• Knowledge • Comprehension • Extracting information • Derive meaning
	**Interactive skills**	• Communication
	**Critical skills**	• Analyzing information (critically) • Decision making • Use/apply information

## Results

**Fig. 1:**
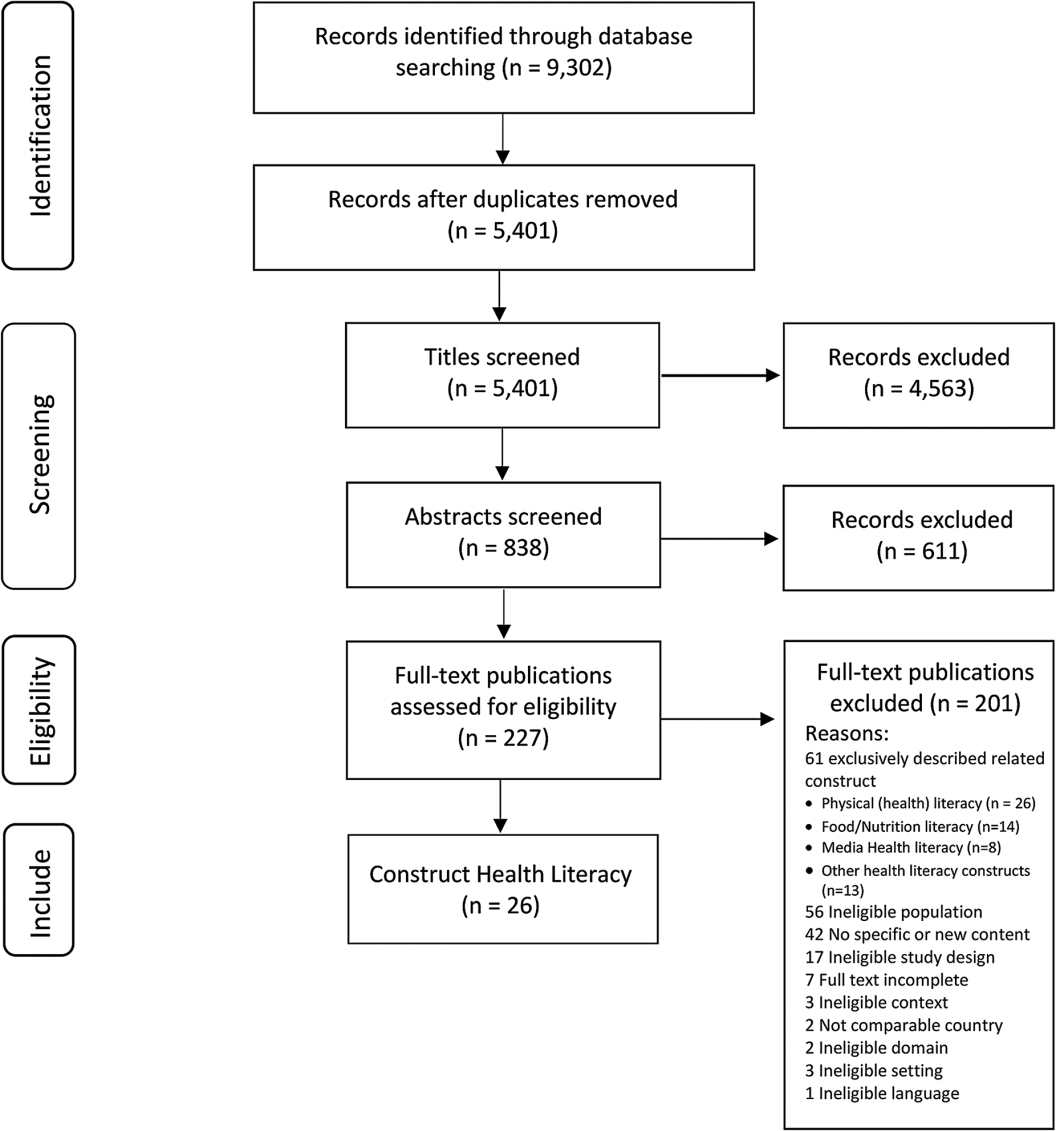
Flowchart of the search and selection procedure of publications

Figure 1 presents the flow chart of the search and selection process. The literature search generated a total of 9,302 references out of which 26 publications were eligible for inclusion. In spite of the search including specific contexts, we had to exclude some publications based on domains and settings which did not match the health promotion context. The focus of this scoping review is on how dimensions of general HL within health promotion in children are described. A review of the related HL constructs focused on specific areas will be described elsewhere.

### Study characteristics

Table 2 presents the characteristics of the included publications. Publication dates ranged from 2000 to 2022, with the majority (*n* = 17) published after 2012. Five publications were grey literature such as government reports (*n* = 2) and educational program designs (*n* = 3). Other scientific publications reported on randomized controlled trials (*n* = 2), literature reviews (*n* = 2), development (*n* = 3) and validations studies (*n* = 2) for measurement instruments, cross sectional studies (*n* = 6), educational program (*n* = 3) evaluation studies (*n* = 3) and qualitative studies (*n* = 3). The age range of participants in the sampled studies was 5–15 years old.

**Table 2:** Study characteristics of included publications in alphabetic order, overview of study designs, country, sample, setting, and developed measurement instruments

First Author	Title	Study design/publication type	Country	Sample^a^	Setting	Developed Measurement instrument
Arvanitis et al. (2020) [24]	Topical review: Proposing a developmentally informed research agenda for the study of health literacy in children	Literature Review	United States	Children (until adolescence adulthood) (age of 10 based on [25]	Daily life/Public health	NA
Bhagat et al. (2018) [26]	The Relationship Between Health Literacy and Health Conceptualizations: An Exploratory Study of Elementary School-Aged Children	Qualitative study	United States	8–11 years, *N* = 29	After school	Yes, NVS adaptation by researchers
Boberova et al. (2017) [27]	Democratic school health education in a post-communist country	Cluster-Randomized-Controlled Trail	Slovakia	9–11 years, *N* = 180	School	NA
Bollweg et al. (2019) [28]	Measuring children’s health literacy: Current approaches and challenges	Systematic review (book chapter)	Germany	6–12 years	Multiple settings	NA
Bollweg et al. (2020) [29]	Adapting the European Health Literacy Survey for Fourth-Grade Students in Germany: Questionnaire Development and Qualitative Pretest	Measurement instrument Development study	Germany	9–11 years, grade 4, *N* = 30	School	Yes, HLS-EU-Q adapted for children (later adapted to HLS-Child-Q15-DE)
Bollweg et al. (2020) [30]	Adapting the European Health Literacy Survey Questionnaire for Fourth-Grade Students in Germany: Validation and Psychometric Analysis	Validation study	Germany	8–12 years, grade 4, *N* = 907	School	Yes, HLS-Child-Q15-DE
Brey et al. (2007) [31]	Enhancing Health Literacy through Accessing Health Information, Products, and Services: An Exercise for Children and Adolescents	Educational program design	United States	grades 6–12, [11–18 years]	School	NA
Brown et al. (2007) [32]	Early adolescents perceptions of health and health literacy	Cross sectional study	United States	9–13 years, grade 4, *N* = 1178	Health education center	NA
California State Dept. (2003) [33]	Health Framework for California Public Schools Kindergarten through Grade Twelve	Government report	United States	Schoolchildren from elementary to secondary school [5–10 and 11–14 years]	School	NA
De Buhr et al. (2020) [34]	Potentials of School Nursing for Strengthening the Health Literacy of Children, Parents and Teachers	Evaluation study	Germany	11 + years old elementary and secondary schools, *N* = 2530	School	Yes, HLSAC translated in German
Diamond et al. (2011) [35]	The development of building wellness™, a youth health literacy program	Evaluation study	United States	7–10 years, *N* = 232	After school	Yes, REALM-Teen
Driessnack et al. (2014) [36]	Using the “Newest Vital Sign” to assess health literacy in children	Validation study	United States	7–12 years, *N* = 94 (Parent-Child dyads *N* = 47)	Public	Yes, NVS
Franze et al. (2011) [37]	Implementation and evaluation of the population-based programme “health literacy in school-aged children” (GeKoKidS)	Randomized controlled trial	Germany	9–13 years, *N* = 882	School	Yes, GeKoKidS self developed items
Fretian et al. (2020) [38]	Exploring Associated Factors of Subjective Health Literacy in School-Aged Children	Cross sectional study	Germany	9–10 years, *N* = 899	School	Yes, HLS-Child-Q15-DE
Guo et al. (2020) [39]	Adolescent Health Literacy in Beijing and Melbourne: A Cross-Cultural Comparison	Cross sectional study	Australia-China	11–13 years, grades 7–9, *N* = 770	School	NA
Hahnraths et al. (2021) [40]	Measuring and Exploring Children’s Health Literacy in The Netherlands: Translation and Adaptation of the HLS-Child-Q15	Measurement instrument Development study	Netherlands	8–11 years, grades 3–4, *N* = 215	School	Yes, HLS-Child-Q15-NL
Haynes (2004) [41]	Health information @Preuss (HIP): integrating online health information into the curricula of a middle school	Educational program design	United States	11–15 years, grades 6–9, *N* = 250	School	NA
Howe et al. (2018) [42]	Poor Performance of Children Age 7 to 13 Years on the Newest Vital Sign	Cross sectional study	United States	7–13 years and parents, *N* = 251	Public	NA
Knisel et al. (2020) [43]	Promotion of Elementary School Students’ Health Literacy	Evaluation study	Germany	6–12 years, grades 2–4, *N* = 137	School	NA
Kostenius et al. (2017) [44]	Health literacy in an age of technology–schoolchildren’s experiences and ideas	Qualitative study	Sweden	10–11 years, grade 4 *N* = 540	School	NA
Liao et al. (2017) [45]	Defining Taiwanese children’s health literacy abilities from a health promotion perspective	Qualitative study	Taiwan	11–12 years, grade 6, *N* = 53	Daily life/Public health	NA
Liu et al. (2018) [46]	Development and validation of the Taiwan Children’s Health Literacy Test	Measurement instrument Development study	Taiwan	11–12 years, grade 6, *N* = 585	School	Yes, TCHL
Paakkari et al. (2019) [47]	Health literacy and the school curriculum: The example of Finland	Educational program design	Finland	5–15 years	School	NA
Schmidt et al. (2010) [48]	Health-related behavior, knowledge, attitudes, communication and social status in school children in Eastern Germany	Cross sectional study	Germany	9–13 years (Mean 10.4), grade 5, *N* = 852	School	NA
South Dakota State Dept. (2000) [49]	South Dakota Health Education Standards: A Resource Guide for Achieving Health Literacy	Government report	United States	Grade 3–5 [8–11 years] and Grade 6–9	School	NA
Yu et al. (2012) [50]	Study on student health literacy gained through health education in elementary and middle schools in China	Cross sectional study	China	Elementary school grade 3 and 4 [8–10 years], *N* = 8008	School	NA

^a^ School/grade level or population was translated in to the supposed age range based on country and educational system, noted as [. years]

GeKoKids: GesundheitsKompetenzKids, HLS-EU-Q: Health Literacy Survey Europe Questionnaire, HLS-Child-Q15-DE/NL: Health Literacy Survey-Child_Q15 German/Dutch, HLSAC: Health Literacy for School-Aged Children NVS: Newest Vital Sign, REALM-Teen: Rapid Estimate for Adolescent Literacy in Medicine-Teen, TCHL: Taiwanese Children’s Health Literacy

To analyze the HL descriptions, publications were grouped based on setting: School, After school and Public setting.

In nine publications, a measurement instrument was developed and/or evaluated. Most instruments were adapted from existing instruments for adults. However, one instrument was newly developed for children by Franze et al. [37].

### The use of definitions and skills

**Fig. 2:**
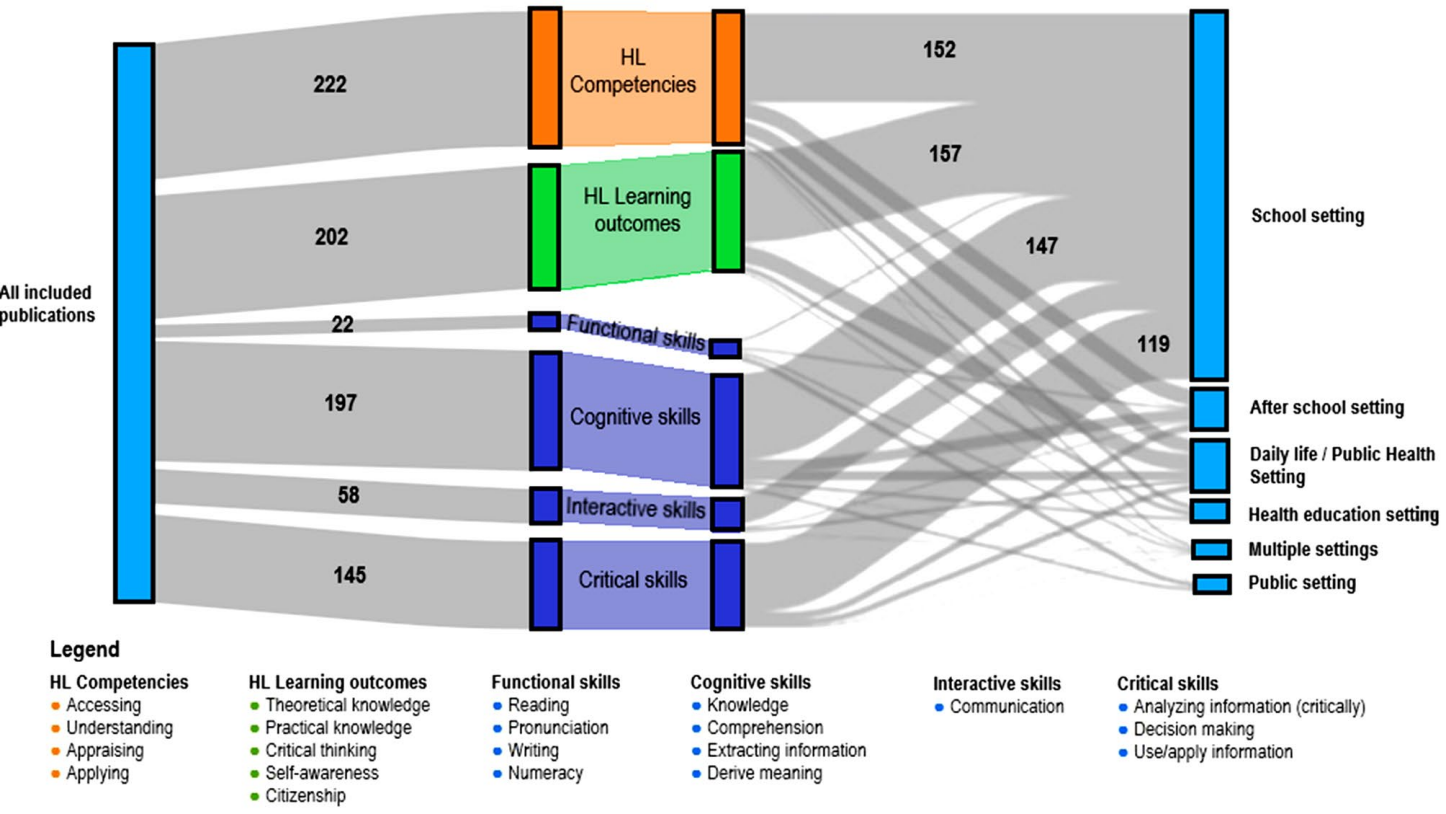
Sankey diagram presenting the distribution of code groups with imbedded codes (in legenda) based on competencies definition (orange), learning outcome definition (green), HL skills (blue) for all publications towards the left, and per setting towards the right. The numbers in the figure indicate the total number of quotations coded in the code group

Deductive analysis showed that the descriptions of HL were conceptualized as competencies, learning outcomes and skills. Figure 2 presents the distribution of all code groups, as well as how often codes from that group were grounded within the included literature. The competencies definition, based on Sørensen et al. [1] was dominant, occurring in a total of 222 quotations across all publications. Additionally, many descriptions were related to the learning outcomes definition from Paakkari et al. [16]. All included publications indicated HL skills, as described by Nutbeam et al. [15]. With ‘knowledge’ and ‘comprehension’ for cognitive skills and ‘critically analyzing information’ and ‘use/apply information’ for critical skills being dominant. In some educational oriented publications, descriptions from both HL definitions [1, 16] were found [24, 31–34, 44–46, 49, 50]. The cognitive skill ‘knowledge’ was frequently described, closely followed by the competency ‘understanding’. This was reflected by the occurrence of descriptions on the skills ‘comprehension’ and ‘derive meaning’, as well as ‘critical thinking’ for learning outcomes.

Nine codes emerged through open coding. Kostenius and colleagues [44] described ‘caring and confirming’ and ‘engaging and empowering’ as important. Three new codes were found in the South Dakota Education Standards [49]: ‘problem solving’, ‘goal setting’, and ‘stress management’. ‘Motivation’ [28, 38], ‘recognition’, ‘help-seeking behavior’, and ‘satisfaction’ [28] could also not be related to the predefined definitions or skills.

### HL definitions and skills in measurement instruments

We found nine studies which developed or validated an HL measurement instrument for children. Five instruments were developed or validated after the latest reviews on instruments from 2018 [17, 51] namely: Taiwanese Children’s Health Literacy (TCHL) [46], Newest Vital Sign (NVS) adaptation [26], Health literacy Survey-Child Questionnaire 15 in German and Dutch (HLS-Child_Q15-DE and HLS-Child-Q15-NL) [29, 30, 38, 40], and Health Literacy for School-Aged Children (HLSAC) in German [34]. We also found the GeKoKidS (GesundheitsKomptenzKids) instrument [37] which was included in the review by Okan et al. [51] in the study by Schmidt [48], however we found GeKoKids through the study by Franze et al. [37]. Most instruments found their origin in validated instruments for older populations, contexts, and settings outside health promotion context. The competencies definition was used most often, indicating an emphasis on understanding [26, 29, 30, 35, 37, 38, 40, 46]. Two instruments, namely TCHL and HLSAC, could be linked to HL as learning outcomes [34, 46]. In the study by Diamond et al., [35] the preferences of teens could be linked to the learning outcome definition but did not match align with the original instrument for that definition. In all instruments we found descriptions of ‘cognitive’ and ‘critical skills’ most frequently.

### Settings in which HL is described

The school setting was the most prevalent setting with an almost equal distribution of descriptions related to the learning outcome definition as to the competency definition (Fig. 2). Within the school setting, HL skills were mostly focused on learning ‘cognitive’ and ‘“critical skills’ while ‘functional skills’ were rarely mentioned in all settings. The descriptions of learning outcomes varied from activities for learning or play to actual learning outcomes for educational programs. Far less descriptions were described in the afterschool, daily life, and public health settings.

### Terms to describe HL

The top five terms found in all publications were *Health*, *Information*, *Can*, *Understand* and *Ability*. The diversity of terms used to describe HL dimensions confirmed the need for a concept analysis on the descriptions of HL in the included literature to gain insight into how topics were described in more detail.

### Frequently used topics

Health was the most frequently occurring topic in all descriptions, as “health information”, “health issues”, “your health” and “personal health”. Information was second, however, in the codes ‘self-awareness’, ‘citizenship’, ‘functional skills’ and ‘interactive skills’ information was not in the top five topics. Information was described as “health information” and “good/valid information”.

For the competencies definition, most frequently found topics were Service (26), Products (22), Development (17), Food (17) and Skill (15). The learning outcomes had more diverse topics: Community (30), Services (18), Family (17), Products (17), Disease (13) and Development (13). Functional skills, such as ‘reading’ and ‘numeracy’, were only assessed using the NVS tool, in which children read an ice-cream label [36, 42]. ‘Cognitive’” and ‘critical skills’” had the most diverse topics which were well-grounded in the literature. The top 5 topics for ‘cognitive’, ‘interactive’, and ‘critical skills’ were Development (20), Life (20), Service (20), Product (18) and Community (18). All topics per code with corresponding noun phrases are presented in Appendix G.

### Co-occurence of HL dimensions within the descriptions of HL

**Fig. 3:**
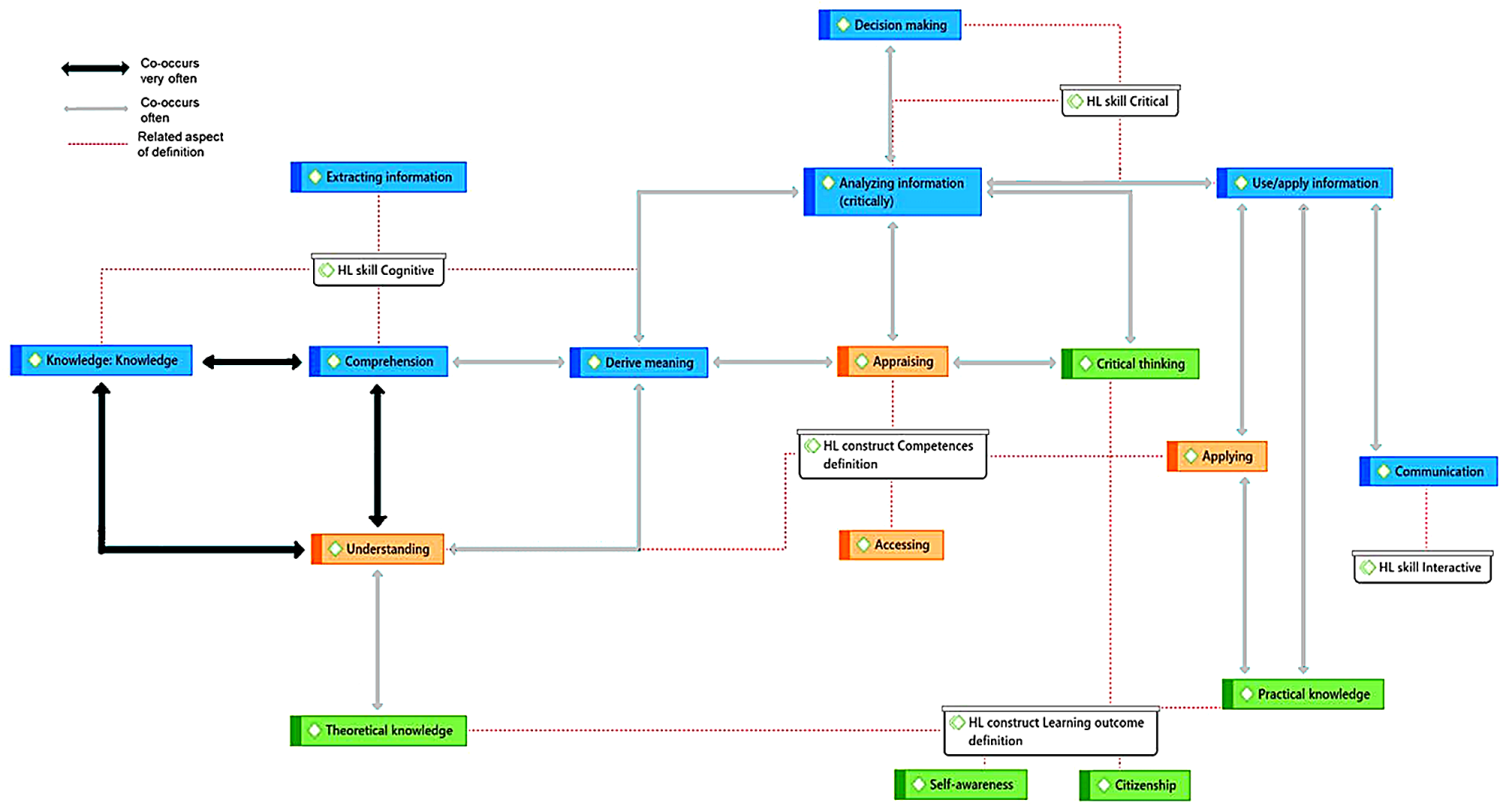
Network of co-occurrences between HL dimensions found in the descriptions of HL. The arrows vary in thickness to indicate whether the co-occurrence was found often (over 20 times) or very often (over 35 times)

The complexity of the construct was reflected in the co-occurrence of codes within the descriptions of HL with links to multiple HL dimensions. Figure 3 represents the co-occurance of codes, and the thicker arrow lines in this figure represent more co-occurences. A description with quotations is provided for the most common co-occurences.

### Understanding, theoretical knowledge, knowledge, comprehension and derive meaning

The co-occurrences between ‘understanding’ and ‘knowledge’ described what children need to understand or referred to the specific knowledge children need to elaborate on in order to evaluate their understanding of the specific knowledge. For example, in Liao et al. [45] describe the need for “[b]*asic health knowledge and skills that can be used to take health-related actions.”* (p. 74) in 11 reference abilities (p.77).

‘Understanding’ and ‘derive meaning’ referred to the processing of information. In Bhagat et al. [26], they ask about the difficulty of a HL related task: “*Is it easy or hard to understand the health information you get from (fill in each source)? What makes is hard or easy to understand?”* (p.3).

‘Understanding’ and ‘theoretical knowledge’ was found as a description of an objective instruction with assessment criteria by Paakkari & Paakkari [47]. “*Pupils should be able to describe life course stages and to explain key characteristics of growth and development in puberty, plus individual variations”* (p. 528).

‘Knowledge’ and ‘comprehension’ often co-occurred where ‘knowledge’ indicated what children know about health topics whereas ‘comprehension’ indicated an understanding of what children can do in certain situations. An example was found in scenario eight by Liu et al. [46] *“Can wearing a mask prevent you from getting sick? What is your understanding of sanitary masks? Learning outcome: Can understand the influence of lifestyle on disease”* (p.39).

The co-occurrence of ‘comprehension’ and ‘derive meaning’ was found in Boberova [27] describing the following criteria students should consider: “*How is your notion of health different from your schoolmates’ view? What is and makes it different (similar) [and why?]”* (p.475).

### Appraising, critical thinking, derive meaning and analyzing information critically

Examples of ‘appraising’ and ‘critical thinking’ were found in De Buhr et al., [34]. For instance, the following items from the instrument De Buhr et al. used for children illustrate how critical thinking is necessary to judge and/or compare health information: “*Ability to compare health-related Information from different sources; Ability to decide if health-related information is right or wrong.”* (p.6).

‘Appraising’ and ‘derive meaning’ were found in quotations from Bollweg [29]. They asked children to indicate how difficult it is for them to: “*Judge what helps a lot for you to stay healthy and what does not help much?”* (p. e127).

An example of ‘critical thinking’ and ‘analyzing information critically’ were found in Brey [31], in the following learning outcome: “*Identify at least 3 reasons a health agency, organization, or institution would be considered a credible source of health information, services, or products.”* (p.641).

‘Derive meaning’ and ‘analyzing information critically’ was found as a well-grounded co-occurrence with an example in Franze [37], describing what we could ask children about prevention on starting smoking. Two examples from the measure are: “*How do children learn about the health-related consequences of tobacco-smoking?” and “How do they evaluate these consequences?”* (p.341).

### Applying, practical knowledge, communication, analyzing information critically, Use/apply information and decision making

The co-occurrence of ‘applying information’ and ‘practical knowledge’, when children have been ‘analyzing information critically’ and ‘deciding’ it is appropriate to use was found in learning outcomes in the *Health Framework for California Public Schools* [33]; “*Students will understand and demonstrate behaviors that prevent disease and speed recovery from illness.”* (p.68) and the *South Dakota Health Education Standard* [49]; “*Explain ways to achieve and maintain good health; determine personal health progress and make adjustments for improvement.”* (p.36).

The relationship between ‘communication’ and ‘use/apply information’ was found in assessment criteria by Paakkari & Paakkari [47] for grade 4–6: *“Pupils should be able to describe practices related to…practices for expressing and regulating emotions, and for applying them in different roles”* (p.528). In Liu et al. [46] the authors described short scenarios with a follow-up question in which students can demonstrate their skills: “*[students] can understand and respond to other people in interpersonal interactions and can express proper rejection skills in health-related life situations”* (p.38).

## Discussion

This scoping review provides insights into the description of the HL construct within the context of health promotion.

In recent years, systematic reviews by Bröder et al. [7], Okan et al. [51] and Guo et al. [17] have reported a variety of definitions and conceptualizations used to describe and assess HL in children. In addition, the current review provides a more specific insight into which topics and concepts of health, are used in the description and assessment of HL dimensions for children aged 9–12 years, specifically within the health promotion context. Moreover, our qualitative analysis revealed various co-occurrences of HL dimensions in the descriptions of related to different definitions.

### Interpretation of results

Similar to previous reviews [7, 17, 51], we found that how HL is conceptualized and described for children is still remarkably diverse. We believe this is due to the use of multiple definitions in research, which differ in dimensions and description. Moreover, we found many topics within the descriptions of HL skills and competencies, which suggest an even larger diversity. We also see this in adult HL where a recent review by Malloy-Weir et al. [52] revealed 250 definitions with differences in actions and skills. Although the definitions by Sørensen et al. [1],Paakkari & Paakkari [16] and Nutbeam [15] were not explicitly mentioned in all the included publications, the descriptions could be related to the HL dimensions in these definitions.

The construct HL and how it is described in the literature is highly complex. Skills and competencies frequently intertwine, as evidenced by the quotes demonstrating co-occurrences within the descriptions of HL. Additionally, the description of HL learning outcomes often entails the use of intricate sentence structures, while measurement instruments utilize two-part questions for item presentation.

Learning outcomes are often constructed as tasks requiring the use of multiple skills. This is appropriate for the construct of HL, as several HL skills must be used simultaneously in daily health activities.

An educational setting was present in most of the included literature. This might indicate that the educational setting can be seen as an ideal place for children to learn HL skills as proposed by Kostenius and colleagues [44]. For example, included publications from China, Germany, Finland and the United States show the use of HL as a learning outcome in (health) educational programs and curricula aimed at improving health literacy. In the above-mentioned countries, the description of HL for specific grades was based on national educational standards and was measurable within the educational context. In the United States this is part of the *National Action Plan to Improve Health Literacy* [53]. In this plan [53], the third goal states: “*Incorporate accurate, standards-based, and developmentally appropriate health and science information and curricula in childcare and education through the university level”* (p.32). In Europe, we also see progress in the uptake of HL in school curricula where propositions are being made by Okan et al. [54] in *Health Literate Schools* and by Kirchhoff et al. [55] with their concept for the development of health-literate schools. They used the standards for a health-promoting school formulated by Schools for Health in Europe (SHE) [56] in which “*Standard 4: The school implements a health promotion curriculum to pupils”’* (p.18) and *“Standard 7: The school improves pupils health literacy”* (p.19–20) relate to HL and health promotion in the school context. The WHO and UNESCO published the Global Standards for Health Promoting Schools [57], with their common goal to make every school a health-promoting school. In a health-promoting educational system HL has a place, as the system entails allocation of a budget for health promotion in education and promotes health and well-being in the curriculum and teaching methods. Moreover, there is evidence that building the health assets of young people in the areas of social and emotional well-being at school can significantly improve educational outcomes [58]. This indicates an opportunity for HL to be adopted in learning outcomes of education where children learn the assets for a healthy life. Schools might be incentivized to adopt HL learning outcomes quicker when it leads to improvement in educational outcomes.

The information sources mentioned in the included publications were mostly internet and news, followed by parents or guardians. Noteworthy is the fact that although most settings were in school, teachers or school were rarely described as information sources. To implement HL learning outcomes in school curricula, there should be an emphasis on schools and teachers being able to understand as well as address HL in children within their teaching practices. Working on organizational HL could be helpful to start with in a school setting [55]. Organizational HL relates to including promotion of health in all policies and creating supportive environments for health.

Kirchhoff and colleagues [55] provide us with a set of eight standards that can be developed for school-related persons on four levels: organizational level, instructional level, school staff level and school environment level. It is important to notice the challenges in the school system in general and teachers specifically for adding new topics to a curriculum. Therefore, Kirchhoff et al. suggest that implementing HL in school should be considered during a time for change and development of new curricula, starting with including HL in the school mission and working from there towards learning outcomes, assessments, and instructional design with the school staff [55].

The health topics we found in the descriptions of HL were often formulated as risks or unhealthy aspects, for example: injury, sickness or nausea, alcohol/drinking or abuse, and rarely promoting healthy behavior. In the context of health promotion, we aim to focus on enabling people, individually and collectively, to increase control over the determinants of health. As argued in the report for promoting positive adolescent health behaviors and outcomes [59], we should not only prevent children from risk behavior, but they should also learn the skills to take healthy risks, which are needed for the development of a healthy life. When describing HL, more use should be made of health-promoting topics and behaviors. This is especially the case for younger school children, while unhealthy behaviors are widespread, such as excessive screen time, excessive consumption of sweetened beverages and high sugar/fat/salt snacks, only a small percentage of children aged 9–12 years are actively engaged in health risk behaviors as classified by the WHO such as alcohol/drug abuse or smoking.

### Strengths and limitations

This review was conducted following the PRISMA ScR Guidelines, using a pre-registered protocol. The search procedure was performed in collaboration with a medical librarian and the screening of eligible studies was performed in duplicate. To enhance the reliability of the methods, we used automated concept analysis and code co-occurrence in ATLAS.ti (version 22). The automated analysis was performed on carefully selected data describing HL. Therefor an automated analysis of concepts based on the occurrence of noun phrases provides insight into what topics were most used within the description of HL in the included data. The observed co-occurrence between the conceptualization of definitions and skills in the included publications were appropriate and in-line with previous definitions, confirming the soundness of our coding process and analyses. Although, the included types of publications were different, the extracted data was similar: descriptions of HL that we coded for the concept and co-occurrence analyses. Most importantly, our review adds novel information on how HL is described for younger children under the age of ten specifically in the educational standards [33, 49] and the publication from the publications of Knisel et al. [43]., and Yu et al. [50].

A limitation of this review is that one researcher performed the data extraction and analyses. During data extraction, descriptions of previously included instruments were not included twice, to not distort the data. The analysis and results were repeatedly discussed within the research team, after a check of the coded segments to ensure completeness and accuracy.

### Implications of the study

Due to the diversity of conceptualizations for this still evolving concept it could be relevant to develop a unified framework or consensus on defining, conceptualizing and describing HL in this specific age group within health promotion. Research in the educational setting on how HL could be incorporated in education is worth exploring, from different perspectives such as health and pedagogy.

Practical implications might be the integration of HL into the educational setting. This review provides schools and educational professionals with an overview of how HL is currently described applied in educational settings. Organizational HL [55] in schools could play a pivotal role in prioritizing health and HL in the educational system [54].

A collaborative approach between health and educational professionals, researchers and children themselves could facilitate the adequate integration of HL in the context of health promotion into school curricula.

## Conclusion

We found a wide variety of conceptualizations and descriptions of HL which could be linked to two commonly applied HL definitions by Sorenson et al. [1], Paakkari and Paakkari [16], and HL skills by Nutbeam et al. [15].

For health promotion, HL was mostly described as learning outcomes for school curricula or as assets and competencies necessary to make healthy choices. The skills needed for accessing, understanding, appraising, and applying health information can be learned and taught in schools. Incorporating HL as a learning outcome within educational programs can provide a structured and measurable approach to improving HL in children. The description of HL for children requires a foundation built upon suitable and established definitions that align with health promoting contexts, particularly within educational settings like schools. Taking a holistic approach to conceptualizing and describing HL is crucial, emphasizing the integration of diverse skills and competencies and making clear what is included and what not. By incorporating various topics, the description can effectively address all dimensions of HL, encompassing the common daily activities of children where they engage in health-promoting actions. This comprehensive approach ensures that children’s HL development includes all relevant dimensions, enabling them to navigate and make informed decisions regarding health promotion.

### Electronic supplementary material

Below is the link to the electronic supplementary material.

**Supplementary Material 1**: Preferred Reporting Items for Systematic reviews and Meta-Analyses extension for Scoping Reviews (PRISMA-ScR) Checklist

**Supplementary Material 2**: Standards for Reporting Qualitative Research (SRQR)

**Supplementary Material 3**: Glossary

**Supplementary Material 4**: Search strategies

## Data Availability

The data used and analyzed was obtained from the included publications in this scoping review. A dataset was created with selected data from the included publications as described in the methods section. The dataset is available from the corresponding author on reasonable request.

## Abbreviations (Terms used in this work are defined in a glossary in Appendix A.

HL: Health literacy
IS: Ivy Sanders
KC: Kristina Chepak
KJ: Katarina Jerković-Ćosić
LS: Linda Schoonmade
MC: Mai Chin A Paw
MM: Marijke Mol
ScR: Scoping Review
WHO: World Health Organization
WvB: Wieke van Boxtel
