# Supplementary material for: Health literacy in the context of child health promotion: a scoping review of conceptualizations and descriptions
Source: BMC Public Health. 2024 Mar 14;24:808. doi: 10.1186/s12889-024-17955-7 (PMC10941366; doi:10.1186/s12889-024-17955-7)
Supplement: Supplementary file 3 — Supplementary Material 3: Glossary [file 12889_2024_17955_MOESM3_ESM.docx]

### Appendix A: Glossary

Based on The Health Promotion Glossary [1], Cambridge dictionary [2]

**Construct Health Literacy:** Health literacy represents the personal knowledge and competencies that accumulate through daily activities, social interactions and across generations. Personal knowledge and competencies are mediated by the organizational structures and availability of resources that enable people to access, understand, appraise and use information and services in ways that promote and maintain good health and well-being for themselves and those around them.

**Health promotion:** is the process of enabling people, individually and collectively, to increase control over the determinants of health and thereby improve their health.

**Context of health promotion**: Within the context of health promotion, health has been considered as a resource that permits people to lead individually, socially and economically productive lives

**Primary prevention:** is directed towards lowering the prevalence of risk factors common to a range of diseases (such as tobacco and alcohol use, obesity and high blood pressure) in order to prevent the initial occurrence of a disorder, for example through behavior change advice.”.

**Setting:** Health literacy development is undertaken across all settings where people’s knowledge, understanding and behavior about health can be influenced. This includes prenatal environments, people’s homes, villages and cities, schools and workplaces – that is, all the places where people are exposed to health-related information and where their health behaviors may be influenced.

**Settings for health**: The place or social context where people engage in daily activities, in which environmental, organizational and personal factors interact to affect health and well-being.

**Construct:** Constructs are broad concepts or topics for a study. Constructs can be conceptually defined in that they have meaning in theoretical terms. They can be abstract and do not necessarily need to be directly observable. Health Literacy is a multi dimensional construct.

**Content:**

- Topic: the subject which is used/described or assessed
- Term: specific words that are used in the description
- Concept: for data analysis based on noun phrases found in text which indicate a similar topic
- Competency: an important skill that is needed to do a task or action
- Skill: a particular ability that you develop through training and experience and that is useful in a specific task or action:
- Learning outcome: statements of the knowledge, skills and abilities individuals should possess and can demonstrate upon completion of a learning experience or sequence of learning experiences.
- Operationalization: process whereby researchers specify empirical concepts that can be taken as indicators of the attributes of a concept

[1] World Health Organization. Health promotion glossary of terms 2021 2021.

[2] [Cambridge Dictionary | English Dictionary, Translations & Thesaurus](https://dictionary.cambridge.org/) retrieved 15-12-2022

## Appendix B: Data extraction tables

Study characteristics and content

| Study ID | Title | Year of publication | Country | Aim of study | Study design | Study methods/data collection | Data preparation/analysis | Population description | Method of recruitment of participants | Total number of participants |
| --- | --- | --- | --- | --- | --- | --- | --- | --- | --- | --- |
|  |  |  |  |  |  |  |  |  |  |  |

Construct and content

| Construct | Levels in HL | Context | Setting | Content subjects | Content description |
| --- | --- | --- | --- | --- | --- |
|  |  |  |  |  |  |

Measurement instrument

| Measurement instrument developed | Measurement instrument name | Mode of administration | Target Population | Population age | N items | Response options | Range of scoring | Language | Validity/Reliability |
| --- | --- | --- | --- | --- | --- | --- | --- | --- | --- |
|  |  |  |  |  |  |  |  |  |  |

# Appendix C Code group and code distribution

Table A1: Distribution of code groups with imbedded codes for all content based on competences definition (orange), learning outcome definition (green), HL levels (blue), health topics (white). Grounded indicates the number of times the codes are used for the co content. The Grounded total is the number of times multiple codes per group are coded on one quotation.

| **Code Groups** | | **Code** | **Grounded** | **Grounded total** |
| --- | --- | --- | --- | --- |
| ● **Construct HL Competences definition** based on Sørensen et al. (2012) | | ● Accessing | 34 | 222 |
|  |  | ● Understanding | 97 |  |
|  |  | ● Appraising | 53 |  |
|  |  | ● Applying | 68 |  |
| ● **Construct HL Learning outcome definition**  based Paakkari & Paakkari (2012) | | ● Theoretical knowledge | 46 | 202 |
|  |  | ● Practical knowledge | 67 |  |
|  |  | ● Critical thinking | 71 |  |
|  |  | ● Self-awareness | 48 |  |
|  |  | ● Citizenship | 53 |  |
| ● **HL Levels**  based on Nutbeam (2008) | **Functional skills** | ● Reading | 12 | 22 |
|  |  | ● Pronunciation | 2 |  |
|  |  | ● Writing | 1 |  |
|  |  | ● Numeracy | 12 |  |
|  | **Cognitive skills** | ● Knowledge | 99 | 197 |
|  |  | ● Comprehension | 80 |  |
|  |  | ● Extracting information | 34 |  |
|  |  | ● Derive meaning | 70 |  |
|  | **Interactive skills** | ● Communication | 58 | 58 |
|  | **Critical skills** | ● Analyzing information (critically) | 78 | 145 |
|  |  | ● Decision making | 32 |  |
|  |  | ● Use/apply information | 86 |  |
| ○ **Health Topics** | | ○ Health topics | 22 | 28 |
|  |  | ○ Health risks | 4 |  |
|  |  | ○ Health related experiences | 2 |  |
|  |  | ○ Health socialization subjects | 1 |  |
| **Other codes** | | Caring and confirming | 3 | 15 |
|  |  | Engaging and empowering | 3 |  |
|  |  | Goal setting | 1 |  |
|  |  | Help-seeking behavior | 1 |  |
|  |  | Motivation | 2 |  |
|  |  | Problem solving | 2 |  |
|  |  | Recognition | 1 |  |
|  |  | Satisfaction | 1 |  |
|  |  | Stress management | 1 |  |

# Appendix D Settings and contexts

The frequencies of coded content within specific settings are presented in Table A2 and for contexts in Table A3.

Table A2: Quotation count of content related to the aspects of definitions and HL levels in specific settings

|  | **School** | **After school** | **Daily life/ Public health** | **Health Education Center** | **Multiple Settings** | **Public setting** | **Totals** |
| --- | --- | --- | --- | --- | --- | --- | --- |
| **HL construct Competences definition** | 152 | 29 | 25 | 10 | 5 | 1 | 222 |
| **HL construct Learning outcome definition** | 157 | 2 | 30 | 10 | 3 | 0 | 202 |
| **HL skills Cognitive** | 5 | 4 | 0 | 0 | 4 | 9 | 22 |
| **HL skills Critical** | 147 | 21 | 16 | 6 | 1 | 6 | 197 |
| **HL skills Functional** | 47 | 3 | 7 | 0 | 1 | 0 | 58 |
| **HL skills Interactive** | 119 | 13 | 11 | 1 | 1 | 0 | 145 |
| **Totals** | 627 | 72 | 89 | 27 | 15 | 16 | 846 |

Table A3: Quotation count of content related to the aspects of definitions and HL levels in specific contexts

|  | **Health  promotion** | **Multiple contexts** | **Totals** |
| --- | --- | --- | --- |
| **HL construct Competences definition** | 196 | 26 | 222 |
| **HL construct Learning outcome definition** | 192 | 10 | 202 |
| **HL skills Cognitive** | 18 | 4 | 22 |
| **HL skills Critical** | 149 | 48 | 197 |
| **HL skills Functional** | 51 | 7 | 58 |
| **HL skills Interactive** | 117 | 28 | 145 |
| **Totals** | 723 | 123 | 846 |

# Appendix E Health topics and information sources

Table A4: Health topics used in content

| **Topic** | **Word** | **Counted** |  | **Topic** | **Word** | **Counted** |  | **Topic** | **Word** | **Counted** |
| --- | --- | --- | --- | --- | --- | --- | --- | --- | --- | --- |
| *concept* | health/healthy/unhealthy | 42 |  | *Physical* | physical | 7 |  | *Disease* | disease | 5 |
|  | prevention | 10 |  |  | injury | 5 |  |  | asthma | 4 |
|  | activity | 5 |  |  | sick | 1 |  |  | anemia | 2 |
|  | development | 5 |  |  | nausea | 1 |  |  | diabetes | 2 |
|  | growth | 5 |  |  | fatigue | 1 |  |  | exam/examine | 4 |
|  | conceptualization | 4 |  |  | vision | 1 |  |  | obesity/fat | 3 |
|  | mental | 5 |  |  | oral/ dental/ tooth/ brush/brushing | 7 |  |  | bronchial | 1 |
|  | safety | 3 |  |  | body | 7 |  |  | cerebral | 1 |
|  | education | 4 |  |  | sex/sexual/sexuality/std's | 5 |  |  | depression | 1 |
|  | emotional | 4 |  | *Action* | food/nutrition/diet/ eating/breakfast/snack | 18 |  |  | dyslexia | 1 |
|  | social | 4 |  |  | personal hygiene | 8 |  |  | hemophilia | 1 |
|  | care | 2 |  |  | alcohol/drinking | 6 |  |  | HIV | 1 |
|  | community | 2 |  |  | drugs/substance | 6 |  |  | pregnancy | 1 |
|  | disparity | 2 |  |  | tobacco/cigarette smoking | 4 |  |  | stress | 1 |
|  | environmental | 2 |  |  | abuse | 5 |  |  | medication | 1 |
|  | emergency | 1 |  |  |  |  |  |  | tetanus | 1 |

Table A5: Information sources used in content

| **Topic** | **Word** | **Counted** |  | **Topic** | **Word** | **Counted** |
| --- | --- | --- | --- | --- | --- | --- |
| *Information sources* | internet/websites/page/google | 9 |  | *Information sources* | friend | 2 |
|  | news/broadcast/newscast | 4 |  |  | advertising | 2 |
|  | parent/guardian/family | 3 |  |  | sibling | 1 |
|  | American heart association/FDA/NIH | 3 |  |  | radio | 1 |
|  | television | 2 |  |  | programs | 1 |
|  | teacher/ coach | 2 |  |  | print | 1 |
|  | physician/pharmacist | 2 |  |  | article | 1 |
|  | pamphlets/prints | 2 |  |  | specific tv shows | 1 |
|  | magazine | 2 |  |  |  |  |

# Appendix F Code Co-occurrence

| **●** HL Construct Competences definition  **●** HL construct Learning outcome definition  **●** HL Levels | **● Accessing** | **● Understanding** | **● Appraising** | **● Applying** | **● Theoretical knowledge** | **● Practical knowledge** | **● Critical thinking** | **● Self-awareness** | **● Citizenship** | **●Reading** | **●Pronunciation** | **●Writing** | **●Numeracy** | **●Knowledge** | **●Comprehension** | **●Extracting information** | **●Derive meaning** | **●Communication** | **●Analyzing information** | **●Decision making** | **●Use/apply information** |
| --- | --- | --- | --- | --- | --- | --- | --- | --- | --- | --- | --- | --- | --- | --- | --- | --- | --- | --- | --- | --- | --- |
| **● Accessing** |  |  |  |  |  |  |  |  |  |  |  |  |  |  |  |  |  |  |  |  |  |
| **● Understanding** | 4 |  |  |  |  |  |  |  |  |  |  |  |  |  |  |  |  |  |  |  |  |
| **● Appraising** | 10 | 2 |  |  |  |  |  |  |  |  |  |  |  |  |  |  |  |  |  |  |  |
| **● Applying** | 2 | 11 | 3 |  |  |  |  |  |  |  |  |  |  |  |  |  |  |  |  |  |  |
| **● Theoretical knowledge** | 4 | 20 | 8 | 6 |  |  |  |  |  |  |  |  |  |  |  |  |  |  |  |  |  |
| **● Practical knowledge** | 11 | 17 | 8 | 26 | 8 |  |  |  |  |  |  |  |  |  |  |  |  |  |  |  |  |
| **● Critical thinking** | 9 | 12 | 25 | 14 | 18 | 23 |  |  |  |  |  |  |  |  |  |  |  |  |  |  |  |
| **● Self-awareness** | 3 | 13 | 3 | 15 | 2 | 14 | 10 |  |  |  |  |  |  |  |  |  |  |  |  |  |  |
| **● Citizenship** |  | 13 | 3 | 12 | 7 | 8 | 9 | 4 |  |  |  |  |  |  |  |  |  |  |  |  |  |
| **● Reading** |  | 3 |  |  |  |  |  |  |  |  |  |  |  |  |  |  |  |  |  |  |  |
| **● Pronunciation** |  |  |  | 1 |  | 1 | 1 |  |  |  |  |  |  |  |  |  |  |  |  |  |  |
| **● Writing** |  |  |  | 1 |  | 1 | 1 |  |  |  | 1 |  |  |  |  |  |  |  |  |  |  |
| **● Numeracy** |  | 1 |  |  |  |  |  |  |  | 4 |  |  |  |  |  |  |  |  |  |  |  |
| **● Knowledge** | 3 | 48 | 7 | 10 | 13 | 8 | 13 | 9 | 4 |  | 1 | 1 |  |  |  |  |  |  |  |  |  |
| **● Comprehension** | 5 | 38 | 9 | 8 | 13 | 8 | 16 | 7 | 7 | 4 |  |  |  | 36 |  |  |  |  |  |  |  |
| **● Extracting information** | 11 | 5 | 11 |  | 2 | 4 | 8 |  | 1 | 2 |  |  | 2 | 9 | 5 |  |  |  |  |  |  |
| **● Derive meaning** | 7 | 25 | 18 | 8 | 7 | 8 | 19 | 8 | 8 | 1 | 1 | 1 |  | 18 | 20 | 6 |  |  |  |  |  |
| **● Communication** | 1 | 11 | 1 | 12 | 2 | 13 | 8 | 13 | 15 |  |  |  |  | 2 | 12 | 1 | 5 |  |  |  |  |
| **● Analyzing information** | 5 | 14 | 31 | 14 | 11 | 17 | 36 | 7 | 14 |  |  |  |  | 12 | 10 | 11 | 23 | 4 |  |  |  |
| **● Decision making** |  | 5 | 1 | 13 | 1 | 4 | 7 | 7 | 1 |  |  |  |  | 8 | 3 | 1 | 2 | 1 | 9 |  |  |
| **● Use/apply information** | 1 | 16 | 5 | 34 | 1 | 25 | 17 | 12 | 11 |  |  |  |  | 16 | 11 | 2 | 6 | 15 | 23 | 26 |  |

Table A6: Code co-occurrence frequencies for all codes in definitions and HL levels, the larger the number to more it was found in co-occurrence in the publications.

| **●** HL Construct Competences definition  **●** HL construct Learning outcome definition  **●** HL Levels | **● Accessing** | **● Understanding** | **● Appraising** | **● Applying** | **● Theoretical knowledge** | **● Practical knowledge** | **● Critical thinking** | **● Self-awareness** | **● Citizenship** | **●Reading** | **●Pronunciation** | **●Writing** | **●Numeracy** | **●Knowledge** | **●Comprehension** | **●Extracting information** | **●Derive meaning** | **●Communication** | **●Analyzing information (critically)** | **●Decision making** | **●Use/apply information** |
| --- | --- | --- | --- | --- | --- | --- | --- | --- | --- | --- | --- | --- | --- | --- | --- | --- | --- | --- | --- | --- | --- |
| **● Accessing** |  |  |  |  |  |  |  |  |  |  |  |  |  |  |  |  |  |  |  |  |  |
| **● Understanding** |  |  |  |  |  |  |  |  |  |  |  |  |  |  |  |  |  |  |  |  |  |
| **● Appraising** |  |  |  |  |  |  |  |  |  |  |  |  |  |  |  |  |  |  |  |  |  |
| **● Applying** |  |  |  |  |  |  |  |  |  |  |  |  |  |  |  |  |  |  |  |  |  |
| **● Theoretical knowledge** |  |  |  |  |  |  |  |  |  |  |  |  |  |  |  |  |  |  |  |  |  |
| **● Practical knowledge** |  |  |  |  |  |  |  |  |  |  |  |  |  |  |  |  |  |  |  |  |  |
| **● Critical thinking** |  |  |  |  |  |  |  |  |  |  |  |  |  |  |  |  |  |  |  |  |  |
| **● Self-awareness** |  |  |  |  |  |  |  |  |  |  |  |  |  |  |  |  |  |  |  |  |  |
| **● Citizenship** |  |  |  |  |  |  |  |  |  |  |  |  |  |  |  |  |  |  |  |  |  |
| **● Reading** |  |  |  |  |  |  |  |  |  |  |  |  |  |  |  |  |  |  |  |  |  |
| **● Pronunciation** |  |  |  |  |  |  |  |  |  |  |  |  |  |  |  |  |  |  |  |  |  |
| **● Writing** |  |  |  |  |  |  |  |  |  |  |  |  |  |  |  |  |  |  |  |  |  |
| **● Numeracy** |  |  |  |  |  |  |  |  |  |  |  |  |  |  |  |  |  |  |  |  |  |
| **● Knowledge** |  |  |  |  |  |  |  |  |  |  |  |  |  |  |  |  |  |  |  |  |  |
| **● Comprehension** |  |  |  |  |  |  |  |  |  |  |  |  |  |  |  |  |  |  |  |  |  |
| **● Extracting information** |  |  |  |  |  |  |  |  |  |  |  |  |  |  |  |  |  |  |  |  |  |
| **● Derive meaning** |  |  |  |  |  |  |  |  |  |  |  |  |  |  |  |  |  |  |  |  |  |
| **● Communication** |  |  |  |  |  |  |  |  |  |  |  |  |  |  |  |  |  |  |  |  |  |
| **● Analyzing information (critically)** |  |  |  |  |  |  |  |  |  |  |  |  |  |  |  |  |  |  |  |  |  |
| **● Decision making** |  |  |  |  |  |  |  |  |  |  |  |  |  |  |  |  |  |  |  |  |  |
| **● Use/apply information** |  |  |  |  |  |  |  |  |  |  |  |  |  |  |  |  |  |  |  |  |  |

Table A7: Code co-occurrence for all codes in definitions and HL levels, green indicate code co-occurring more than 20 times, light green co-occurrence less than 20 times and in red no co-occurrence of the codes.

# appendix G Topics in content

Figure A1: Word cloud of topics for all publications

**
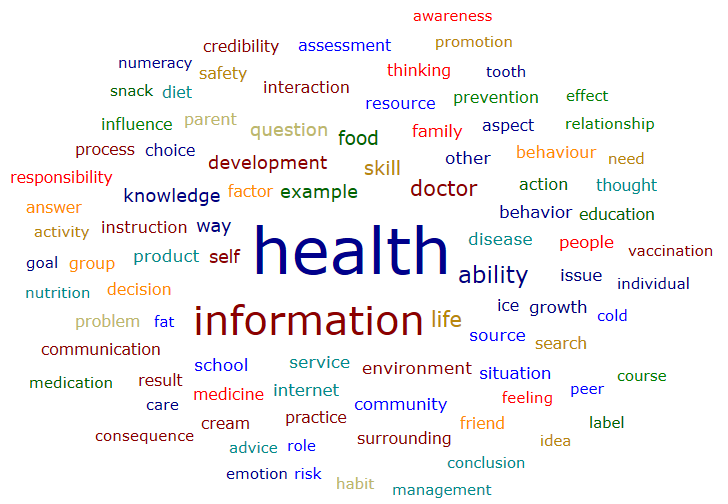
**

**Most frequent topics:**

Table A8: Most frequent topics Health and Information with frequency and noun phrases for all publications

| **Concept** | **Frequency** | **Noun Phrases** |
| --- | --- | --- |
| Health | 363 | "health (81)" "health information (34)" "health-related information (18)" "your health (18)" "personal health (14)" "health issues (12)" "health promotion (12)" "health behaviors (8)" "health practices (8)" "health problems (8)" "health resources (8)" "lifelong health (8)" "community and environmental health issues (7)" "mental health (7)" "one's health (7)" "their health (7)" "health-related decisions (6)" "community and environmental health plans/strategies (5)" "health topic (5)" "various health plans/strategies (5)" "good health (4)" "health habits (4)" "health services (4)" "personal and family health decisions (4)" "the 4 health education settings (4)" "community health (3)" "health agencies (3)" "health care (3)" "health-related life situations (3)" "health risks (3)" "personal health decisions (3)" "basic health knowledge (2)" "basic health skills (2)" "consumer health (2)" "environmental health (2)" "everyday health habits (2)" "health care requirements (2)" "health environment (2)" "health literate (2)" "health question (2)" "our health (2)" "personal health goals (2)" "public health policies (2)" "personal health progress (2)" "suitable health products (2)" "their health literacy (2)" "their own good health (2)" |
| Information | 148 | "information (47)" "health information (25)" "health-related information (17)" "good information (4)" "valid health information (4)" "accessing information (3)" "the written information (3)" "what health information (3)" "accurate and meaningful health information (2)" "appropriate information ethics (2)" "consumer information (2)" "different information (2)" "information/data (2)" "information need (2)" "information sources (2)" "new information (2)" "reliable information (2)" "synthesize information (2) "wrong information (2)" |

Table A9: Top 3 topics per code

| **Codes** | **Top 3 Topics (frequency)** |
| --- | --- |
| **● Accessing** | Service (12), Products (8), Sources (5) |
| **● Understanding** | Development (17), Food (17), Skill (15) |
| **● Appraising** | Service (14), Product (14), Practice (11) |
| **● Applying** | Situation (11), Way (10), Community (8) |
| **● Theoretical knowledge** | Development (13), Life (9), Growth (9) |
| **● Practical knowledge** | Behavior (16), Situation (10), Service (9) |
| **● Critical thinking** | Services (18), Product (17), Disease (13) |
| **● Self-awareness** | Self (10), Feeling (7), Behavior (6) |
| **● Citizenship** | Community (30), Family (17), Environment (12) |
| **● Functional HL** | Ice Cream (11), Calorie (7), Fat (7) |
| **● Cognitive HL** | Development (20), Life (18), Disease (17) |
| **● Interactive HL** | Communication (16), Skill (12), Interaction (11) |
| **● Critical HL** | Service (18), Product (18), Community (18) |

*Table A10: Frequency of the topics found as concepts in noun phrases of the content in all code groups through automatic concept analysis in Atlas.Ti, presented* *in white rows above frequency cut off point, grey rows below frequency cut off.*

| **Concept** | **Frequency** | **Noun Phrases** |
| --- | --- | --- |
| health | 297 | "health (55)" "health information (17)" "your health (16)" "the health (11)" "health promotion (8)" "health-related information (8)" "life-long health (8)" "their health (7)" "mental health (5)" "health habits (4)" "personal health (4)" "the 4 health education settings (4)" "the health information (4)" "good health (3)" "health-related life situations (3)" "personal health decisions (3)" "valid health information (3)" "community and environmental health issues (2)" "community and environmental health plans/strategies (2)" "community health issues (2)" "community health plans (2)" "consumer health (2)" "environmental health (2)" "everyday health habits (2)" "health care (2)" "health care behaviors (2)" "health care requirements (2)" "health environment (2)" "health issues (2)" "health practices (2)" "health products (2)" "health promoting behaviours (2)" "health services (2)" "health-enhancing behaviors (2)" "how appropriate and inappropriate health practices (2)" "one's health (2)" "our health (2)" "past health plans/strategies (2)" "personal and family health decisions (2)" "personal health behaviors (2)" "personal health goals (2)" "personal health progress (2)" "public health policies (2)" "regional health agencies (2)" "suitable health products (2)" "that influence health and health behaviors (2)" "their health literacy (2)" "their own good health (2)" "various community health issues (2)" "various health issues (2)" "various health plans/strategies (2)" "a health agency (1)" "accurate and meaningful health information (1)" "accurate health information (1)" "adolescent health problems (1)" "age-appropriate health-related vocabulary (1)" "available health-related information (1)" "b) process health information (1)" "basic health knowledge (1)" "common childhood health problems (1)" "community health (1)" "community health advocacy activities (1)" "critically appraising health information (1)" "dental health literacy (1)" "different health plans (1)" "equitable health care (1)" "good health care (1)" "good personal health (1)" "health advocacy (1)" "health advocacy strategies (1)" "health behaviors (1)" "health behaviours (1)" "health communications (1)" "health concepts (1)" "health goals (1)" "health health mapping (1)" "health information project competition (1)" "health knowledge (1)" "health literacy milestones (1)" "health literacy skills (1)" "health literate (1)" "health pressures (1)" "health promoting products (1)" "health related interactions (1)" "health resources (1)" "health risks (1)" "health skills (1)" "health-literate individuals (1)" "health-promoting foods (1)" "health-related actions (1)" "health-related decision-making (1)" "health-related decisions (1)" "health-related knowledge (1)" "health-related services (1)" "how social, emotional, and physical health (1)" "major health issues (1)" "mental and emotional health (1)" "mental, emotional, social, and physical health (1)" "my health examination report (1)" "non-health-promoting foods (1)" "personal and family health (1)" "poor eyesight and good oral health (1)" "positive health practices (1)" "professional health services (1)" "regional health resources (1)" "significant community health issues (1)" "the health topics (1)" "the health-related consequences (1)" "the provided health information (1)" "the term health (1)" "their own health decisions (1)" "their sexual health (1)" "true and false health information (1)" "what health information (1)" "what specific health information (1)" "your health relate (1)" "your own health (1)" |
| information | 86 | "information (25)" "health information (17)" "health-related information (8)" "the health information (4)" "valid health information (3)" "consumer information (2)" "information sources (2)" "information/data (2)" "new information (2)" "reliable information (2)" "accurate and meaningful health information (1)" "accurate health information (1)" "available health-related information (1)" "b) process health information (1)" "contact information (1)" "critically appraising health information (1)" "extract information (1)" "good information (1)" "health information project competition (1)" "the information (1)" "the most wrong information (1)" "the provided health information (1)" "this information (1)" "true and false health information (1)" "valid and reliable information (1)" "what health information (1)" "what information (1)" "what specific health information (1)" "which information (1)" |
| pupil | 55 | "pupils (48)" "pupils opportunities (2)" "pupils’ ability (2)" "pupils’ environmental awareness (2)" "grade 4-6"to guide pupils (1)" |
| student | 50 | "students (41)" "the student (8)" "a “fitness-pyramid” students (1)" |
| community | 42 | "community (11)" "communities (5)" "a community (2)" "community and environmental health issues (2)" "community and environmental health plans/strategies (2)" "community health issues (2)" "community health plans (2)" "local communities (2)" "the community (2)" "various community (2)" "various community health issues (2)" "a beautiful community (1)" "a healthy community (1)" "community health (1)" "community health advocacy activities (1)" "community-based efforts (1)" "our community (1)" "our community environment (1)" "significant community health issues (1)" |
| life | 40 | "life (9)" "daily life (4)" "life courses (4)" "life-long health (4)" "health-related life situations (3)" "life course stages (2)" "life events (2)" "real life (2)" "their lives (2)" "their own lives (2)" "also people’s life courses (1)" "life cycle (1)" "life-threatening diseases (1)" "the social life (1)" "their life (1)" "your everyday life (1)" |
| food | 38 | "which food (6)" "healthy food (4)" "food (3)" "different food groups (2)" "food shopping (2)" "foods (2)" "the “food pyramid (2)" "the foods (2)" "their regulary consumed food (2)" "all healthy foods (1)" "fake food (1)" "food choices (1)" "food options (1)" "food-impulses (1)" "health-promoting foods (1)" "non-health-promoting foods (1)" "processed foods (1)" "right balanced food (1)" "some foods (1)" "the related foods (1)" "the two food categories (1)" "various foods (1)" |
| grade | 38 | "grade (29)" "citizenship grade (2)" "citizinship grade (2)" "critical thinking grade (2)" "grade formulation (2)" "grade 4-6"to guide pupils (1)" |
| skill | 36 | "skill (6)" "skills (6)" "proper rejection skills (3)" "communication skills (2)" "different coping skills (2)" "effective emotional management skills (2)" "effective interpersonal communication skills (2)" "environmental protection skills (2)" "medication-taking skills (2)" "their emotional and interaction skills (2)" "effective communication skills (1)" "first aid skills (1)" "first-aid skills (1)" "health literacy skills (1)" "health skills (1)" "interpersonal and decision-making skills (1)" "interpersonal communication skills (1)" |
| way | 35 | "ways (17)" "the way (7)" "the ways (3)" "healthy ways (2)" "various ways (2)" "a way (1)" "model appropriate ways (1)" "my way (1)" "possible ways (1)" |
| behavior | 35 | "behaviors (8)" "health care behaviors (2)" "health-enhancing behaviors (2)" "personal behavior (2)" "personal health behaviors (2)" "risk taking behaviors (2)" "risky and harmful behavior (2)" "risky and harmful behaviors (2)" "age-appropriate behaviors (1)" "behaviors/situations (1)" "everyday behavior (1)" "health behaviors (1)" "healthcare behaviors (1)" "individual behavior (1)" "individual behaviors (1)" "negative and positive behaviors (1)" "one's own behavior (1)" "positive, personal hygienic behaviors (1)" "that influence health and health behaviors (1)" "their behaviors (1)" "your behavior (1)" |
| example | 31 | "examples (19)" "example (6)" "an example (2)" "any other examples (2)" "at least 2 examples (1)" "at least 3 examples (1)" |
| development | 30 | "development (16)" "sustainable development (4)" "the development (3)" "particularly development (2)" "growth development (1)" "individual development (1)" "mental and emotional development (1)" "the expected development (1)" "the item development process (1)" |
| child | 29 | "children (12)" "the children (10)" "child (2)" "child abuse (1)" "each child (1)" "emerging child autonomy (1)" "other children (1)" "younger children (1)" |
| service | 28 | "services (22)" "health services (2)" "health-related services (1)" "professional health services (1)" "professional services (1)" "service navigation (1)" |
| hl | 27 | "functional hl (9)" "interactive hl (8)" "critical hl (7)" "communicative hl (1)" "self-worth (functional hl (1)" "vaccination hl (1)" |
| situation | 26 | "situations (6)" "health-related life situations (3)" "potentially dangerous situations (3)" "different conflict and crisis situations (2)" "interactive situations (2)" "potentially harmful situations (2)" "the situation (2)" "age-appropriate situations (1)" "behaviors/situations (1)" "conflict situations (1)" "everyday situations (1)" "various personal situations (1)" "what everyday situations (1)" |
| other | 24 | "others (23)" "us/others (1)" |
| doctor | 24 | "the doctor (10)" "your doctor (9)" "a doctor (2)" "doctors (2)" "doctor (1)" |
| product | 23 | "products (16)" "health products (2)" "suitable health products (2)" "a yogurt product (1)" "health promoting products (1)" "this product (1)" |
| family | 23 | "family (11)" "their families (4)" "families (3)" "personal and family health decisions (2)" "family members (1)" "personal and family health (1)" "the family (1)" |
| school | 23 | "school (12)" "schools (3)" "school environment (2)" "action-oriented school project (1)" "middle school (1)" "school map (1)" "the school (1)" "the school environment (1)" "the school sports meet (1)" |
| environment | 22 | "the environment (5)" "environment (3)" "environments (2)" "health environment (2)" "school environment (2)" "the learning environment (2)" "the living environment (2)" "our community environment (1)" "the future?school environment (1)" "the school environment (1)" "the surroundings natural environment (1)" |
| disease | 22 | "disease prevention (9)" "disease (6)" "infectious disease (2)" "common infectious diseases (1)" "disease prevention concepts (1)" "diseases (1)" "life-threatening diseases (1)" "spread/prevent diseases (1)" |
| parent | 22 | "your parents (15)" "my parents (4)" "parents (2)" "their parents (1)" |
| practice | 20 | "practices (11)" "health practices (2)" "how appropriate and inappropriate health practices (2)" "personal practices (2)" "b)examine practices (1)" "positive health practices (1)" "support practices (1)" |
| communication | 19 | "communication (7)" "communication skills (2)" "effective communication (2)" "effective interpersonal communication skills (2)" "appropriate communication techniques (1)" "effective communication skills (1)" "health communications (1)" "healthy communication and resolution strategies (1)" "interpersonal communication skills (1)" "various communication techniques (1)" |
| scenario | 19 | "scenario (19)" |
| interaction | 18 | "interpersonal interactions (8)" "the interaction (5)" "social interaction (2)" "their emotional and interaction skills (2)" "health related interactions (1)" |
| ability | 17 | "ability (10)" "the ability (5)" "pupils’ ability (2)" |
| growth | 17 | "growth (13)" "individual growth (2)" "growth development (1)" "physical, mental,emotional, and social growth (1)" |
| diet | 17 | "a healthy diet (6)" "diet (4)" "your diet (3)" "a healthy, balanced diet (2)" "diet choices (2)" |
| Pupils | 17 | "pupils (17)" |
| self | 17 | "self (6)" "a healthy self-concept (2)" "self-assessment (2)" "self-confidence (2)" "self-control (2)" "a self-directed learner (1)" "self-awareness (1)" "self-worth (functional hl (1)" |
| ice | 16 | "ice cream (6)" "how much ice cream (4)" "this ice cream (4)" "this ice cream?skill (2)" |
| safety | 16 | "safety (8)" "road safety lessons (6)" "outdoor safety tips (1)" "safety tips (1)" |
| role | 16 | "the role (6)" "different roles (4)" "their own role (2)" "a positive, active role (1)" "role-playing games (1)" "the roles (1)" "tobacco-smoking?which role (1)" |
| issue | 15 | "community and environmental health issues (2)" "community health issues (2)" "health issues (2)" "various community health issues (2)" "various health issues (2)" "a tough issue (1)" "major health issues (1)" "significant community health issues (1)" "this issue (1)" "tough issues (1)" |
| feeling | 15 | "feelings (8)" "enhanced feelings (2)" "positive feelings (2)" "the feeling (2)" "your feelings (1)" |
| strategy | 15 | "strategies (3)" "community and environmental health plans/strategies (2)" "past health plans/strategies (2)" "various health plans/strategies (2)" "appropriate conflict resolution strategies (1)" "correct prevention strategy (1)" "effective conflict resolution strategies (1)" "health advocacy strategies (1)" "healthy communication and resolution strategies (1)" "your coping strategies (1)" |
| aspect | 15 | "aspects (8)" "another aspect (2)" "the various aspects (2)" "the aspects (1)" "the positive and negative aspects (1)" "those aspects (1)" |
| people | 14 | "other people (8)" "people (3)" "all people (1)" "also people’s life courses (1)" "different people (1)" |
| medicine | 14 | "your medicine (12)" "medicine (1)" "medicines (1)" |
| group | 14 | "a group (5)" "different food groups (2)" "groups (2)" "each group (1)" "peer group (1)" "the group (1)" "the group relay race (1)" "their groups (1)" |
| influence | 14 | "the influence (10)" "their influence (2)" "that influence health and health behaviors (1)" "what influences (1)" |
| prevention | 14 | "disease prevention (9)" "the prevention (2)" "correct prevention strategy (1)" "disease prevention concepts (1)" "prevention (1)" |
| cream | 14 | "ice cream (6)" "how much ice cream (4)" "this ice cream (4)" |
| action | 13 | "actions (3)" "the action (2)" "action (1)" "action-oriented school project (1)" "ethically responsible actions (1)" "health-related actions (1)" "one's own actions (1)" "these actions (1)" "this action area (1)" "which action (1)" |
| plan | 13 | "community and environmental health plans/strategies (2)" "community health plans (2)" "how different plans (2)" "past health plans/strategies (2)" "various health plans/strategies (2)" "various plans (2)" "different health plans (1)" |
| decision | 13 | "personal health decisions (3)" "personal and family health decisions (2)" "decisions (1)" "health-related decision-making (1)" "health-related decisions (1)" "interpersonal and decision-making skills (1)" "our decision-making collective?is (1)" "our final decision (1)" "their individual decision-making (1)" "their own health decisions (1)" |
| choice | 13 | "choices (4)" "diet choices (2)" "food choices (2)" "healthy choices (2)" "one's own choices (1)" "personal choices (1)" "your snack choices (1)" |
| resource | 13 | "resources (4)" "a resource (2)" "a resource-based manner (2)" "the resources (2)" "electronic resources (1)" "health resources (1)" "regional health resources (1)" |
| age | 12 | "3 age (2)" "3 age 8-9, multiply 2-digit number (2)" "4 age (2)" "age (2)" "2 age (1)" "age-appropriate behaviors (1)" "age-appropriate health-related vocabulary (1)" "age-appropriate situations (1)" |
| vaccination | 12 | "vaccinations (5)" "vaccination (4)" "oral vaccination/injection (1)" "the vaccination card (1)" "vaccination hl (1)" |
| behaviour | 12 | "behaviour (5)" "health promoting behaviours (2)" "polite behaviour (2)" "your behaviour (2)" "health behaviours (1)" |
| peer | 12 | "peers (9)" "peer group (1)" "peer relationships (1)" "their peers (1)" |
| care | 12 | "care (3)" "health care (2)" "health care behaviors (2)" "health care requirements (2)" "c) care (1)" "equitable health care (1)" "good health care (1)" |
| relationship | 12 | "causal relationships (4)" "relationships (3)" "healthy relationships (2)" "peer relationships (1)" "positive relationships (1)" "the relationship (1)" |
| knowledge | 12 | "knowledge (5)" "theoretical knowledge (2)" "basic health knowledge (1)" "health knowledge (1)" "health-related knowledge (1)" "practical knowledge (1)" "the knowledge (1)" |
| cold | 12 | "a cold (11)" "my cold (1)" |
| activity | 12 | "physical activity (6)" "community health advocacy activities (1)" "different activities (1)" "leisure activities (1)" "our leisure activities (1)" "sexual activity (1)" "your daily activities (1)" |
| fat | 11 | "saturated fat (9)" "fat (2)" |
| conflict | 10 | "conflicts (3)" "conflict (2)" "different conflict and crisis situations (2)" "appropriate conflict resolution strategies (1)" "conflict situations (1)" "effective conflict resolution strategies (1)" |
| body | 10 | "body autonomy (2)" "body awareness (1)" "body perception (1)" "body systems (1)" "body temperature (1)" "the body (1)" "the human body systems (1)" "their body functions (1)" "various interrelated body systems (1)" |
| tooth | 10 | "your teeth (4)" "incisors/ erupting teeth/milk teeth (2)" "what teeth (2)" "brushing teeth (1)" "healthy teeth (1)" |
| factor | 10 | "factors (5)" "analyse factors (2)" "multiple factors (2)" "an influencing factor (1)" |
| goal | 10 | "common goals (2)" "goals (2)" "personal health goals (2)" "personal study goals (2)" "goal (1)" "health goals (1)" |
| responsibility | 10 | "personal responsibility (6)" "responsibilities (2)" "their responsibility (2)" |
| calorie | 10 | "calories (4)" "2,500 calories (3)" "how many calories (3)" |
| impact | 10 | "the impact (6)" "the possible impact (2)" "the media impact (1)" "the media's impact (1)" |
| assessment | 10 | "assessment (5)" "assessment criteria (3)" "self-assessment (2)" |
| instruction | 10 | "instruction (7)" "objective instruction (2)" "instructions (1)" |
| being | 10 | "well-being (7)" "well being (3)" |
| source | 10 | "different sources (3)" "information sources (2)" "some reliable sources (2)" "a credible source (1)" "sources (1)" "valid sources (1)" |
| technology | 10 | "interactive technology (7)" "technology (3)" |
| thought | 9 | "your thoughts (7)" "their thoughts (2)" |
| promotion | 9 | "health promotion (8)" "promotion (1)" |
| game | 9 | "games (2)" "the game (2)" "a relay game (1)" "another so-called energy game (1)" "played perceptual games (1)" "role-playing games (1)" "several active games (1)" |
| consequence | 9 | "the consequences (4)" "these consequences (2)" "a) distinguish short- and long-term consequences (1)" "the health-related consequences (1)" "what consequences (1)" |
| risk | 9 | "risk (3)" "risk taking behaviors (2)" "risks (2)" "health risks (1)" "the risk (1)" |
| agency | 9 | "at least 3 agencies (2)" "government agencies (2)" "regional health agencies (2)" "the assigned agencies (2)" "a health agency (1)" |
| education | 9 | "the 4 health education settings (4)" "medication education (3)" "sex education (2)" |
| study | 9 | "environmental studies (2)" "personal study goals (2)" "small study units (2)" "further studies (1)" "future studies (1)" "such studies (1)" |
| snack | 8 | "a snack (3)" "snacks (3)" "cariogenic snacks (1)" "your snack choices (1)" |
| digit | 8 | "1-digit number (2)" "3 age 8-9, multiply 2-digit number (2)" "a 1- digit number (2)" "a 4-digit number (2)" |
| number | 8 | "1-digit number (2)" "3 age 8-9, multiply 2-digit number (2)" "a 1- digit number (2)" "a 4-digit number (2)" |
| medication | 8 | "medication education (3)" "medication-taking skills (2)" "his medication (1)" "medication (1)" "some medication (1)" |
| emotion | 8 | "emotions (4)" "their emotions (2)" "various emotions (2)" |
| effect | 8 | "the cause/effect (2)" "the effects (2)" "effect (1)" "effects (1)" "the nutritive effect (1)" "what effects (1)" |
| habit | 8 | "health habits (4)" "everyday health habits (2)" "habits (2)" |
| surrounding | 8 | "their surroundings (6)" "one's immediate surroundings (1)" "the surroundings natural environment (1)" |
| change | 8 | "these changes (2)" "which changes (2)" "change (1)" "changes (1)" "physical, mental, emotional, and social changes (1)" "the changes (1)" |
| amount | 7 | "the amount (4)" "different amounts (2)" "any amount (1)" |
| HP | 7 | "hp (7)" |
| nutrition | 7 | "healthy nutrition (3)" "nutrition (2)" "a nutrition (1)" "nutrition labels (1)" |
| control | 7 | "control (2)" "greater control (2)" "self-control (2)" "some control (1)" |
| process | 7 | "the process (4)" "b) process health information (1)" "participatory dialogue process (1)" "the item development process (1)" |
| label | 7 | "this label (4)" "label (2)" "nutrition labels (1)" |
| need | 7 | "needs (4)" "individual needs (2)" "the need (1)" |
| fruit | 7 | "fruits (3)" "different fruits (2)" "fruit (1)" "sweets/ bread/fruits (1)" |

## Construct HL Competences definition:

### Accessing

| **Concept** | **Count** | **Noun Phrases** |
| --- | --- | --- |
| **health** | **31** | **"health (8)" "health information (7)" "health-related information (3)" "health products (2)" "health services (2)" "accurate health information (1)" "health issues (1)" "health resources (1)" "personal health (1)" "personal health decisions (1)" "regional health resources (1)" "the 4 health education settings (1)" "their health (1)" "valid health information (1)"** |
| **information** | **30** | **"information (10)" "health information (7)" "health-related information (3)" "accurate health information (1)" "consumer information (1)" "extract information (1)" "good information (1)" "information sources (1)" "reliable information (1)" "the information (1)" "valid health information (1)" "what information (1)" "which information (1)"** |
| **service** | **12** | **"services (8)" "health services (2)" "professional services (1)" "service navigation (1)"** |
| **product** | **8** | **"products (6)" "health products (2)"** |
| **student** | **7** | **"students (6)" "the student (1)"** |
| **source** | **5** | **"different sources (2)" "information sources (1)" "some reliable sources (1)" "valid sources (1)"** |
| **food** | **5** | **"which food (3)" "food choices (1)" "food options (1)"** |
| **ability** | **4** | **"ability (3)" "the ability (1)"** |
| **cold** | **4** | **"a cold (4)"** |
| **state** | **3** | **"state (2)" "the local and state levels (1)"** |
| **pupil** | **3** | **"pupils (3)"** |
| **resource** | **3** | **"electronic resources (1)" "health resources (1)" "regional health resources (1)"** |
| **level** | **2** | **"regional levels (1)" "the local and state levels (1)"** |
| **example** | **2** | **"an example (1)" "any other examples (1)"** |
| **access** | **2** | **"access (2)"** |
| **option** | **2** | **"food options (1)" "options (1)"** |
| **internet** | **2** | **"internet (1)" "the internet (1)"** |
| **education** | **1** | **"the 4 health education settings (1)"** |
| **appropriateness** | **1** | **"appropriateness (1)"** |
| **thinking** | **1** | **"critical thinking grade (1)"** |
| **grade** | **1** | **"critical thinking grade (1)"** |
| **instruction** | **1** | **"instruction (1)"** |
| **view** | **1** | **"different views (1)"** |
| **interpreting** | **1** | **"interpreting (1)"** |
| **viewpoint** | **1** | **"viewpoints (1)"** |
| **relationship** | **1** | **"causal relationships (1)"** |
| **conclusion** | **1** | **"conclusions (1)"** |
| **result** | **1** | **"results (1)"** |
| **assessment** | **1** | **"assessment (1)"** |
| **impact** | **1** | **"the impact (1)"** |
| **culture** | **1** | **"culture (1)"** |
| **medium** | **1** | **"media (1)"** |
| **technology** | **1** | **"technology (1)"** |
| **decision** | **1** | **"personal health decisions (1)"** |
| **practice** | **1** | **"practices (1)"** |
| **benefit** | **1** | **"the benefits (1)"** |
| **type** | **1** | **"various types (1)"** |
| **accuracy** | **1** | **"accuracy (1)"** |
| **quality** | **1** | **"quality (1)"** |
| **effectiveness** | **1** | **"the effectiveness (1)"** |
| **availability** | **1** | **"the availability (1)"** |
| **situation** | **1** | **"situations (1)"** |
| **way** | **1** | **"ways (1)"** |
| **parent** | **1** | **"your parents (1)"** |
| **friend** | **1** | **"friends (1)"** |
| **nutrition** | **1** | **"healthy nutrition (1)"** |
| **navigation** | **1** | **"service navigation (1)"** |
| **being** | **1** | **"well-being (1)"** |
| **empowerment** | **1** | **"empowerment (1)"** |
| **feeling** | **1** | **"enhanced feelings (1)"** |
| **use** | **1** | **"informed use (1)"** |
| **newspaper** | **1** | **"magazines/newspapers (1)"** |
| **choice** | **1** | **"food choices (1)"** |
| **magazine** | **1** | **"magazines/newspapers (1)"** |
| **book** | **1** | **"books (1)"** |
| **throat** | **1** | **"a cold, sore throat (1)"** |
| **hc** | **1** | **"hc (1)"** |
| **winter** | **1** | **"winter (1)"** |
| **dp** | **1** | **"dp (1)"** |
| **HP** | **1** | **"hp (1)"** |
| **aid** | **1** | **"aids (1)"** |
| **skill** | **1** | **"skills (1)"** |
| **people** | **1** | **"different people (1)"** |
| **place** | **1** | **"places (1)"** |
| **attention** | **1** | **"attention (1)"** |
| **meaning** | **1** | **"derive meaning (1)"** |
| **form** | **1** | **"different forms (1)"** |
| **communication** | **1** | **"communication (1)"** |
| **technique** | **1** | **"techniques (1)"** |
| **time** | **1** | **"times (1)"** |
| **consumer** | **1** | **"consumer information (1)"** |
| **channel** | **1** | **"diverse channels (1)"** |
| **t** | **1** | **"t (1)"** |
| **neighbourhood** | **1** | **"neighbourhood (1)"** |
| **indicator** | **1** | **"indicator (1)"** |
| **opinion** | **1** | **"opinions (1)"** |
| **issue** | **1** | **"health issues (1)"** |
| **question** | **1** | **"an important question (1)"** |
| **knowledge** | **1** | **"knowledge (1)"** |
| **setting** | **1** | **"the 4 health education settings (1)"** |
| **acces** | **1** | **"acces (1)"** |

### Understanding

| **Concept** | **Count** | **Noun Phrases** |
| --- | --- | --- |
| health | 84 | "health (20)" "the health (7)" "your health (6)" "health promotion (3)" "health care behaviors (2)" "health information (2)" "our health (2)" "their health (2)" "b) process health information (1)" "basic health knowledge (1)" "community health advocacy activities (1)" "community health plans (1)" "dental health literacy (1)" "everyday health habits (1)" "good health (1)" "good health care (1)" "health care (1)" "health concepts (1)" "health health mapping (1)" "health knowledge (1)" "health literacy skills (1)" "health risks (1)" "health skills (1)" "health-enhancing behaviors (1)" "health-literate individuals (1)" "health-promoting foods (1)" "health-related actions (1)" "health-related information (1)" "health-related life situations (1)" "lifelong health (1)" "life-long health (1)" "major health issues (1)" "my health examination report (1)" "non-health-promoting foods (1)" "personal and family health (1)" "personal health decisions (1)" "poor eyesight and good oral health (1)" "positive health practices (1)" "significant community health issues (1)" "the health information (1)" "the health topics (1)" "the health-related consequences (1)" "the term health (1)" "their sexual health (1)" "various community health issues (1)" "various health plans/strategies (1)" "what health information (1)" "your health relate (1)" |
| development | 17 | "development (10)" "sustainable development (2)" "growth development (1)" "individual development (1)" "mental and emotional development (1)" "the development (1)" "the expected development (1)" |
| food | 17 | "foods (2)" "the foods (2)" "all healthy foods (1)" "different food groups (1)" "food (1)" "food shopping (1)" "food-impulses (1)" "health-promoting foods (1)" "healthy food (1)" "non-health-promoting foods (1)" "the “food pyramid (1)" "the related foods (1)" "the two food categories (1)" "their regulary consumed food (1)" "various foods (1)" |
| skill | 15 | "skills (4)" "skill (3)" "medication-taking skills (2)" "effective interpersonal communication skills (1)" "environmental protection skills (1)" "first-aid skills (1)" "health literacy skills (1)" "health skills (1)" "proper rejection skills (1)" |
| student | 14 | "students (13)" "the student (1)" |
| other | 14 | "others (13)" "us/others (1)" |
| child | 13 | "the children (6)" "children (5)" "child (1)" "each child (1)" |
| community | 12 | "community (5)" "communities (2)" "community health advocacy activities (1)" "community health plans (1)" "community-based efforts (1)" "significant community health issues (1)" "various community health issues (1)" |
| school | 12 | "school (4)" "school environment (2)" "action-oriented school project (1)" "middle school (1)" "school map (1)" "schools (1)" "the school (1)" "the school environment (1)" |
| doctor | 12 | "the doctor (5)" "your doctor (4)" "a doctor (2)" "doctors (1)" |
| growth | 11 | "growth (8)" "growth development (1)" "individual growth (1)" "physical, mental,emotional, and social growth (1)" |
| disease | 11 | "disease (4)" "disease prevention (3)" "disease prevention concepts (1)" "diseases (1)" "infectious disease (1)" "life-threatening diseases (1)" |
| action | 10 | "the action (2)" "action (1)" "action-oriented school project (1)" "actions (1)" "ethically responsible actions (1)" "health-related actions (1)" "one's own actions (1)" "these actions (1)" "which action (1)" |
| family | 10 | "family (4)" "their families (2)" "families (1)" "family members (1)" "personal and family health (1)" "the family (1)" |
| information | 10 | "health information (2)" "information (2)" "b) process health information (1)" "consumer information (1)" "health-related information (1)" "the health information (1)" "this information (1)" "what health information (1)" |
| behavior | 9 | "behaviors (3)" "health care behaviors (2)" "healthcare behaviors (1)" "health-enhancing behaviors (1)" "personal behavior (1)" "risky and harmful behavior (1)" |
| life | 9 | "health-related life situations (1)" "life (1)" "life courses (1)" "life cycle (1)" "life-long health (1)" "life-threatening diseases (1)" "the social life (1)" "their lives (1)" "your everyday life (1)" |
| vaccination | 9 | "vaccination (3)" "vaccinations (3)" "oral vaccination/injection (1)" "the vaccination card (1)" "vaccination hl (1)" |
| hl | 9 | "functional hl (6)" "interactive hl (2)" "vaccination hl (1)" |
| influence | 8 | "the influence (7)" "what influences (1)" |
| change | 8 | "these changes (2)" "which changes (2)" "change (1)" "changes (1)" "physical, mental, emotional, and social changes (1)" "the changes (1)" |
| pupil | 8 | "pupils (7)" "pupils’ environmental awareness (1)" |
| environment | 7 | "school environment (2)" "the environment (2)" "the future?school environment (1)" "the school environment (1)" "the surroundings natural environment (1)" |
| ice | 7 | "this ice cream (3)" "ice cream (2)" "how much ice cream (1)" "this ice cream?skill (1)" |
| care | 7 | "care (3)" "health care behaviors (2)" "good health care (1)" "health care (1)" |
| grade | 6 | "grade (5)" "citizinship grade (1)" |
| tooth | 6 | "incisors/ erupting teeth/milk teeth (2)" "what teeth (2)" "your teeth (2)" |
| process | 6 | "the process (4)" "b) process health information (1)" "participatory dialogue process (1)" |
| understanding | 6 | "an understanding (2)" "understanding (2)" "this understanding (1)" "your understanding (1)" |
| cream | 6 | "this ice cream (3)" "ice cream (2)" "how much ice cream (1)" |
| pain | 5 | "chronic pain (1)" "pain (1)" "pain visit (1)" "the most frequent pain (1)" "their pain (1)" |
| alternative | 5 | "which alternatives (3)" "these alternatives (1)" "various alternatives (1)" |
| knowledge | 5 | "knowledge (2)" "basic health knowledge (1)" "health knowledge (1)" "the knowledge (1)" |
| medication | 5 | "medication-taking skills (2)" "medication (1)" "medication education (1)" "some medication (1)" |
| body | 5 | "body autonomy (2)" "body temperature (1)" "the body (1)" "their body functions (1)" |
| concept | 5 | "the concept (2)" "concepts (1)" "disease prevention concepts (1)" "health concepts (1)" |
| parent | 5 | "your parents (3)" "my parents (1)" "their parents (1)" |
| prevention | 5 | "disease prevention (3)" "correct prevention strategy (1)" "disease prevention concepts (1)" |
| way | 5 | "the ways (2)" "ways (2)" "the way (1)" |
| group | 5 | "a group (1)" "different food groups (1)" "each group (1)" "groups (1)" "peer group (1)" |
| individual | 5 | "health-literate individuals (2)" "individuals (2)" "all individuals (1)" |
| label | 5 | "this label (4)" "label (1)" |
| card | 4 | "playing cards (1)" "the cards (1)" "the playing cards (1)" "the vaccination card (1)" |
| interaction | 4 | "interpersonal interactions (4)" |
| mask | 4 | "a mask (1)" "his sanitary mask (1)" "my sanitary mask (1)" "sanitary masks (1)" |
| issue | 4 | "major health issues (1)" "significant community health issues (1)" "this issue (1)" "various community health issues (1)" |
| sex | 4 | "sex education (2)" "opposite-sex friendships (1)" "the opposite sex (1)" |
| digit | 4 | "1-digit number (1)" "3 age 8-9, multiply 2-digit number (1)" "a 1- digit number (1)" "a 4-digit number (1)" |
| number | 4 | "1-digit number (1)" "3 age 8-9, multiply 2-digit number (1)" "a 1- digit number (1)" "a 4-digit number (1)" |
| age | 4 | "3 age (1)" "3 age 8-9, multiply 2-digit number (1)" "4 age (1)" "age (1)" |
| fat | 4 | "saturated fat (3)" "fat (1)" |
| perspective | 4 | "personal and collective perspective (4)" |
| time | 4 | "time (2)" "enough time (1)" "the most important time (1)" |
| friendship | 4 | "friendship (1)" "harmonious friendships (1)" "my friendships (1)" "opposite-sex friendships (1)" |
| communication | 4 | "communication (2)" "effective interpersonal communication skills (1)" "various communication techniques (1)" |
| example | 4 | "examples (2)" "at least 3 examples (1)" "example (1)" |
| activity | 4 | "community health advocacy activities (1)" "physical activity (1)" "sexual activity (1)" "your daily activities (1)" |
| promotion | 4 | "health promotion (3)" "promotion (1)" |
| people | 4 | "people (2)" "all people (1)" "other people (1)" |
| scenario | 4 | "scenario (4)" |
| aspect | 4 | "aspects (2)" "the aspects (1)" "those aspects (1)" |
| practice | 4 | "practices (3)" "positive health practices (1)" |
| medicine | 4 | "your medicine (3)" "medicines (1)" |
| peer | 4 | "peer group (1)" "peer relationships (1)" "peers (1)" "their peers (1)" |
| rate | 3 | "higher pulse rates (1)" "the pulse rate (1)" "their pulse rate (1)" |
| self | 3 | "self (3)" |
| plan | 3 | "community health plans (1)" "how different plans (1)" "various health plans/strategies (1)" |
| resource | 3 | "a resource-based manner (1)" "resources (1)" "the resources (1)" |
| consequence | 3 | "the health-related consequences (1)" "these consequences (1)" "what consequences (1)" |
| snack | 3 | "a snack (1)" "cariogenic snacks (1)" "snacks (1)" |
| vegetable | 3 | "vegetables (3)" |
| fruit | 3 | "different fruits (1)" "fruits (1)" "sweets/ bread/fruits (1)" |
| drug | 3 | "drugs (1)" "illegal drugs (1)" "pharmaceutical drugs (1)" |
| decision | 3 | "our decision-making collective?is (1)" "personal health decisions (1)" "their individual decision-making (1)" |
| diet | 3 | "diet (2)" "your diet (1)" |
| calorie | 3 | "2,500 calories (1)" "calories (1)" "how many calories (1)" |
| education | 3 | "sex education (2)" "medication education (1)" |
| classmate | 3 | "classmates (1)" "my classmates (1)" "other classmates (1)" |
| puberty | 3 | "puberty (3)" |
| hygiene | 3 | "personal hygiene (3)" |
| peanut | 3 | "peanuts (2)" "peanut oil (1)" |
| sugar | 3 | "how much sugar (1)" "sugar (1)" "the word sugar (1)" |
| surrounding | 3 | "their surroundings (2)" "the surroundings natural environment (1)" |
| ability | 3 | "ability (3)" |
| relationship | 3 | "peer relationships (1)" "positive relationships (1)" "the relationship (1)" |
| role | 3 | "a positive, active role (1)" "the roles (1)" "tobacco-smoking?which role (1)" |
| being | 3 | "well-being (2)" "well being (1)" |
| respect | 3 | "respect (3)" |
| thought | 3 | "your thoughts (3)" |
| examination | 2 | "my health examination report (1)" "your physical examination (1)" |
| form | 2 | "different forms (2)" |
| meaning | 2 | "communicative hl derive meaning (1)" "derive meaning (1)" |
| word | 2 | "the word sugar (1)" "the words (1)" |
| connection | 2 | "the connection (2)" |
| sexuality | 2 | "the sexuality (1)" "their developing sexuality (1)" |
| member | 2 | "family members (1)" "members (1)" |
| Health | 2 | "health health mapping (1)" "health-literate individuals (1)" |
| effect | 2 | "the effects (1)" "the nutritive effect (1)" |
| HL | 2 | "functional hl (2)" |
| boy | 2 | "boys (1)" "boys’ trousers (1)" |

### Appraising

| **Concept** | **Count** | **Noun Phrases** |
| --- | --- | --- |
| health | 67 | "health (10)" "health information (6)" "health-related information (6)" "personal health decisions (3)" "your health (3)" "health care requirements (2)" "health habits (2)" "health practices (2)" "health products (2)" "personal and family health decisions (2)" "personal health goals (2)" "the health (2)" "a health agency (1)" "accurate health information (1)" "available health-related information (1)" "critically appraising health information (1)" "environmental health (1)" "health behaviors (1)" "health care (1)" "health communications (1)" "health literate (1)" "health pressures (1)" "health resources (1)" "health-related services (1)" "life-long health (1)" "one's health (1)" "personal health behaviors (1)" "regional health resources (1)" "suitable health products (1)" "the 4 health education settings (1)" "the health information (1)" "their health (1)" "their own health decisions (1)" "true and false health information (1)" "valid health information (1)" "various health issues (1)" "what specific health information (1)" |
| information | 33 | "health information (6)" "health-related information (6)" "information (5)" "accurate health information (1)" "available health-related information (1)" "contact information (1)" "critically appraising health information (1)" "extract information (1)" "information sources (1)" "information/data (1)" "reliable information (1)" "the health information (1)" "the information (1)" "the most wrong information (1)" "true and false health information (1)" "valid health information (1)" "what information (1)" "what specific health information (1)" "which information (1)" |
| service | 14 | "services (13)" "health-related services (1)" |
| product | 14 | "products (11)" "health products (2)" "suitable health products (1)" |
| student | 10 | "students (7)" "the student (3)" |
| practice | 9 | "practices (6)" "health practices (2)" "personal practices (1)" |
| behavior | 8 | "health behaviors (1)" "one's own behavior (1)" "personal behavior (1)" "personal health behaviors (1)" "risk taking behaviors (1)" "risky and harmful behaviors (1)" "their behaviors (1)" "your behavior (1)" |
| decision | 6 | "personal health decisions (3)" "personal and family health decisions (2)" "their own health decisions (1)" |
| pupil | 6 | "pupils (5)" "pupils’ ability (1)" |
| source | 5 | "a credible source (1)" "different sources (1)" "information sources (1)" "sources (1)" "valid sources (1)" |
| ability | 5 | "ability (3)" "pupils’ ability (1)" "the ability (1)" |
| way | 5 | "ways (4)" "various ways (1)" |
| life | 4 | "life (3)" "life-long health (1)" |
| impact | 4 | "the impact (2)" "the media impact (1)" "the media's impact (1)" |
| Pupils | 4 | "pupils (4)" |
| example | 4 | "examples (2)" "an example (1)" "any other examples (1)" |
| safety | 4 | "safety (4)" |
| medium | 4 | "media (1)" "the media (1)" "the media impact (1)" "the media's impact (1)" |
| grade | 4 | "grade (2)" "citizenship grade (1)" "critical thinking grade (1)" |
| state | 3 | "state (2)" "the local and state levels (1)" |
| child | 3 | "children (3)" |
| item | 3 | "developing items (1)" "items (1)" "the item development process (1)" |
| study | 3 | "further studies (1)" "future studies (1)" "such studies (1)" |
| reliability | 3 | "the reliability (3)" |
| consequence | 3 | "the consequences (2)" "these consequences (1)" |
| environment | 3 | "environment (1)" "the environment (1)" "the learning environment (1)" |
| community | 3 | "community (1)" "local communities (1)" "the community (1)" |
| disease | 3 | "disease (1)" "disease prevention (1)" "spread/prevent diseases (1)" |
| technology | 3 | "technology (3)" |
| prevention | 3 | "the prevention (2)" "disease prevention (1)" |
| family | 3 | "personal and family health decisions (2)" "family (1)" |
| result | 3 | "results (2)" "the results (1)" |
| lot | 3 | "a lot (3)" |
| risk | 3 | "risk (1)" "risk taking behaviors (1)" "risks (1)" |
| institution | 3 | "institutions (2)" "institution (1)" |
| organization | 3 | "organizations (2)" "organization (1)" |
| agency | 3 | "a health agency (1)" "at least 3 agencies (1)" "the assigned agencies (1)" |
| care | 3 | "health care requirements (2)" "health care (1)" |
| viewpoint | 2 | "different viewpoints (1)" "viewpoints (1)" |
| relationship | 2 | "causal relationships (2)" |
| conclusion | 2 | "conclusions (1)" "simple conclusions (1)" |
| culture | 2 | "culture (2)" |
| requirement | 2 | "health care requirements (2)" |
| policy | 2 | "policies (2)" |
| strategy | 2 | "strategies (2)" |
| factor | 2 | "factors (1)" "multiple factors (1)" |
| resource | 2 | "health resources (1)" "regional health resources (1)" |
| level | 2 | "regional levels (1)" "the local and state levels (1)" |
| habit | 2 | "health habits (2)" |
| area | 2 | "this action area (1)" "this area (1)" |
| development | 2 | "the development (1)" "the item development process (1)" |
| view | 2 | "different views (1)" "various views (1)" |
| goal | 2 | "personal health goals (2)" |
| instruction | 2 | "instruction (2)" |
| situation | 2 | "the situation (1)" "what everyday situations (1)" |
| game | 2 | "games (2)" |
| thinking | 2 | "critical thinking (1)" "critical thinking grade (1)" |
| use | 2 | "informed use (2)" |
| Health | 2 | "health (1)" "health-literate individuals (1)" |
| food | 2 | "fake food (1)" "food choices (1)" |
| choice | 2 | "choices (1)" "food choices (1)" |
| Judge | 2 | "hc judge (1)" "judge (1)" |
| doctor | 2 | "doctor (1)" "the doctor (1)" |
| datum | 2 | "a class computer data file (1)" "information/data (1)" |
| measure | 2 | "adjustment measures (1)" "measures (1)" |
| pressure | 2 | "health pressures (1)" "social pressures (1)" |
| effect | 2 | "effects (1)" "the cause/effect (1)" |
| management | 2 | "effective emotional management skills (1)" "emotional management methods (1)" |
| influence | 2 | "the influence (1)" "their influence (1)" |
| people | 2 | "different people (1)" "other people (1)" |
| truth | 1 | "the truth (1)" |
| order | 1 | "order (1)" |
| hc | 1 | "hc judge (1)" |
| behaviour | 1 | "your behaviour (1)" |
| exercise | 1 | "exercise (1)" |
| place | 1 | "places (1)" |
| HP | 1 | "hp (1)" |
| attention | 1 | "attention (1)" |
| time | 1 | "times (1)" |
| bias | 1 | "the bias (1)" |
| cold | 1 | "a cold (1)" |
| communication | 1 | "health communications (1)" |
| diet | 1 | "diet (1)" |
| consumer | 1 | "a selective consumer (1)" |
| summary | 1 | "a summary (1)" |
| individual | 1 | "health-literate individuals (1)" |
| file | 1 | "a class computer data file (1)" |
| computer | 1 | "a class computer data file (1)" |
| class | 1 | "a class computer data file (1)" |
| inclusion | 1 | "inclusion (1)" |
| reason | 1 | "at least 3 reasons (1)" |
| setting | 1 | "the 4 health education settings (1)" |
| education | 1 | "the 4 health education settings (1)" |
| contact | 1 | "contact information (1)" |
| accuracy | 1 | "accuracy (1)" |
| quality | 1 | "quality (1)" |
| principle | 1 | "principles (1)" |
| adjustment | 1 | "adjustment measures (1)" |
| literate | 1 | "health literate (1)" |
| method | 1 | "emotional management methods (1)" |

### Applying

| **Concept** | **Count** | **Noun Phrases** |
| --- | --- | --- |
| health | 58 | "health (5)" "their health (4)" "health information (3)" "lifelong health (3)" "personal health (3)" "the health (3)" "good health (2)" "health issues (2)" "personal health goals (2)" "personal health progress (2)" "that influence health and health behaviors (2)" "basic health knowledge (1)" "community and environmental health plans/strategies (1)" "community health advocacy activities (1)" "community health issues (1)" "different health plans (1)" "equitable health care (1)" "health advocacy (1)" "health advocacy strategies (1)" "health behaviours (1)" "health goals (1)" "health information project competition (1)" "health services (1)" "health-related actions (1)" "health-related decisions (1)" "health-related knowledge (1)" "mental and emotional health (1)" "our health (1)" "past health plans/strategies (1)" "personal health behaviors (1)" "positive health practices (1)" "the 4 health education settings (1)" "the health information (1)" "the health topics (1)" "the provided health information (1)" "their health literacy (1)" "their sexual health (1)" "your own health (1)" |
| student | 15 | "students (10)" "the student (4)" "a “fitness-pyramid” students (1)" |
| information | 11 | "health information (3)" "information (3)" "new information (2)" "health information project competition (1)" "the health information (1)" "the provided health information (1)" |
| situation | 11 | "potentially dangerous situations (3)" "situations (3)" "potentially harmful situations (2)" "behaviors/situations (1)" "different conflict and crisis situations (1)" "interactive situations (1)" |
| pupil | 11 | "pupils (9)" "pupils opportunities (1)" "pupils’ environmental awareness (1)" |
| way | 10 | "ways (4)" "the way (3)" "healthy ways (1)" "possible ways (1)" "the ways (1)" |
| community | 8 | "community (3)" "communities (1)" "community and environmental health plans/strategies (1)" "community health advocacy activities (1)" "community health issues (1)" "community-based efforts (1)" |
| skill | 8 | "communication skills (2)" "skills (2)" "effective interpersonal communication skills (1)" "interpersonal and decision-making skills (1)" "interpersonal communication skills (1)" "their emotional and interaction skills (1)" |
| action | 8 | "the action (2)" "action (1)" "actions (1)" "ethically responsible actions (1)" "health-related actions (1)" "these actions (1)" "which action (1)" |
| child | 7 | "the children (4)" "child abuse (1)" "other children (1)" "younger children (1)" |
| school | 7 | "school (4)" "middle school (1)" "schools (1)" "the school (1)" |
| family | 7 | "family (3)" "their families (2)" "families (1)" "family members (1)" |
| activity | 7 | "physical activity (5)" "community health advocacy activities (1)" "sexual activity (1)" |
| behavior | 6 | "behaviors (2)" "behaviors/situations (1)" "everyday behavior (1)" "personal health behaviors (1)" "that influence health and health behaviors (1)" |
| role | 6 | "the role (2)" "a positive, active role (1)" "different roles (1)" "role-playing games (1)" "the roles (1)" |
| other | 6 | "others (6)" |
| parent | 6 | "your parents (3)" "my parents (2)" "their parents (1)" |
| communication | 6 | "communication skills (2)" "appropriate communication techniques (1)" "effective communication (1)" "effective interpersonal communication skills (1)" "interpersonal communication skills (1)" |
| strategy | 6 | "strategies (2)" "community and environmental health plans/strategies (1)" "effective conflict resolution strategies (1)" "health advocacy strategies (1)" "past health plans/strategies (1)" |
| service | 5 | "services (3)" "health services (1)" "professional services (1)" |
| example | 5 | "example (2)" "an example (1)" "any other examples (1)" "at least 3 examples (1)" |
| grade | 5 | "grade (4)" "citizinship grade (1)" |
| change | 5 | "which changes (2)" "change (1)" "the changes (1)" "these changes (1)" |
| decision | 4 | "decisions (1)" "health-related decisions (1)" "interpersonal and decision-making skills (1)" "our decision-making collective?is (1)" |
| plan | 4 | "community and environmental health plans/strategies (1)" "different health plans (1)" "past health plans/strategies (1)" "various plans (1)" |
| food | 4 | "foods (1)" "healthy food (1)" "right balanced food (1)" "the two food categories (1)" |
| goal | 4 | "personal health goals (2)" "health goals (1)" "personal study goals (1)" |
| development | 4 | "sustainable development (2)" "development (1)" "the development (1)" |
| relationship | 4 | "healthy relationships (2)" "peer relationships (1)" "positive relationships (1)" |
| responsibility | 4 | "personal responsibility (4)" |
| safety | 4 | "road safety lessons (3)" "safety (1)" |
| barrier | 3 | "which barriers (2)" "barriers (1)" |
| issue | 3 | "health issues (2)" "community health issues (1)" |
| group | 3 | "a group (2)" "groups (1)" |
| life | 3 | "life events (2)" "daily life (1)" |
| diet | 3 | "a healthy diet (3)" |
| hand | 3 | "washing hands (2)" "your hands (1)" |
| lesson | 3 | "road safety lessons (3)" |
| ability | 3 | "ability (2)" "the ability (1)" |
| medicine | 3 | "your medicine (3)" |
| peer | 3 | "peer relationships (1)" "peers (1)" "their peers (1)" |
| choice | 3 | "choices (1)" "food choices (1)" "healthy choices (1)" |
| control | 3 | "greater control (2)" "some control (1)" |
| advocacy | 3 | "community health advocacy activities (1)" "health advocacy (1)" "health advocacy strategies (1)" |
| product | 3 | "products (3)" |
| agency | 3 | "the assigned agencies (2)" "at least 3 agencies (1)" |
| road | 3 | "road safety lessons (3)" |
| resource | 2 | "resources (1)" "the resources (1)" |
| NDICATOR | 2 | "indicator (2)" |
| importance | 2 | "the importance (2)" |
| treatment | 2 | "prescribed/recommended treatment (2)" |
| effect | 2 | "the effects (1)" "what effects (1)" |
| relaxation | 2 | "relaxation (1)" "relaxation exercises (1)" |
| need | 2 | "needs (1)" "the need (1)" |
| game | 2 | "role-playing games (1)" "several active games (1)" |
| washing | 2 | "washing hands (2)" |
| study | 2 | "environmental studies (1)" "personal study goals (1)" |
| progress | 2 | "personal health progress (2)" |
| conflict | 2 | "different conflict and crisis situations (1)" "effective conflict resolution strategies (1)" |
| knowledge | 2 | "basic health knowledge (1)" "health-related knowledge (1)" |
| care | 2 | "c) care (1)" "equitable health care (1)" |
| indicator | 2 | "indicator (2)" |
| organization | 2 | "organizations (2)" |
| institution | 2 | "institutions (2)" |
| emergency | 2 | "an emergency (1)" "emergencies (1)" |
| effort | 2 | "community-based efforts (1)" "persistent efforts (1)" |
| being | 2 | "well-being (2)" |
| instruction | 2 | "instructions (1)" "objective instruction (1)" |
| surrounding | 2 | "one's immediate surroundings (1)" "their surroundings (1)" |
| circumstance | 2 | "changing circumstances (2)" |
| event | 2 | "life events (2)" |
| sexuality | 2 | "the sexuality (1)" "their developing sexuality (1)" |
| risk | 1 | "the risk (1)" |
| living | 1 | "healthy living (1)" |
| Human Body | 1 | "human body (1)" |
| Food | 1 | "food choices (1)" |
| alcohol | 1 | "alcohol tobacco (1)" |
| member | 1 | "family members (1)" |
| drug | 1 | "other drugs (1)" |
| abuse | 1 | "child abuse (1)" |
| exploitation | 1 | "sexual exploitation (1)" |
| commitment | 1 | "a lifelong commitment (1)" |
| practice | 1 | "positive health practices (1)" |
| tobacco | 1 | "alcohol tobacco (1)" |
| paper | 1 | "a reflective paper (1)" |
| summary | 1 | "a summary (1)" |
| datum | 1 | "a class computer data file (1)" |
| computer | 1 | "a class computer data file (1)" |
| class | 1 | "a class computer data file (1)" |
| inclusion | 1 | "inclusion (1)" |
| report | 1 | "an oral report (1)" |
| setting | 1 | "the 4 health education settings (1)" |
| education | 1 | "the 4 health education settings (1)" |
| mail | 1 | "mail (1)" |
| e | 1 | "e (1)" |
| competition | 1 | "health information project competition (1)" |
| project | 1 | "health information project competition (1)" |
| friendship | 1 | "friendship (1)" |
| file | 1 | "a class computer data file (1)" |
| process | 1 | "the process (1)" |

## Construct HL Learning outcome definition

### Theoretical knowledge

| **Concept** | **Count** | **Noun Phrases** |
| --- | --- | --- |
| health | 64 | "health (13)" "health promotion (5)" "health information (3)" "personal health decisions (3)" "health-enhancing behaviors (2)" "personal and family health decisions (2)" "a health agency (1)" "adolescent health problems (1)" "age-appropriate health-related vocabulary (1)" "basic health knowledge (1)" "common childhood health problems (1)" "community and environmental health plans/strategies (1)" "community health issues (1)" "community health plans (1)" "environmental health (1)" "everyday health habits (1)" "good personal health (1)" "health behaviors (1)" "health care behaviors (1)" "health concepts (1)" "health literacy skills (1)" "health promoting products (1)" "health risks (1)" "health-related actions (1)" "health-related information (1)" "how social, emotional, and physical health (1)" "major health issues (1)" "personal and family health (1)" "personal health behaviors (1)" "personal health goals (1)" "professional health services (1)" "public health policies (1)" "significant community health issues (1)" "the 4 health education settings (1)" "their health (1)" "their health literacy (1)" "their own good health (1)" "their sexual health (1)" "valid health information (1)" "various community health issues (1)" "various health issues (1)" "what health information (1)" |
| student | 15 | "students (13)" "the student (2)" |
| development | 13 | "development (8)" "growth development (1)" "individual development (1)" "mental and emotional development (1)" "particularly development (1)" "the expected development (1)" |
| information | 10 | "health information (3)" "information (2)" "good information (1)" "health-related information (1)" "information/data (1)" "valid health information (1)" "what health information (1)" |
| life | 9 | "daily life (2)" "life (2)" "life courses (2)" "also people’s life courses (1)" "life course stages (1)" "life cycle (1)" |
| growth | 9 | "growth (7)" "growth development (1)" "individual growth (1)" |
| behavior | 9 | "health-enhancing behaviors (2)" "behaviors (1)" "health behaviors (1)" "health care behaviors (1)" "negative and positive behaviors (1)" "personal health behaviors (1)" "risk taking behaviors (1)" "risky and harmful behaviors (1)" |
| practice | 8 | "practices (7)" "b)examine practices (1)" |
| service | 8 | "services (7)" "professional health services (1)" |
| community | 7 | "community (1)" "community and environmental health plans/strategies (1)" "community health issues (1)" "community health plans (1)" "significant community health issues (1)" "the community (1)" "various community health issues (1)" |
| product | 7 | "products (6)" "health promoting products (1)" |
| disease | 6 | "disease prevention (3)" "disease (1)" "disease prevention concepts (1)" "infectious disease (1)" |
| pupil | 6 | "pupils (5)" "grade 4-6"to guide pupils (1)" |
| prevention | 5 | "disease prevention (3)" "correct prevention strategy (1)" "disease prevention concepts (1)" |
| promotion | 5 | "health promotion (5)" |
| decision | 5 | "personal health decisions (3)" "personal and family health decisions (2)" |
| issue | 5 | "community health issues (1)" "major health issues (1)" "significant community health issues (1)" "various community health issues (1)" "various health issues (1)" |
| impact | 4 | "the impact (2)" "the media impact (1)" "the media's impact (1)" |
| plan | 4 | "community and environmental health plans/strategies (1)" "community health plans (1)" "how different plans (1)" "various plans (1)" |
| strategy | 4 | "appropriate conflict resolution strategies (1)" "community and environmental health plans/strategies (1)" "correct prevention strategy (1)" "strategies (1)" |
| resource | 4 | "resources (2)" "a resource (1)" "a resource-based manner (1)" |
| concept | 4 | "concepts (1)" "disease prevention concepts (1)" "health concepts (1)" "the concept (1)" |
| grade | 4 | "grade (3)" "grade 4-6"to guide pupils (1)" |
| course | 4 | "life courses (2)" "also people’s life courses (1)" "life course stages (1)" |
| aspect | 4 | "aspects (3)" "the various aspects (1)" |
| mask | 4 | "a mask (1)" "his sanitary mask (1)" "my sanitary mask (1)" "sanitary masks (1)" |
| example | 4 | "examples (4)" |
| skill | 3 | "skills (2)" "health literacy skills (1)" |
| process | 3 | "the process (3)" |
| technology | 3 | "technology (3)" |
| medium | 3 | "media (1)" "the media impact (1)" "the media's impact (1)" |
| family | 3 | "personal and family health decisions (2)" "personal and family health (1)" |
| way | 3 | "ways (3)" |
| characteristic | 3 | "key characteristics (1)" "the characteristics (1)" "the secondary sexual characteristics (1)" |
| knowledge | 3 | "theoretical knowledge (2)" "basic health knowledge (1)" |
| influence | 3 | "the influence (3)" |
| problem | 3 | "adolescent common problems (1)" "adolescent health problems (1)" "common childhood health problems (1)" |
| agency | 2 | "a health agency (1)" "government agencies (1)" |
| time | 2 | "enough time (1)" "the most important time (1)" |
| sexuality | 2 | "the sexuality (1)" "their developing sexuality (1)" |
| ability | 2 | "ability (1)" "the ability (1)" |
| effect | 2 | "the cause/effect (1)" "the nutritive effect (1)" |
| advance | 2 | "medical advances (2)" |
| cause | 2 | "causes (1)" "the cause/effect (1)" |
| conflict | 2 | "appropriate conflict resolution strategies (1)" "conflict situations (1)" |
| situation | 2 | "conflict situations (1)" "various personal situations (1)" |
| childhood | 2 | "childhood (1)" "common childhood health problems (1)" |
| culture | 2 | "culture (2)" |
| body | 2 | "body systems (1)" "the human body systems (1)" |
| system | 2 | "body systems (1)" "the human body systems (1)" |
| school | 2 | "middle school (1)" "school (1)" |
| hl | 2 | "functional hl (2)" |
| stage | 2 | "different stages (1)" "life course stages (1)" |
| importance | 2 | "the importance (2)" |
| understanding | 2 | "an understanding (1)" "your understanding (1)" |
| puberty | 2 | "puberty (2)" |
| interaction | 2 | "the interaction (2)" |
| risk | 2 | "health risks (1)" "risk taking behaviors (1)" |
| literacy | 2 | "health literacy skills (1)" "their health literacy (1)" |
| belief | 1 | "cultural beliefs (1)" |
| benefit | 1 | "the benefits (1)" |
| significance | 1 | "the significance (1)" |
| structure | 1 | "the basic structure (1)" |
| function | 1 | "functions (1)" |
| examine | 1 | "b)examine practices (1)" |
| BENCHMARKS | 1 | "benchmarks (1)" |
| NDICATOR | 1 | "indicator (1)" |
| treatment | 1 | "prescribed/recommended treatment (1)" |
| research | 1 | "medical research (1)" |
| government | 1 | "government agencies (1)" |
| policy | 1 | "public health policies (1)" |
| adolescence | 1 | "adolescence (1)" |
| wellness | 1 | "wellness (1)" |
| term | 1 | "a) distinguish short- and long-term consequences (1)" |
| consequence | 1 | "a) distinguish short- and long-term consequences (1)" |
| pressure | 1 | "social pressures (1)" |
| goal | 1 | "personal health goals (1)" |
| Pupils | 1 | "pupils (1)" |
| teenager | 1 | "teenagers (1)" |
| criterion | 1 | "assessment criteria (1)" |
| participation | 1 | "participation (1)" |
| scenario | 1 | "scenario (1)" |
| Bill | 1 | "bill (1)" |
| today | 1 | "today (1)" |
| teacher | 1 | "the teacher (1)" |
| Bill ’s | 1 | "bill’s nasal allergy (1)" |
| allergy | 1 | "bill’s nasal allergy (1)" |
| doctor | 1 | "a doctor (1)" |
| variation | 1 | "individual variations (1)" |
| cold | 1 | "a cold (1)" |
| instruction | 1 | "instruction (1)" |
| guide | 1 | "grade 4-6"to guide pupils (1)" |
| habit | 1 | "everyday health habits (1)" |
| people | 1 | "also, people’s life courses (1)" |
| child | 1 | "children (1)" |
| nature | 1 | "the broad nature (1)" |
| manner | 1 | "a resource-based manner (1)" |
| assessment | 1 | "assessment criteria (1)" |
| lifestyle | 1 | "lifestyle (1)" |
| bridge | 1 | "bridges (1)" |

### Practical knowledge

| **Concept** | **Count** | **Noun Phrases** |
| --- | --- | --- |
| health | 68 | "health information (10)" "health (4)" "good health (3)" "lifelong health (3)" "the 4 health education settings (3)" "the health (3)" "valid health information (3)" "health-enhancing behaviors (2)" "life-long health (2)" "personal health (2)" "personal health goals (2)" "personal health progress (2)" "their health (2)" "community health (1)" "community health issues (1)" "different health plans (1)" "equitable health care (1)" "good health care (1)" "health advocacy strategies (1)" "health care behaviors (1)" "health goals (1)" "health information project competition (1)" "health issues (1)" "health products (1)" "health promoting products (1)" "health risks (1)" "health services (1)" "health-promoting foods (1)" "health-related decision-making (1)" "health-related decisions (1)" "health-related information (1)" "mental and emotional health (1)" "non-health-promoting foods (1)" "one's health (1)" "past health plans/strategies (1)" "personal health decisions (1)" "positive health practices (1)" "regional health resources (1)" "the provided health information (1)" "various health plans/strategies (1)" |
| student | 23 | "students (18)" "the student (5)" |
| information | 19 | "health information (10)" "valid health information (3)" "consumer information (1)" "contact information (1)" "health information project competition (1)" "health-related information (1)" "information (1)" "the provided health information (1)" |
| behavior | 16 | "behaviors (6)" "health-enhancing behaviors (2)" "age-appropriate behaviors (1)" "behaviors/situations (1)" "health care behaviors (1)" "healthcare behaviors (1)" "positive, personal hygienic behaviors (1)" "risk taking behaviors (1)" "risky and harmful behavior (1)" "risky and harmful behaviors (1)" |
| situation | 10 | "potentially dangerous situations (3)" "potentially harmful situations (2)" "behaviors/situations (1)" "different conflict and crisis situations (1)" "everyday situations (1)" "interactive situations (1)" "the situation (1)" |
| service | 9 | "services (8)" "health services (1)" |
| role | 8 | "the role (3)" "different roles (2)" "a positive, active role (1)" "role-playing games (1)" "the roles (1)" |
| product | 8 | "products (6)" "health products (1)" "health promoting products (1)" |
| child | 8 | "the children (5)" "child abuse (1)" "each child (1)" "other children (1)" |
| community | 7 | "communities (2)" "community (2)" "community health (1)" "community health issues (1)" "community-based efforts (1)" |
| strategy | 7 | "strategies (2)" "effective conflict resolution strategies (1)" "health advocacy strategies (1)" "healthy communication and resolution strategies (1)" "past health plans/strategies (1)" "various health plans/strategies (1)" |
| family | 7 | "family (3)" "families (2)" "family members (1)" "their families (1)" |
| skill | 7 | "skills (4)" "communication skills (1)" "effective communication skills (1)" "their emotional and interaction skills (1)" |
| other | 7 | "others (7)" |
| way | 7 | "ways (5)" "healthy ways (1)" "the ways (1)" |
| pupil | 6 | "pupils (5)" "pupils opportunities (1)" |
| school | 6 | "school (3)" "schools (2)" "the school (1)" |
| conflict | 5 | "conflict (2)" "conflicts (1)" "different conflict and crisis situations (1)" "effective conflict resolution strategies (1)" |
| example | 5 | "example (2)" "examples (2)" "at least 2 examples (1)" |
| practice | 5 | "practices (3)" "personal practices (1)" "positive health practices (1)" |
| risk | 5 | "risk (2)" "health risks (1)" "risk taking behaviors (1)" "the risk (1)" |
| responsibility | 5 | "personal responsibility (5)" |
| ability | 5 | "the ability (3)" "ability (2)" |
| food | 5 | "the foods (2)" "health-promoting foods (1)" "non-health-promoting foods (1)" "various foods (1)" |
| communication | 4 | "communication skills (1)" "effective communication (1)" "effective communication skills (1)" "healthy communication and resolution strategies (1)" |
| game | 4 | "a relay game (1)" "another so-called energy game (1)" "played perceptual games (1)" "role-playing games (1)" |
| agency | 4 | "at least 3 agencies (2)" "the assigned agencies (2)" |
| plan | 4 | "different health plans (1)" "past health plans/strategies (1)" "various health plans/strategies (1)" "various plans (1)" |
| choice | 4 | "healthy choices (2)" "food choices (1)" "personal choices (1)" |
| group | 4 | "a group (2)" "each group (1)" "their groups (1)" |
| emotion | 4 | "emotions (2)" "their emotions (1)" "various emotions (1)" |
| grade | 4 | "grade (4)" |
| decision | 4 | "health-related decision-making (1)" "health-related decisions (1)" "our final decision (1)" "personal health decisions (1)" |
| disease | 3 | "disease (2)" "disease prevention (1)" |
| card | 3 | "playing cards (1)" "the cards (1)" "the playing cards (1)" |
| activity | 3 | "physical activity (3)" |
| institution | 3 | "institutions (3)" |
| organization | 3 | "organizations (3)" |
| setting | 3 | "the 4 health education settings (3)" |
| care | 3 | "equitable health care (1)" "good health care (1)" "health care behaviors (1)" |
| goal | 3 | "personal health goals (2)" "health goals (1)" |
| impact | 3 | "the impact (2)" "the possible impact (1)" |
| behaviour | 3 | "behaviour (2)" "polite behaviour (1)" |
| treatment | 3 | "prescribed/recommended treatment (1)" "treatment (1)" "treatment options (1)" |
| education | 3 | "the 4 health education settings (3)" |
| safety | 2 | "outdoor safety tips (1)" "safety (1)" |
| aid | 2 | "aids (1)" "first aid (1)" |
| relationship | 2 | "peer relationships (1)" "positive relationships (1)" |
| peer | 2 | "peer relationships (1)" "their peers (1)" |
| hl | 2 | "critical hl (1)" "functional hl (1)" |
| question | 2 | "an important question (1)" "the questions (1)" |
| resolution | 2 | "effective conflict resolution strategies (1)" "healthy communication and resolution strategies (1)" |
| body | 2 | "body temperature (1)" "the body (1)" |
| reflection | 2 | "a reflection (1)" "the subsequent reflection (1)" |
| change | 2 | "changes (1)" "these changes (1)" |
| playing | 2 | "playing cards (1)" "the playing cards (1)" |
| relaxation | 2 | "relaxation (1)" "relaxation exercises (1)" |
| rate | 2 | "higher pulse rates (1)" "their pulse rate (1)" |
| issue | 2 | "community health issues (1)" "health issues (1)" |
| benefit | 2 | "the benefits (2)" |
| progress | 2 | "personal health progress (2)" |
| need | 2 | "needs (1)" "the need (1)" |
| option | 2 | "options (1)" "treatment options (1)" |
| crisis | 2 | "crises (1)" "different conflict and crisis situations (1)" |
| instruction | 2 | "instruction (1)" "instructions (1)" |
| NDICATOR | 2 | "indicator (2)" |
| state | 2 | "state (2)" |
| interaction | 2 | "the interaction (1)" "their emotional and interaction skills (1)" |
| effect | 2 | "the effects (1)" "what effects (1)" |
| pressure | 2 | "social pressure (1)" "social pressures (1)" |
| life | 2 | "life-long health (2)" |
| term | 2 | "a) distinguish short- and long-term consequences (1)" "terms (1)" |
| resource | 2 | "electronic resources (1)" "regional health resources (1)" |
| being | 2 | "well being (1)" "well-being (1)" |
| level | 1 | "regional levels (1)" |
| wellness | 1 | "wellness (1)" |
| consequence | 1 | "a) distinguish short- and long-term consequences (1)" |
| participation | 1 | "participation (1)" |
| improvement | 1 | "improvement (1)" |
| adjustment | 1 | "adjustments (1)" |
| basis | 1 | "a regular basis (1)" |
| making | 1 | "health-related decision-making (1)" |
| availability | 1 | "the availability (1)" |
| age | 1 | "age-appropriate behaviors (1)" |
| indicator | 1 | "indicator (1)" |
| effectiveness | 1 | "the effectiveness (1)" |
| Pupils | 1 | "pupils (1)" |
| medium | 1 | "media (1)" |
| culture | 1 | "culture (1)" |
| variety | 1 | "a variety (1)" |
| stress | 1 | "stress (1)" |
| solution | 1 | "solutions (1)" |
| regulation | 1 | "regulation (1)" |
| criterion | 1 | "assessment criteria (1)" |
| assessment | 1 | "assessment criteria (1)" |
| opportunity | 1 | "pupils opportunities (1)" |
| knowledge | 1 | "practical knowledge (1)" |
| lifestyle | 1 | "a healthy lifestyle (1)" |
| technology | 1 | "technology (1)" |
| feeling | 1 | "feelings (1)" |

### Critical Thinking

| **Concept** | **Count** | **Noun Phrases** |
| --- | --- | --- |
| health | 89 | "health (8)" "health information (8)" "health-related information (4)" "personal health decisions (3)" "community and environmental health issues (2)" "consumer health (2)" "health care requirements (2)" "health habits (2)" "health practices (2)" "health products (2)" "health promotion (2)" "health-related life situations (2)" "lifelong health (2)" "personal and family health decisions (2)" "personal health (2)" "personal health goals (2)" "public health policies (2)" "suitable health products (2)" "that influence health and health behaviors (2)" "the 4 health education settings (2)" "their health (2)" "valid health information (2)" "a health agency (1)" "adolescent health problems (1)" "community health advocacy activities (1)" "community health issues (1)" "different health plans (1)" "environmental health (1)" "good health (1)" "health behaviors (1)" "health care (1)" "health issues (1)" "health pressures (1)" "health promoting products (1)" "health resources (1)" "health services (1)" "health-promoting foods (1)" "life-long health (1)" "major health issues (1)" "non-health-promoting foods (1)" "one's health (1)" "personal and family health (1)" "personal health behaviors (1)" "personal health progress (1)" "professional health services (1)" "regional health resources (1)" "significant community health issues (1)" "the health (1)" "the provided health information (1)" "various community health issues (1)" "various health issues (1)" "your health (1)" |
| information | 30 | "health information (8)" "information (8)" "health-related information (4)" "valid health information (2)" "consumer information (1)" "contact information (1)" "information sources (1)" "information/data (1)" "reliable information (1)" "the most wrong information (1)" "the provided health information (1)" "valid and reliable information (1)" |
| student | 22 | "students (17)" "the student (5)" |
| service | 18 | "services (15)" "health services (1)" "professional health services (1)" "professional services (1)" |
| product | 17 | "products (10)" "health products (2)" "suitable health products (2)" "a yogurt product (1)" "health promoting products (1)" "this product (1)" |
| disease | 13 | "disease prevention (7)" "disease (3)" "common infectious diseases (1)" "infectious disease (1)" "spread/prevent diseases (1)" |
| situation | 11 | "potentially dangerous situations (3)" "health-related life situations (2)" "behaviors/situations (1)" "conflict situations (1)" "everyday situations (1)" "situations (1)" "the situation (1)" "various personal situations (1)" |
| behavior | 11 | "behaviors (3)" "behaviors/situations (1)" "health behaviors (1)" "negative and positive behaviors (1)" "personal health behaviors (1)" "risk taking behaviors (1)" "risky and harmful behaviors (1)" "that influence health and health behaviors (1)" "their behaviors (1)" |
| prevention | 10 | "disease prevention (7)" "the prevention (2)" "prevention (1)" |
| practice | 10 | "practices (6)" "health practices (2)" "b)examine practices (1)" "personal practices (1)" |
| scenario | 9 | "scenario (9)" |
| hl | 9 | "functional hl (4)" "critical hl (3)" "interactive hl (2)" |
| way | 9 | "ways (7)" "my way (1)" "various ways (1)" |
| community | 9 | "community and environmental health issues (2)" "community (1)" "community health advocacy activities (1)" "community health issues (1)" "local communities (1)" "significant community health issues (1)" "the community (1)" "various community health issues (1)" |
| child | 8 | "the children (5)" "child abuse (1)" "each child (1)" "other children (1)" |
| issue | 8 | "community and environmental health issues (2)" "community health issues (1)" "health issues (1)" "major health issues (1)" "significant community health issues (1)" "various community health issues (1)" "various health issues (1)" |
| life | 8 | "life (4)" "health-related life situations (2)" "life cycle (1)" "life-long health (1)" |
| food | 8 | "food (2)" "the foods (2)" "food choices (1)" "health-promoting foods (1)" "non-health-promoting foods (1)" "various foods (1)" |
| skill | 7 | "different coping skills (2)" "proper rejection skills (2)" "effective emotional management skills (1)" "first aid skills (1)" "medication-taking skills (1)" |
| medication | 6 | "medication education (2)" "his medication (1)" "medication (1)" "medication-taking skills (1)" "some medication (1)" |
| pupil | 6 | "pupils (5)" "pupils’ ability (1)" |
| source | 6 | "different sources (2)" "a credible source (1)" "information sources (1)" "some reliable sources (1)" "sources (1)" |
| choice | 6 | "diet choices (2)" "choices (1)" "food choices (1)" "personal choices (1)" "your snack choices (1)" |
| agency | 6 | "at least 3 agencies (2)" "government agencies (2)" "a health agency (1)" "the assigned agencies (1)" |
| strategy | 6 | "strategies (3)" "appropriate conflict resolution strategies (1)" "healthy communication and resolution strategies (1)" "your coping strategies (1)" |
| impact | 5 | "the impact (3)" "the media impact (1)" "the media's impact (1)" |
| school | 5 | "school (5)" |
| decision | 5 | "personal health decisions (3)" "personal and family health decisions (2)" |
| safety | 5 | "safety (4)" "safety tips (1)" |
| diet | 5 | "a healthy, balanced diet (2)" "diet choices (2)" "diet (1)" |
| influence | 5 | "the influence (2)" "their influence (2)" "that influence health and health behaviors (1)" |
| activity | 5 | "physical activity (2)" "community health advocacy activities (1)" "leisure activities (1)" "our leisure activities (1)" |
| Pupils | 5 | "pupils (5)" |
| policy | 4 | "policies (2)" "public health policies (2)" |
| education | 4 | "medication education (2)" "the 4 health education settings (2)" |
| thinking | 4 | "critical thinking (2)" "critical thinking grade (1)" "critical thinking/evaluation (1)" |
| grade | 4 | "grade (2)" "citizenship grade (1)" "critical thinking grade (1)" |
| example | 4 | "examples (2)" "at least 3 examples (1)" "example (1)" |
| ability | 4 | "ability (2)" "pupils’ ability (1)" "the ability (1)" |
| family | 4 | "personal and family health decisions (2)" "family (1)" "personal and family health (1)" |
| conflict | 4 | "conflict (2)" "appropriate conflict resolution strategies (1)" "conflict situations (1)" |
| development | 4 | "development (3)" "mental and emotional development (1)" |
| treatment | 3 | "correct medical treatment (2)" "treatment (1)" |
| institution | 3 | "institutions (2)" "institution (1)" |
| plan | 3 | "different health plans (1)" "how different plans (1)" "various plans (1)" |
| growth | 3 | "growth (3)" |
| organization | 3 | "organizations (2)" "organization (1)" |
| change | 3 | "changes (1)" "physical, mental, emotional, and social changes (1)" "these changes (1)" |
| game | 3 | "a relay game (1)" "another so-called energy game (1)" "role-playing games (1)" |
| toothpaste | 3 | "toothpaste (2)" "the toothpaste (1)" |
| parent | 3 | "my parents (2)" "parents (1)" |
| doctor | 3 | "the doctor (2)" "doctor (1)" |
| card | 3 | "playing cards (1)" "the cards (1)" "the playing cards (1)" |
| consumer | 3 | "consumer health (2)" "consumer information (1)" |
| risk | 3 | "risk (1)" "risk taking behaviors (1)" "the risk (1)" |
| body | 3 | "body systems (1)" "body temperature (1)" "the body (1)" |
| technology | 3 | "technology (3)" |
| state | 3 | "state (2)" "the local and state levels (1)" |
| thought | 3 | "your thoughts (3)" |
| result | 3 | "results (2)" "the results (1)" |
| reliability | 3 | "the reliability (3)" |
| factor | 3 | "factors (2)" "multiple factors (1)" |
| environment | 3 | "environment (1)" "the environment (1)" "the learning environment (1)" |
| responsibility | 3 | "personal responsibility (2)" "responsibilities (1)" |
| research | 3 | "medical research (3)" |
| care | 3 | "health care requirements (2)" "health care (1)" |
| medium | 3 | "media (1)" "the media impact (1)" "the media's impact (1)" |
| consequence | 3 | "the consequences (2)" "a) distinguish short- and long-term consequences (1)" |
| culture | 2 | "culture (2)" |
| problem | 2 | "adolescent health problems (1)" "problem (1)" |
| government | 2 | "government agencies (2)" |
| promotion | 2 | "health promotion (2)" |
| requirement | 2 | "health care requirements (2)" |
| goal | 2 | "personal health goals (2)" |
| indicator | 2 | "indicator (2)" |
| resource | 2 | "health resources (1)" "regional health resources (1)" |
| level | 2 | "regional levels (1)" "the local and state levels (1)" |
| pressure | 2 | "health pressures (1)" "social pressures (1)" |
| measure | 2 | "adjustment measures (1)" "measures (1)" |
| communication | 2 | "effective communication (1)" "healthy communication and resolution strategies (1)" |
| resolution | 2 | "appropriate conflict resolution strategies (1)" "healthy communication and resolution strategies (1)" |
| benefit | 2 | "the benefits (2)" |
| basis | 2 | "a regular basis (1)" "the basis (1)" |
| relationship | 2 | "causal relationships (2)" |
| habit | 2 | "health habits (2)" |
| criterion | 2 | "assessment criteria (2)" |
| instruction | 2 | "instruction (2)" |
| conclusion | 2 | "conclusions (1)" "simple conclusions (1)" |
| viewpoint | 2 | "different viewpoints (1)" "viewpoints (1)" |
| view | 2 | "different views (1)" "various views (1)" |
| nutrition | 2 | "nutrition (2)" |
| aid | 2 | "first aid (1)" "first aid skills (1)" |
| leisure | 2 | "leisure activities (1)" "our leisure activities (1)" |
| rejection | 2 | "proper rejection skills (2)" |
| condition | 2 | "my condition (1)" "your worsened condition (1)" |
| classmate | 2 | "my classmate (1)" "my classmates (1)" |
| John | 2 | "john (1)" "john’s kindness (1)" |
| cause | 2 | "causes (1)" "the cause/effect (1)" |
| question | 2 | "ethical questions (1)" "the questions (1)" |
| advance | 2 | "medical advances (2)" |

### Self-awareness

| **Concept** | **Count** | **Noun Phrases** |
| --- | --- | --- |
| health | 39 | "health (4)" "personal health (4)" "mental health (3)" "good health (2)" "health-related life situations (2)" "lifelong health (2)" "one's health (2)" "personal health progress (2)" "their health (2)" "community health (1)" "community health advocacy activities (1)" "different health plans (1)" "health advocacy (1)" "health issues (1)" "health knowledge (1)" "health promoting behaviours (1)" "health services (1)" "health-related decision-making (1)" "health-related decisions (1)" "life-long health (1)" "mental and emotional health (1)" "personal health behaviors (1)" "personal health goals (1)" "professional health services (1)" "their sexual health (1)" |
| self | 10 | "a healthy self-concept (2)" "self (2)" "a self-directed learner (1)" "self-assessment (1)" "self-awareness (1)" "self-confidence (1)" "self-control (1)" "self-worth (functional hl (1)" |
| pupil | 10 | "pupils (9)" "pupils opportunities (1)" |
| student | 10 | "students (9)" "the student (1)" |
| feeling | 7 | "feelings (3)" "enhanced feelings (1)" "positive feelings (1)" "the feeling (1)" "your feelings (1)" |
| grade | 7 | "grade (6)" "grade formulation (1)" |
| behavior | 6 | "behaviors (2)" "age-appropriate behaviors (1)" "one's own behavior (1)" "personal health behaviors (1)" "positive, personal hygienic behaviors (1)" |
| situation | 6 | "health-related life situations (2)" "different conflict and crisis situations (1)" "interactive situations (1)" "situations (1)" "various personal situations (1)" |
| skill | 6 | "proper rejection skills (2)" "effective communication skills (1)" "effective emotional management skills (1)" "interpersonal communication skills (1)" "their emotional and interaction skills (1)" |
| hl | 6 | "interactive hl (4)" "critical hl (1)" "self-worth (functional hl (1)" |
| interaction | 6 | "interpersonal interactions (4)" "social interaction (1)" "their emotional and interaction skills (1)" |
| other | 5 | "others (5)" |
| responsibility | 5 | "personal responsibility (4)" "their responsibility (1)" |
| way | 5 | "ways (3)" "healthy ways (1)" "the ways (1)" |
| ability | 4 | "ability (2)" "the ability (2)" |
| choice | 4 | "choices (1)" "food choices (1)" "one's own choices (1)" "personal choices (1)" |
| goal | 4 | "common goals (1)" "goals (1)" "personal health goals (1)" "personal study goals (1)" |
| sex | 4 | "sex education (2)" "opposite-sex friendships (1)" "the opposite sex (1)" |
| body | 4 | "body autonomy (2)" "body awareness (1)" "body perception (1)" |
| role | 4 | "the role (2)" "different roles (1)" "their own role (1)" |
| example | 3 | "at least 3 examples (1)" "example (1)" "examples (1)" |
| disease | 3 | "disease (2)" "disease prevention (1)" |
| activity | 3 | "community health advocacy activities (1)" "physical activity (1)" "sexual activity (1)" |
| service | 3 | "health services (1)" "professional health services (1)" "professional services (1)" |
| communication | 3 | "communication (1)" "effective communication skills (1)" "interpersonal communication skills (1)" |
| study | 3 | "environmental studies (1)" "personal study goals (1)" "small study units (1)" |
| being | 3 | "well-being (2)" "well being (1)" |
| information | 3 | "information (3)" |
| life | 3 | "health-related life situations (2)" "life-long health (1)" |
| group | 3 | "a group (1)" "the group (1)" "the group relay race (1)" |
| opinion | 3 | "your opinion (2)" "opinions (1)" |
| scenario | 3 | "scenario (3)" |
| concept | 3 | "a healthy self-concept (2)" "the concept (1)" |
| development | 3 | "development (3)" |
| growth | 3 | "growth (3)" |
| education | 3 | "sex education (2)" "medication education (1)" |
| friendship | 3 | "harmonious friendships (1)" "my friendships (1)" "opposite-sex friendships (1)" |
| response | 3 | "your response (3)" |
| indicator | 2 | "indicator (2)" |
| progress | 2 | "personal health progress (2)" |
| decision | 2 | "health-related decision-making (1)" "health-related decisions (1)" |
| family | 2 | "family (2)" |
| community | 2 | "community health (1)" "community health advocacy activities (1)" |
| technology | 2 | "interactive technology (2)" |
| sexuality | 2 | "the sexuality (1)" "their developing sexuality (1)" |
| story | 2 | "this story (2)" |
| exercise | 2 | "exercises (1)" "relaxation exercises (1)" |
| relaxation | 2 | "relaxation (1)" "relaxation exercises (1)" |
| basis | 2 | "a basis (1)" "a regular basis (1)" |
| advocacy | 2 | "community health advocacy activities (1)" "health advocacy (1)" |
| awareness | 2 | "body awareness (1)" "self-awareness (1)" |
| knowledge | 2 | "health knowledge (1)" "knowledge (1)" |
| school | 2 | "middle school (1)" "the school sports meet (1)" |
| autonomy | 2 | "body autonomy (2)" |
| rejection | 2 | "proper rejection skills (2)" |
| friend | 2 | "my friends (1)" "your friend’s request (1)" |
| need | 2 | "needs (2)" |
| party | 2 | "a party (1)" "our party (1)" |
| mother | 2 | "my mother (1)" "your mother’s worry (1)" |
| management | 2 | "effective emotional management skills (1)" "emotional management methods (1)" |
| stress | 2 | "stress (2)" |
| part | 2 | "a key part (1)" "part (1)" |
| classmate | 2 | "classmates (1)" "my classmates (1)" |
| child | 2 | "children (1)" "the children (1)" |
| bar | 1 | "a karaoke bar (1)" |
| karaoke | 1 | "a karaoke bar (1)" |
| happiness | 1 | "happiness (1)" |
| graduation | 1 | "graduation ceremony (1)" |
| kind | 1 | "this kind (1)" |
| ceremony | 1 | "graduation ceremony (1)" |
| place | 1 | "place (1)" |
| request | 1 | "your friend’s request (1)" |
| atmosphere | 1 | "the joyful atmosphere (1)" |
| alcohol | 1 | "alcohol (1)" |
| cigarette | 1 | "their cigarettes (1)" |
| beverage | 1 | "alcoholic beverages (1)" |
| today | 1 | "today (1)" |
| medication | 1 | "medication education (1)" |
| pride | 1 | "pride (1)" |
| worry | 1 | "your mother’s worry (1)" |
| method | 1 | "emotional management methods (1)" |
| meet | 1 | "the school sports meet (1)" |
| game | 1 | "the game (1)" |
| member | 1 | "members (1)" |
| fun | 1 | "fun (1)" |
| boy | 1 | "boys’ trousers (1)" |
| trouser | 1 | "boys’ trousers (1)" |
| girl | 1 | "girls’ bra straps (1)" |
| bra | 1 | "girls’ bra straps (1)" |
| strap | 1 | "girls’ bra straps (1)" |
| thought | 1 | "your thoughts (1)" |
| puberty | 1 | "puberty (1)" |
| HL | 1 | "functional hl (1)" |
| sport | 1 | "the school sports meet (1)" |
| athlete | 1 | "an athlete (1)" |
| relay | 1 | "the group relay race (1)" |
| race | 1 | "the group relay race (1)" |
| class | 1 | "our class (1)" |
| lot | 1 | "a lot (1)" |
| confidence | 1 | "self-confidence (1)" |

### Citizenship

| **Concept** | **Count** | **Noun Phrases** |
| --- | --- | --- |
| health | 45 | "health (6)" "the health (6)" "community and environmental health issues (2)" "health environment (2)" "health habits (2)" "mental health (2)" "regional health agencies (2)" "community and environmental health plans/strategies (1)" "community health (1)" "community health advocacy activities (1)" "community health issues (1)" "community health plans (1)" "environmental health (1)" "equitable health care (1)" "good health (1)" "good health care (1)" "health advocacy strategies (1)" "health literacy milestones (1)" "health promoting behaviours (1)" "health-related information (1)" "how appropriate and inappropriate health practices (1)" "major health issues (1)" "past health plans/strategies (1)" "personal and family health (1)" "positive health practices (1)" "significant community health issues (1)" "the health topics (1)" "various community health issues (1)" "various health issues (1)" "various health plans/strategies (1)" |
| community | 30 | "community (7)" "communities (4)" "community and environmental health issues (2)" "various community (2)" "a beautiful community (1)" "a community (1)" "a healthy community (1)" "community and environmental health plans/strategies (1)" "community health (1)" "community health advocacy activities (1)" "community health issues (1)" "community health plans (1)" "community-based efforts (1)" "local communities (1)" "our community (1)" "our community environment (1)" "significant community health issues (1)" "the community (1)" "various community health issues (1)" |
| family | 17 | "family (9)" "families (3)" "their families (3)" "family members (1)" "personal and family health (1)" |
| student | 13 | "students (12)" "the student (1)" |
| environment | 12 | "environment (2)" "health environment (2)" "the environment (2)" "the living environment (2)" "environments (1)" "our community environment (1)" "the learning environment (1)" "the surroundings natural environment (1)" |
| other | 10 | "others (10)" |
| peer | 9 | "peers (7)" "peer relationships (1)" "their peers (1)" |
| people | 7 | "other people (6)" "people (1)" |
| issue | 7 | "community and environmental health issues (2)" "community health issues (1)" "major health issues (1)" "significant community health issues (1)" "various community health issues (1)" "various health issues (1)" |
| plan | 6 | "community and environmental health plans/strategies (1)" "community health plans (1)" "how different plans (1)" "past health plans/strategies (1)" "various health plans/strategies (1)" "various plans (1)" |
| communication | 6 | "effective interpersonal communication skills (2)" "appropriate communication techniques (1)" "communication (1)" "communication skills (1)" "various communication techniques (1)" |
| way | 6 | "ways (4)" "the ways (1)" "various ways (1)" |
| information | 6 | "information (3)" "health-related information (1)" "information/data (1)" "valid and reliable information (1)" |
| relationship | 6 | "healthy relationships (2)" "relationships (2)" "peer relationships (1)" "positive relationships (1)" |
| role | 6 | "the role (3)" "a positive, active role (1)" "the roles (1)" "their own role (1)" |
| skill | 6 | "effective interpersonal communication skills (2)" "environmental protection skills (2)" "communication skills (1)" "interpersonal and decision-making skills (1)" |
| school | 6 | "schools (3)" "school (2)" "the school (1)" |
| pupil | 6 | "pupils (4)" "pupils’ ability (1)" "pupils’ environmental awareness (1)" |
| surrounding | 5 | "their surroundings (3)" "one's immediate surroundings (1)" "the surroundings natural environment (1)" |
| interaction | 5 | "interpersonal interactions (5)" |
| life | 5 | "life (3)" "their life (1)" "their lives (1)" |
| example | 5 | "examples (3)" "at least 3 examples (1)" "example (1)" |
| strategy | 5 | "community and environmental health plans/strategies (1)" "health advocacy strategies (1)" "past health plans/strategies (1)" "strategies (1)" "various health plans/strategies (1)" |
| factor | 4 | "factors (2)" "an influencing factor (1)" "multiple factors (1)" |
| Pupils | 4 | "pupils (4)" |
| self | 4 | "self (4)" |
| child | 4 | "the children (2)" "children (1)" "emerging child autonomy (1)" |
| hl | 4 | "interactive hl (2)" "critical hl (1)" "functional hl (1)" |
| behavior | 4 | "behaviors (1)" "individual behavior (1)" "individual behaviors (1)" "personal behavior (1)" |
| scenario | 4 | "scenario (4)" |
| ability | 4 | "ability (2)" "pupils’ ability (1)" "the ability (1)" |
| impact | 3 | "the impact (2)" "the possible impact (1)" |
| care | 3 | "care (1)" "equitable health care (1)" "good health care (1)" |
| choice | 3 | "healthy choices (2)" "choices (1)" |
| practice | 3 | "how appropriate and inappropriate health practices (1)" "positive health practices (1)" "support practices (1)" |
| development | 3 | "sustainable development (2)" "the development (1)" |
| thought | 3 | "your thoughts (2)" "their thoughts (1)" |
| protection | 3 | "environmental protection skills (2)" "environmental protection (1)" |
| reliability | 3 | "the reliability (3)" |
| agency | 2 | "regional health agencies (2)" |
| habit | 2 | "health habits (2)" |
| consequence | 2 | "the consequences (2)" |
| method | 2 | "different methods (1)" "methods (1)" |
| matter | 2 | "matters (2)" |
| organization | 2 | "organization (2)" |
| state | 2 | "state (2)" |
| service | 2 | "services (2)" |
| food | 2 | "foods (1)" "the two food categories (1)" |
| cause | 2 | "causes (1)" "the cause/effect (1)" |
| advance | 2 | "medical advances (2)" |
| resource | 2 | "resources (2)" |
| advocacy | 2 | "community health advocacy activities (1)" "health advocacy strategies (1)" |
| parent | 2 | "parents (1)" "their parents (1)" |
| al | 2 | "al (1)" "schwartz et al (1)" |
| criterion | 2 | "assessment criteria (2)" |
| technique | 2 | "appropriate communication techniques (1)" "various communication techniques (1)" |
| Assessment | 2 | "assessment criteria (2)" |
| autonomy | 2 | "body autonomy (1)" "emerging child autonomy (1)" |
| grade | 2 | "citizenship grade (1)" "citizinship grade (1)" |
| instruction | 2 | "instruction (1)" "objective instruction (1)" |
| action | 2 | "actions (1)" "one's own actions (1)" |
| friend | 2 | "a friend (1)" "my friend (1)" |
| race | 2 | "the cleaning race (1)" "the relay race (1)" |
| class | 2 | "our class (1)" "our class win second place (1)" |
| appreciation | 2 | "appreciation (2)" |
| encouragement | 2 | "encouragement (2)" |
| effort | 2 | "an effort (1)" "community-based efforts (1)" |
| response | 2 | "your response (2)" |
| living | 2 | "the living environment (2)" |
| friendship | 2 | "friendship (1)" "harmonious friendships (1)" |
| need | 2 | "needs (1)" "the need (1)" |
| support | 2 | "support (1)" "support practices (1)" |
| adult | 2 | "adults (2)" |
| influence | 2 | "the influence (2)" |
| responsibility | 2 | "responsibilities (1)" "their responsibility (1)" |
| year | 1 | "ten years (1)" |
| body | 1 | "body autonomy (1)" |
| feeling | 1 | "feelings (1)" |
| help | 1 | "help (1)" |
| awareness | 1 | "pupils’ environmental awareness (1)" |
| citizinship | 1 | "citizinship grade (1)" |
| acting | 1 | "acting (1)" |
| earth | 1 | "our earth (1)" |
| aim | 1 | "the aim (1)" |
| behaviour | 1 | "health promoting behaviours (1)" |
| cleaning | 1 | "the cleaning race (1)" |
| relay | 1 | "the relay race (1)" |
| Andy | 1 | "andy’s excellent performance (1)" |
| place | 1 | "our class win second place (1)" |
| win | 1 | "our class win second place (1)" |
| runner | 1 | "the last runner (1)" |
| sadness | 1 | "may’s sadness (1)" |
| May | 1 | "may’s sadness (1)" |
| game | 1 | "the game (1)" |
| meet | 1 | "the sports meet (1)" |
| sport | 1 | "the sports meet (1)" |
| day | 1 | "the day (1)" |
| importance | 1 | "the importance (1)" |
| performance | 1 | "andy’s excellent performance (1)" |
| world | 1 | "the world (1)" |

## HL levels and skills

### Functional HL skills

| **Concept** | **Count** | **Noun Phrases** |
| --- | --- | --- |
| ice | 11 | "ice cream (4)" "this ice cream (4)" "how much ice cream (3)" |
| cream | 11 | "ice cream (4)" "this ice cream (4)" "how much ice cream (3)" |
| calorie | 7 | "calories (3)" "2,500 calories (2)" "how many calories (2)" |
| fat | 7 | "saturated fat (6)" "fat (1)" |
| answer | 6 | "the only correct answer (3)" "answer (2)" "potential answer options (1)" |
| health | 6 | "health (1)" "health care (1)" "my health examination report (1)" "poor eyesight and good oral health (1)" "the 4 health education settings (1)" "the provided health information (1)" |
| label | 5 | "this label (4)" "label (1)" |
| grade | 4 | "grade (4)" |
| age | 4 | "2 age (1)" "3 age (1)" "3 age 8-9, multiply 2-digit number (1)" "4 age (1)" |
| number | 4 | "1-digit number (1)" "3 age 8-9, multiply 2-digit number (1)" "a 1- digit number (1)" "a 4-digit number (1)" |
| digit | 4 | "1-digit number (1)" "3 age 8-9, multiply 2-digit number (1)" "a 1- digit number (1)" "a 4-digit number (1)" |
| amount | 3 | "the amount (2)" "any amount (1)" |
| serving | 3 | "one serving (2)" "two servings (1)" |
| word | 3 | "the word sugar (1)" "the words (1)" "word recognition (1)" |
| gram | 3 | "how many grams (2)" "60 grams (1)" |
| g | 3 | "42 g (2)" "60 g (1)" |
| percentage | 3 | "what percentage (2)" "percentage (1)" |
| skill | 3 | "skill (3)" |
| container | 3 | "the entire container (2)" "half the container (1)" |
| diet | 2 | "your diet (2)" |
| carbohydrate | 2 | "carbohydrates (2)" |
| cup | 2 | "1 cup (1)" "up to 1 cup (1)" |
| day | 2 | "a day (2)" |
| value | 2 | "your daily value (2)" |
| snack | 2 | "a snack (2)" |
| doctor | 2 | "your doctor (2)" |
| examination | 2 | "my health examination report (1)" "your physical examination (1)" |
| peanut | 2 | "peanuts (2)" |
| sugar | 2 | "how much sugar (1)" "the word sugar (1)" |
| comprehension | 2 | "reading comprehension (2)" |
| numeracy | 2 | "numeracy (2)" |
| pronunciation | 1 | "pronunciation (1)" |
| mail | 1 | "mail (1)" |
| agency | 1 | "at least 3 agencies (1)" |
| education | 1 | "the 4 health education settings (1)" |
| setting | 1 | "the 4 health education settings (1)" |
| food | 1 | "food (1)" |
| math | 1 | "mental math (1)" |
| Locate | 1 | "locate (1)" |
| denominator | 1 | "same denominator (1)" |
| Add | 1 | "add fractions (1)" |
| information | 1 | "the provided health information (1)" |
| product | 1 | "products (1)" |
| Multiply | 1 | "3 age 8-9, multiply 2-digit number (1)" |
| service | 1 | "services (1)" |
| system | 1 | "the metric system (1)" |
| datum | 1 | "data (1)" |
| e | 1 | "e (1)" |
| fraction | 1 | "add fractions (1)" |
| student | 1 | "the student (1)" |
| HL | 1 | "functional hl (1)" |
| sting | 1 | "bee stings (1)" |
| vaccine | 1 | "a vaccine (1)" |
| option | 1 | "potential answer options (1)" |
| blank | 1 | "the first blank (1)" |
| accident | 1 | "accidents (1)" |
| disease | 1 | "diseases (1)" |
| cat | 1 | "rooms”, and “cats (1)" |
| Functional Health Literacy | 1 | "functional health literacy (1)" |
| NVS | 1 | "nvs (1)" |
| scenario | 1 | "scenario (1)" |
| report | 1 | "my health examination report (1)" |
| interpretation | 1 | "your interpretation (1)" |
| recognition | 1 | "word recognition (1)" |
| connection | 1 | "the connection (1)" |
| hl | 1 | "functional hl (1)" |
| hygiene | 1 | "personal hygiene (1)" |
| eyesight | 1 | "poor eyesight and good oral health (1)" |
| following | 1 | "the following (1)" |
| bowl | 1 | "a bowl (1)" |
| exercise | 1 | "exercise (1)" |
| substance | 1 | "the following substances (1)" |
| penicillin | 1 | "penicillin (1)" |
| latex | 1 | "latex gloves (1)" |
| glove | 1 | "latex gloves (1)" |
| bee | 1 | "bee stings (1)" |
| care | 1 | "health care (1)" |
| BMI | 1 | "bmi (1)" |

### Cognitive HL skills

| **Concept** | **Count** | **Noun Phrases** |
| --- | --- | --- |
| health | 150 | "health (38)" "your health (10)" "the health (8)" "health-related information (6)" "health information (4)" "health promotion (4)" "their health (4)" "lifelong health (3)" "the 4 health education settings (3)" "the health information (3)" "consumer health (2)" "health care (2)" "health care behaviors (2)" "health environment (2)" "health habits (2)" "health products (2)" "our health (2)" "personal and family health decisions (2)" "personal health decisions (2)" "suitable health products (2)" "accurate and meaningful health information (1)" "available health-related information (1)" "b) process health information (1)" "basic health knowledge (1)" "community health advocacy activities (1)" "everyday health habits (1)" "health behaviors (1)" "health care requirements (1)" "health concepts (1)" "health health mapping (1)" "health knowledge (1)" "health literacy skills (1)" "health literate (1)" "health practices (1)" "health pressures (1)" "health resources (1)" "health services (1)" "health skills (1)" "health-literate individuals (1)" "health-related actions (1)" "health-related life situations (1)" "health-related services (1)" "how appropriate and inappropriate health practices (1)" "major health issues (1)" "mental and emotional health (1)" "mental health (1)" "mental, emotional, social, and physical health (1)" "my health examination report (1)" "one's health (1)" "personal and family health (1)" "personal health (1)" "personal health behaviors (1)" "personal health goals (1)" "poor eyesight and good oral health (1)" "positive health practices (1)" "public health policies (1)" "regional health resources (1)" "significant community health issues (1)" "the health-related consequences (1)" "the provided health information (1)" "the term health (1)" "their own good health (1)" "their sexual health (1)" "valid health information (1)" "various health issues (1)" "various health plans/strategies (1)" "your health relate (1)" |
| information | 41 | "information (12)" "health-related information (6)" "health information (4)" "the health information (3)" "consumer information (2)" "accurate and meaningful health information (1)" "available health-related information (1)" "b) process health information (1)" "contact information (1)" "extract information (1)" "information sources (1)" "information/data (1)" "reliable information (1)" "the information (1)" "the provided health information (1)" "this information (1)" "valid health information (1)" "what information (1)" "which information (1)" |
| development | 20 | "development (12)" "sustainable development (2)" "growth development (1)" "individual development (1)" "mental and emotional development (1)" "particularly development (1)" "the development (1)" "the expected development (1)" |
| life | 18 | "life (5)" "life courses (2)" "their own lives (2)" "daily life (1)" "health-related life situations (1)" "life course stages (1)" "life cycle (1)" "life-threatening diseases (1)" "real life (1)" "the social life (1)" "their lives (1)" "your everyday life (1)" |
| hl | 18 | "functional hl (9)" "interactive hl (5)" "critical hl (3)" "vaccination hl (1)" |
| pupil | 18 | "pupils (16)" "pupils’ ability (1)" "pupils’ environmental awareness (1)" |
| student | 17 | "students (13)" "the student (4)" |
| disease | 17 | "disease prevention (8)" "disease (4)" "infectious disease (2)" "common infectious diseases (1)" "life-threatening diseases (1)" "spread/prevent diseases (1)" |
| product | 16 | "products (10)" "health products (2)" "suitable health products (2)" "a yogurt product (1)" "this product (1)" |
| service | 16 | "services (13)" "health services (1)" "health-related services (1)" "professional services (1)" |
| behavior | 16 | "behaviors (3)" "health care behaviors (2)" "health behaviors (1)" "healthcare behaviors (1)" "individual behaviors (1)" "negative and positive behaviors (1)" "one's own behavior (1)" "personal behavior (1)" "personal health behaviors (1)" "risk taking behaviors (1)" "risky and harmful behavior (1)" "risky and harmful behaviors (1)" "your behavior (1)" |
| skill | 16 | "skills (5)" "environmental protection skills (2)" "medication-taking skills (2)" "different coping skills (1)" "first aid skills (1)" "first-aid skills (1)" "health literacy skills (1)" "health skills (1)" "proper rejection skills (1)" "skill (1)" |
| school | 15 | "school (8)" "school environment (2)" "action-oriented school project (1)" "middle school (1)" "school map (1)" "the school (1)" "the school environment (1)" |
| example | 15 | "examples (10)" "example (2)" "an example (1)" "any other examples (1)" "at least 3 examples (1)" |
| child | 14 | "children (6)" "the children (5)" "child (1)" "child abuse (1)" "other children (1)" |
| community | 13 | "community (4)" "a beautiful community (1)" "communities (1)" "community health advocacy activities (1)" "community-based efforts (1)" "local communities (1)" "our community (1)" "our community environment (1)" "significant community health issues (1)" "the community (1)" |
| environment | 13 | "the environment (3)" "health environment (2)" "school environment (2)" "environment (1)" "our community environment (1)" "the future?school environment (1)" "the learning environment (1)" "the living environment (1)" "the school environment (1)" |
| growth | 13 | "growth (10)" "growth development (1)" "individual growth (1)" "physical, mental,emotional, and social growth (1)" |
| scenario | 13 | "scenario (13)" |
| influence | 12 | "the influence (9)" "their influence (2)" "what influences (1)" |
| food | 12 | "which food (3)" "healthy food (2)" "all healthy foods (1)" "food (1)" "food choices (1)" "foods (1)" "processed foods (1)" "some foods (1)" "the “food pyramid (1)" |
| grade | 12 | "grade (8)" "citizenship grade (1)" "citizinship grade (1)" "critical thinking grade (1)" "grade formulation (1)" |
| other | 11 | "others (10)" "us/others (1)" |
| doctor | 11 | "the doctor (5)" "your doctor (4)" "a doctor (2)" |
| prevention | 10 | "disease prevention (8)" "correct prevention strategy (1)" "prevention (1)" |
| family | 10 | "family (4)" "personal and family health decisions (2)" "family members (1)" "personal and family health (1)" "the family (1)" "their families (1)" |
| way | 9 | "ways (7)" "the way (1)" "various ways (1)" |
| practice | 9 | "practices (6)" "health practices (1)" "how appropriate and inappropriate health practices (1)" "positive health practices (1)" |
| action | 9 | "the action (2)" "action (1)" "action-oriented school project (1)" "actions (1)" "ethically responsible actions (1)" "health-related actions (1)" "these actions (1)" "which action (1)" |
| vaccination | 9 | "vaccination (3)" "vaccinations (3)" "oral vaccination/injection (1)" "the vaccination card (1)" "vaccination hl (1)" |
| activity | 8 | "physical activity (3)" "community health advocacy activities (1)" "leisure activities (1)" "our leisure activities (1)" "sexual activity (1)" "your daily activities (1)" |
| change | 8 | "these changes (2)" "which changes (2)" "change (1)" "changes (1)" "physical, mental, emotional, and social changes (1)" "the changes (1)" |
| ice | 8 | "this ice cream (4)" "ice cream (2)" "how much ice cream (1)" "this ice cream?skill (1)" |
| Pupils | 8 | "pupils (8)" |
| ability | 8 | "ability (6)" "pupils’ ability (1)" "the ability (1)" |
| medication | 7 | "medication education (2)" "medication-taking skills (2)" "his medication (1)" "medication (1)" "some medication (1)" |
| cream | 7 | "this ice cream (4)" "ice cream (2)" "how much ice cream (1)" |
| care | 7 | "care (2)" "health care (2)" "health care behaviors (2)" "health care requirements (1)" |
| situation | 7 | "potentially dangerous situations (3)" "situations (2)" "conflict situations (1)" "health-related life situations (1)" |
| tooth | 7 | "incisors/ erupting teeth/milk teeth (2)" "what teeth (2)" "your teeth (2)" "healthy teeth (1)" |
| cold | 7 | "a cold (6)" "my cold (1)" |
| diet | 7 | "a healthy diet (3)" "diet (3)" "your diet (1)" |
| people | 7 | "people (3)" "other people (2)" "all people (1)" "different people (1)" |
| education | 7 | "the 4 health education settings (3)" "medication education (2)" "sex education (2)" |
| thought | 7 | "your thoughts (6)" "their thoughts (1)" |
| consequence | 6 | "the consequences (2)" "a) distinguish short- and long-term consequences (1)" "the health-related consequences (1)" "these consequences (1)" "what consequences (1)" |
| aspect | 6 | "aspects (3)" "the aspects (1)" "the various aspects (1)" "those aspects (1)" |
| assessment | 6 | "assessment (5)" "self-assessment (1)" |
| role | 6 | "a positive, active role (1)" "different roles (1)" "role-playing games (1)" "the role (1)" "the roles (1)" "tobacco-smoking?which role (1)" |
| decision | 6 | "personal and family health decisions (2)" "personal health decisions (2)" "our decision-making collective?is (1)" "their individual decision-making (1)" |
| process | 6 | "the process (4)" "b) process health information (1)" "participatory dialogue process (1)" |
| interaction | 6 | "interpersonal interactions (4)" "the interaction (2)" |
| effect | 6 | "the effects (2)" "effect (1)" "effects (1)" "the cause/effect (1)" "the nutritive effect (1)" |
| individual | 6 | "health-literate individuals (3)" "individuals (2)" "all individuals (1)" |
| understanding | 6 | "an understanding (2)" "understanding (2)" "this understanding (1)" "your understanding (1)" |
| knowledge | 5 | "knowledge (2)" "basic health knowledge (1)" "health knowledge (1)" "the knowledge (1)" |
| parent | 5 | "your parents (3)" "my parents (2)" |
| alternative | 5 | "which alternatives (3)" "these alternatives (1)" "various alternatives (1)" |
| source | 5 | "different sources (2)" "information sources (1)" "some reliable sources (1)" "sources (1)" |
| label | 5 | "this label (4)" "label (1)" |
| resource | 5 | "a resource (1)" "a resource-based manner (1)" "electronic resources (1)" "health resources (1)" "regional health resources (1)" |
| strategy | 5 | "appropriate conflict resolution strategies (1)" "correct prevention strategy (1)" "strategies (1)" "various health plans/strategies (1)" "your coping strategies (1)" |
| consumer | 5 | "consumer health (2)" "consumer information (2)" "a selective consumer (1)" |
| pain | 5 | "chronic pain (1)" "pain (1)" "pain visit (1)" "the most frequent pain (1)" "their pain (1)" |
| protection | 5 | "correct personal protection (2)" "environmental protection skills (2)" "environmental protection (1)" |
| peanut | 5 | "peanuts (3)" "peanut oil (2)" |
| lot | 5 | "a lot (5)" |
| promotion | 5 | "health promotion (4)" "promotion (1)" |
| classmate | 5 | "my classmates (3)" "classmates (1)" "my classmate (1)" |
| impact | 5 | "the impact (2)" "the media impact (1)" "the media's impact (1)" "the possible impact (1)" |
| factor | 4 | "factors (2)" "analyse factors (1)" "multiple factors (1)" |
| relationship | 4 | "causal relationships (2)" "peer relationships (1)" "positive relationships (1)" |
| surrounding | 4 | "their surroundings (3)" "one's immediate surroundings (1)" |
| body | 4 | "body autonomy (1)" "body temperature (1)" "the body (1)" "various interrelated body systems (1)" |
| choice | 4 | "food choices (2)" "choices (1)" "one's own choices (1)" |
| hygiene | 4 | "personal hygiene (3)" "good personal hygiene (1)" |
| fat | 4 | "saturated fat (3)" "fat (1)" |
| responsibility | 4 | "personal responsibility (3)" "responsibilities (1)" |
| mask | 4 | "a mask (1)" "his sanitary mask (1)" "my sanitary mask (1)" "sanitary masks (1)" |
| issue | 4 | "major health issues (1)" "significant community health issues (1)" "this issue (1)" "various health issues (1)" |
| age | 4 | "2 age (1)" "3 age (1)" "3 age 8-9, multiply 2-digit number (1)" "age (1)" |
| state | 4 | "state (2)" "a desirable state (1)" "the local and state levels (1)" |
| puberty | 4 | "puberty (4)" |
| Health | 4 | "health-literate individuals (2)" "health (1)" "health health mapping (1)" |
| perspective | 4 | "personal and collective perspective (4)" |
| group | 4 | "a group (2)" "groups (1)" "peer group (1)" |
| being | 4 | "well-being (4)" |
| medicine | 4 | "your medicine (3)" "medicines (1)" |
| behaviour | 4 | "behaviour (2)" "polite behaviour (1)" "your behaviour (1)" |
| risk | 4 | "risk (1)" "risk taking behaviors (1)" "risks (1)" "the risk (1)" |
| concept | 4 | "the concept (2)" "concepts (1)" "health concepts (1)" |
| friendship | 4 | "friendship (1)" "harmonious friendships (1)" "my friendships (1)" "opposite-sex friendships (1)" |
| sex | 4 | "sex education (2)" "opposite-sex friendships (1)" "the opposite sex (1)" |
| aid | 4 | "aids (1)" "first aid (1)" "first aid skills (1)" "first-aid skills (1)" |
| drug | 4 | "drugs (1)" "illegal drugs (1)" "other drugs (1)" "pharmaceutical drugs (1)" |
| view | 3 | "different views (1)" "various views (1)" "your schoolmates’ view (1)" |
| agency | 3 | "at least 3 agencies (2)" "government agencies (1)" |
| setting | 3 | "the 4 health education settings (3)" |
| respect | 3 | "respect (3)" |
| peer | 3 | "peer group (1)" "peer relationships (1)" "their peers (1)" |

### Interactive HL skills

| **Concept** | **Count** | **Noun Phrases** |
| --- | --- | --- |
| health | 19 | "health-related life situations (3)" "mental health (3)" "your health (3)" "personal health (2)" "age-appropriate health-related vocabulary (1)" "equitable health care (1)" "good health (1)" "good health care (1)" "health advocacy strategies (1)" "health information (1)" "health issues (1)" "the health information (1)" |
| communication | 16 | "communication (5)" "communication skills (2)" "effective communication (2)" "effective interpersonal communication skills (2)" "appropriate communication techniques (1)" "effective communication skills (1)" "healthy communication and resolution strategies (1)" "interpersonal communication skills (1)" "various communication techniques (1)" |
| skill | 12 | "proper rejection skills (3)" "communication skills (2)" "effective interpersonal communication skills (2)" "effective communication skills (1)" "effective emotional management skills (1)" "interpersonal communication skills (1)" "skills (1)" "their emotional and interaction skills (1)" |
| interaction | 11 | "interpersonal interactions (8)" "social interaction (1)" "the interaction (1)" "their emotional and interaction skills (1)" |
| situation | 11 | "health-related life situations (3)" "potentially harmful situations (2)" "age-appropriate situations (1)" "behaviors/situations (1)" "conflict situations (1)" "different conflict and crisis situations (1)" "everyday situations (1)" "interactive situations (1)" |
| other | 10 | "others (10)" |
| student | 8 | "students (8)" |
| conflict | 8 | "conflict (2)" "conflicts (2)" "appropriate conflict resolution strategies (1)" "conflict situations (1)" "different conflict and crisis situations (1)" "effective conflict resolution strategies (1)" |
| way | 8 | "ways (4)" "healthy ways (2)" "model appropriate ways (1)" "the ways (1)" |
| hl | 7 | "interactive hl (7)" |
| feeling | 7 | "feelings (6)" "the feeling (1)" |
| community | 6 | "community (3)" "communities (2)" "a healthy community (1)" |
| pupil | 6 | "pupils (5)" "pupils opportunities (1)" |
| family | 6 | "family (3)" "families (2)" "their families (1)" |
| friend | 6 | "a friend (1)" "friends (1)" "my friend (1)" "my friends (1)" "your friend’s request (1)" "your friends (1)" |
| parent | 6 | "your parents (6)" |
| sex | 5 | "sex education (2)" "opposite-sex friendships (1)" "sex (1)" "the opposite sex (1)" |
| scenario | 5 | "scenario (5)" |
| response | 5 | "your response (5)" |
| life | 5 | "health-related life situations (3)" "their life (1)" "their lives (1)" |
| need | 5 | "needs (4)" "the need (1)" |
| grade | 5 | "grade (5)" |
| behavior | 4 | "behaviors (2)" "behaviors/situations (1)" "negative and positive behaviors (1)" |
| school | 4 | "school (2)" "schools (2)" |
| strategy | 4 | "appropriate conflict resolution strategies (1)" "effective conflict resolution strategies (1)" "health advocacy strategies (1)" "healthy communication and resolution strategies (1)" |
| emotion | 4 | "emotions (2)" "their emotions (1)" "various emotions (1)" |
| role | 4 | "different roles (2)" "role-playing games (1)" "the role (1)" |
| doctor | 4 | "your doctor (3)" "doctors (1)" |
| people | 4 | "other people (4)" |
| education | 4 | "medication education (2)" "sex education (2)" |
| friendship | 4 | "harmonious friendships (2)" "my friendships (1)" "opposite-sex friendships (1)" |
| child | 4 | "the children (3)" "other children (1)" |
| peer | 3 | "peers (3)" |
| resolution | 3 | "appropriate conflict resolution strategies (1)" "effective conflict resolution strategies (1)" "healthy communication and resolution strategies (1)" |
| care | 3 | "care (1)" "equitable health care (1)" "good health care (1)" |
| behaviour | 3 | "behaviour (2)" "polite behaviour (1)" |
| example | 3 | "example (2)" "examples (1)" |
| nutrition | 3 | "healthy nutrition (3)" |
| issue | 3 | "a tough issue (1)" "health issues (1)" "tough issues (1)" |
| mother | 3 | "his mother (1)" "my mother (1)" "your mother’s worry (1)" |
| rejection | 3 | "proper rejection skills (3)" |
| medication | 3 | "medication education (2)" "his medication (1)" |
| self | 3 | "self (3)" |
| classmate | 3 | "classmates (1)" "my classmate (1)" "my classmates (1)" |
| group | 2 | "a group (2)" |
| crisis | 2 | "crises (1)" "different conflict and crisis situations (1)" |
| practice | 2 | "practices (1)" "support practices (1)" |
| model | 2 | "c) model (1)" "model appropriate ways (1)" |
| age | 2 | "age-appropriate health-related vocabulary (1)" "age-appropriate situations (1)" |
| stress | 2 | "stress (1)" "the stress (1)" |
| technique | 2 | "appropriate communication techniques (1)" "various communication techniques (1)" |
| bridge | 2 | "bridges (2)" |
| barrier | 2 | "barriers (2)" |
| choice | 2 | "healthy choices (2)" |
| relationship | 2 | "healthy relationships (1)" "relationships (1)" |
| aspect | 2 | "another aspect (1)" "the positive and negative aspects (1)" |
| place | 2 | "our class win second place (1)" "place (1)" |
| method | 2 | "emotional management methods (1)" "methods (1)" |
| technology | 2 | "interactive technology (2)" |
| information | 2 | "health information (1)" "the health information (1)" |
| boy | 2 | "a boy (1)" "boys’ trousers (1)" |
| body | 2 | "body autonomy (2)" |
| game | 2 | "role-playing games (1)" "the game (1)" |
| autonomy | 2 | "body autonomy (2)" |
| appreciation | 2 | "appreciation (2)" |
| encouragement | 2 | "encouragement (2)" |
| John | 2 | "john (1)" "john’s kindness (1)" |
| party | 2 | "a party (1)" "our party (1)" |
| management | 2 | "effective emotional management skills (1)" "emotional management methods (1)" |
| performance | 1 | "andy’s excellent performance (1)" |
| win | 1 | "our class win second place (1)" |
| Andy | 1 | "andy’s excellent performance (1)" |
| class | 1 | "our class win second place (1)" |
| throat | 1 | "a sore throat (1)" |
| karaoke | 1 | "a karaoke bar (1)" |
| kindness | 1 | "john’s kindness (1)" |
| today | 1 | "today (1)" |
| graduation | 1 | "graduation ceremony (1)" |
| ceremony | 1 | "graduation ceremony (1)" |
| runner | 1 | "the last runner (1)" |
| bar | 1 | "a karaoke bar (1)" |
| condition | 1 | "my condition (1)" |
| sadness | 1 | "may’s sadness (1)" |
| day | 1 | "the day (1)" |
| race | 1 | "the relay race (1)" |
| relay | 1 | "the relay race (1)" |
| meet | 1 | "the sports meet (1)" |
| sport | 1 | "the sports meet (1)" |
| joke | 1 | "gender-related jokes (1)" |
| gender | 1 | "gender-related jokes (1)" |
| thought | 1 | "your thoughts (1)" |
| strap | 1 | "girls’ bra straps (1)" |
| bra | 1 | "girls’ bra straps (1)" |
| girl | 1 | "girls’ bra straps (1)" |
| trouser | 1 | "boys’ trousers (1)" |
| fun | 1 | "fun (1)" |
| member | 1 | "members (1)" |
| kind | 1 | "this kind (1)" |
| May | 1 | "may’s sadness (1)" |
| worry | 1 | "your mother’s worry (1)" |

### Critical HL skills

| **Concept** | **Count** | **Noun Phrases** |
| --- | --- | --- |
| health | 130 | "health (14)" "health information (9)" "their health (6)" "health-related information (4)" "lifelong health (4)" "the health (4)" "good health (3)" "personal health (3)" "your health (3)" "consumer health (2)" "health care requirements (2)" "health habits (2)" "health practices (2)" "health products (2)" "health promotion (2)" "life-long health (2)" "mental health (2)" "our health (2)" "personal and family health decisions (2)" "personal health progress (2)" "public health policies (2)" "suitable health products (2)" "the health information (2)" "a health agency (1)" "accurate and meaningful health information (1)" "available health-related information (1)" "community and environmental health issues (1)" "community and environmental health plans/strategies (1)" "community health advocacy activities (1)" "community health plans (1)" "critically appraising health information (1)" "dental health literacy (1)" "different health plans (1)" "environmental health (1)" "equitable health care (1)" "good health care (1)" "health advocacy (1)" "health advocacy strategies (1)" "health behaviors (1)" "health care (1)" "health communications (1)" "health environment (1)" "health goals (1)" "health health mapping (1)" "health issues (1)" "health literate (1)" "health pressures (1)" "health resources (1)" "health services (1)" "health-promoting foods (1)" "health-related decision-making (1)" "health-related knowledge (1)" "health-related life situations (1)" "health-related services (1)" "how appropriate and inappropriate health practices (1)" "major health issues (1)" "mental and emotional health (1)" "mental, emotional, social, and physical health (1)" "non-health-promoting foods (1)" "past health plans/strategies (1)" "personal and family health (1)" "personal health behaviors (1)" "personal health goals (1)" "positive health practices (1)" "regional health resources (1)" "significant community health issues (1)" "the 4 health education settings (1)" "the health topics (1)" "their own health decisions (1)" "their sexual health (1)" "true and false health information (1)" "various health issues (1)" "various health plans/strategies (1)" "what specific health information (1)" "your own health (1)" |
| information | 35 | "health information (9)" "information (7)" "health-related information (4)" "the health information (2)" "accurate and meaningful health information (1)" "available health-related information (1)" "consumer information (1)" "critically appraising health information (1)" "information sources (1)" "information/data (1)" "new information (1)" "reliable information (1)" "the information (1)" "the most wrong information (1)" "this information (1)" "true and false health information (1)" "what specific health information (1)" |
| student | 21 | "students (16)" "the student (5)" |
| service | 18 | "services (15)" "health services (1)" "health-related services (1)" "professional services (1)" |
| product | 18 | "products (12)" "health products (2)" "suitable health products (2)" "a yogurt product (1)" "this product (1)" |
| community | 18 | "community (5)" "communities (3)" "a healthy community (1)" "community and environmental health issues (1)" "community and environmental health plans/strategies (1)" "community health advocacy activities (1)" "community health plans (1)" "community-based efforts (1)" "local communities (1)" "our community (1)" "significant community health issues (1)" "the community (1)" |
| food | 17 | "food (2)" "the foods (2)" "different food groups (1)" "fake food (1)" "food choices (1)" "food shopping (1)" "food-impulses (1)" "foods (1)" "health-promoting foods (1)" "healthy food (1)" "non-health-promoting foods (1)" "right balanced food (1)" "the two food categories (1)" "their regulary consumed food (1)" "various foods (1)" |
| way | 16 | "ways (9)" "the way (3)" "the ways (2)" "my way (1)" "various ways (1)" |
| pupil | 16 | "pupils (14)" "pupils’ ability (1)" "pupils’ environmental awareness (1)" |
| school | 15 | "school (6)" "school environment (2)" "schools (2)" "action-oriented school project (1)" "middle school (1)" "school map (1)" "the school (1)" "the school environment (1)" |
| behavior | 15 | "behaviors (5)" "age-appropriate behaviors (1)" "behaviors/situations (1)" "everyday behavior (1)" "health behaviors (1)" "individual behavior (1)" "personal health behaviors (1)" "positive, personal hygienic behaviors (1)" "risk taking behaviors (1)" "risky and harmful behaviors (1)" "their behaviors (1)" |
| situation | 14 | "potentially dangerous situations (3)" "situations (3)" "potentially harmful situations (2)" "the situation (2)" "behaviors/situations (1)" "everyday situations (1)" "health-related life situations (1)" "what everyday situations (1)" |
| example | 13 | "examples (5)" "an example (2)" "any other examples (2)" "example (2)" "at least 2 examples (1)" "at least 3 examples (1)" |
| family | 13 | "family (5)" "families (2)" "personal and family health decisions (2)" "their families (2)" "family members (1)" "personal and family health (1)" |
| hl | 13 | "critical hl (6)" "functional hl (3)" "interactive hl (3)" "communicative hl (1)" |
| skill | 13 | "communication skills (2)" "different coping skills (2)" "effective emotional management skills (2)" "effective communication skills (1)" "effective interpersonal communication skills (1)" "environmental protection skills (1)" "interpersonal and decision-making skills (1)" "medication-taking skills (1)" "proper rejection skills (1)" "skills (1)" |
| environment | 11 | "environment (2)" "school environment (2)" "environments (1)" "health environment (1)" "the environment (1)" "the future?school environment (1)" "the learning environment (1)" "the living environment (1)" "the school environment (1)" |
| practice | 11 | "practices (5)" "health practices (2)" "how appropriate and inappropriate health practices (1)" "personal practices (1)" "positive health practices (1)" "support practices (1)" |
| safety | 11 | "safety (6)" "road safety lessons (3)" "outdoor safety tips (1)" "safety tips (1)" |
| disease | 10 | "disease prevention (5)" "disease (4)" "spread/prevent diseases (1)" |
| child | 10 | "the children (5)" "children (3)" "child abuse (1)" "each child (1)" |
| scenario | 10 | "scenario (10)" |
| life | 9 | "life (3)" "life events (2)" "life-long health (2)" "health-related life situations (1)" "their own lives (1)" |
| parent | 9 | "my parents (4)" "your parents (3)" "parents (1)" "their parents (1)" |
| decision | 9 | "personal and family health decisions (2)" "decisions (1)" "health-related decision-making (1)" "interpersonal and decision-making skills (1)" "our decision-making collective?is (1)" "our final decision (1)" "their individual decision-making (1)" "their own health decisions (1)" |
| choice | 9 | "choices (2)" "diet choices (2)" "food choices (2)" "healthy choices (2)" "your snack choices (1)" |
| influence | 8 | "the influence (5)" "their influence (2)" "what influences (1)" |
| other | 8 | "others (7)" "us/others (1)" |
| action | 8 | "the action (2)" "action (1)" "action-oriented school project (1)" "actions (1)" "ethically responsible actions (1)" "these actions (1)" "which action (1)" |
| diet | 8 | "a healthy diet (3)" "a healthy, balanced diet (2)" "diet choices (2)" "diet (1)" |
| strategy | 8 | "strategies (2)" "community and environmental health plans/strategies (1)" "effective conflict resolution strategies (1)" "health advocacy strategies (1)" "past health plans/strategies (1)" "various health plans/strategies (1)" "your coping strategies (1)" |
| grade | 8 | "grade (4)" "citizenship grade (1)" "citizinship grade (1)" "critical thinking grade (1)" "grade formulation (1)" |
| Pupils | 7 | "pupils (7)" |
| change | 7 | "these changes (2)" "which changes (2)" "change (1)" "changes (1)" "the changes (1)" |
| prevention | 7 | "disease prevention (5)" "the prevention (2)" |
| communication | 7 | "communication skills (2)" "appropriate communication techniques (1)" "effective communication (1)" "effective communication skills (1)" "effective interpersonal communication skills (1)" "health communications (1)" |
| issue | 6 | "community and environmental health issues (1)" "health issues (1)" "major health issues (1)" "significant community health issues (1)" "this issue (1)" "various health issues (1)" |
| responsibility | 6 | "personal responsibility (5)" "responsibilities (1)" |
| development | 6 | "sustainable development (2)" "the development (2)" "development (1)" "the item development process (1)" |
| impact | 6 | "the impact (4)" "the media impact (1)" "the possible impact (1)" |
| care | 5 | "health care requirements (2)" "equitable health care (1)" "good health care (1)" "health care (1)" |
| medication | 5 | "medication education (2)" "medication (1)" "medication-taking skills (1)" "some medication (1)" |
| resource | 5 | "resources (2)" "health resources (1)" "regional health resources (1)" "the resources (1)" |
| plan | 5 | "community and environmental health plans/strategies (1)" "community health plans (1)" "different health plans (1)" "past health plans/strategies (1)" "various health plans/strategies (1)" |
| relationship | 5 | "causal relationships (2)" "healthy relationships (1)" "peer relationships (1)" "positive relationships (1)" |
| lot | 5 | "a lot (5)" |
| agency | 5 | "government agencies (2)" "the assigned agencies (2)" "a health agency (1)" |
| group | 5 | "a group (2)" "different food groups (1)" "each group (1)" "groups (1)" |
| consequence | 5 | "the consequences (2)" "a) distinguish short- and long-term consequences (1)" "these consequences (1)" "what consequences (1)" |
| risk | 5 | "risk (2)" "risk taking behaviors (1)" "risks (1)" "the risk (1)" |
| alternative | 5 | "which alternatives (3)" "these alternatives (1)" "various alternatives (1)" |
| doctor | 4 | "the doctor (3)" "doctor (1)" |
| policy | 4 | "policies (2)" "public health policies (2)" |
| factor | 4 | "factors (2)" "analyse factors (1)" "multiple factors (1)" |
| goal | 4 | "common goals (1)" "goals (1)" "health goals (1)" "personal health goals (1)" |
| behaviour | 4 | "behaviour (3)" "polite behaviour (1)" |
| fruit | 4 | "fruits (3)" "different fruits (1)" |
| method | 4 | "emotional management methods (2)" "different methods (1)" "methods (1)" |
| management | 4 | "effective emotional management skills (2)" "emotional management methods (2)" |
| consumer | 4 | "consumer health (2)" "a selective consumer (1)" "consumer information (1)" |
| assessment | 4 | "assessment (3)" "self-assessment (1)" |
| nutrition | 4 | "nutrition (2)" "a nutrition (1)" "nutrition labels (1)" |
| snack | 4 | "snacks (2)" "cariogenic snacks (1)" "your snack choices (1)" |
| sport | 4 | "a sports club (2)" "sports (1)" "the sports meet (1)" |
| role | 4 | "a positive, active role (1)" "different roles (1)" "the role (1)" "the roles (1)" |
| treatment | 4 | "correct medical treatment (2)" "prescribed/recommended treatment (1)" "treatment (1)" |
| activity | 4 | "physical activity (2)" "community health advocacy activities (1)" "sexual activity (1)" |
| control | 4 | "greater control (2)" "control (1)" "some control (1)" |
| state | 4 | "state (2)" "a desirable state (1)" "the local and state levels (1)" |
| thought | 4 | "your thoughts (4)" |
| response | 3 | "your response (3)" |
| need | 3 | "individual needs (2)" "the need (1)" |
| week | 3 | "week (2)" "one week (1)" |
| toothpaste | 3 | "toothpaste (2)" "the toothpaste (1)" |
| time | 3 | "time (2)" "times (1)" |
| emotion | 3 | "emotions (2)" "various emotions (1)" |
| thinking | 3 | "critical thinking (2)" "critical thinking grade (1)" |
| stress | 3 | "stress (3)" |
| study | 3 | "further studies (1)" "small study units (1)" "such studies (1)" |
| basis | 3 | "a basis (1)" "a regular basis (1)" "the basis (1)" |
| surrounding | 3 | "their surroundings (3)" |
| game | 3 | "a relay game (1)" "another so-called energy game (1)" "the game (1)" |
| conflict | 3 | "conflict (1)" "conflicts (1)" "effective conflict resolution strategies (1)" |
| day | 3 | "each day (1)" "not every day (1)" "the day (1)" |
| making | 3 | "health-related decision-making (1)" "our decision-making collective?is (1)" "their individual decision-making (1)" |
| lesson | 3 | "road safety lessons (3)" |
| road | 3 | "road safety lessons (3)" |
| medicine | 3 | "your medicine (3)" |
| peer | 3 | "peer relationships (1)" "peers (1)" "their peers (1)" |
| institution | 3 | "institutions (2)" "institution (1)" |
| organization | 3 | "organizations (2)" "organization (1)" |
| education | 3 | "medication education (2)" "the 4 health education settings (1)" |
| being | 3 | "well-being (2)" "well being (1)" |
| relation | 3 | "relations (2)" "relation (1)" |
| experience | 3 | "experience (1)" "our experience (1)" "their own experiences (1)" |
| barrier | 3 | "which barriers (2)" "barriers (1)" |
| process | 3 | "participatory dialogue process (1)" "the item development process (1)" "the process (1)" |
| instruction | 3 | "instruction (2)" "objective instruction (1)" |
| friend | 3 | "my friend (1)" "my friends (1)" "your friend’s request (1)" |
| reliability | 3 | "the reliability (3)" |

stylefix
