# Supplementary material for: Health literacy in the context of child health promotion: a scoping review of conceptualizations and descriptions
Source: BMC Public Health. 2024 Mar 14;24:808. doi: 10.1186/s12889-024-17955-7 (PMC10941366; doi:10.1186/s12889-024-17955-7)

# Supplementary material 4: Health literacy in the context of child health promotion: a scoping review of conceptualizations and descriptions

## Search history original search, 11 July 2020

For all databases search terms regarding the construct #1 and #2, the population #3 and #4, the methods/tools used #5 and the document criteria #6. Combinations of search terms were tested to find to most valuable search string. This search string is presented in **bold.**

|  | Medline/PubMed | Embase.com | Cinahl | Psycinfo | Eric |
| --- | --- | --- | --- | --- | --- |
| #1 | "Health Literacy"[Mesh] OR “health literac*”[tiab] OR “ehealth literac*”[tiab] OR “physical literac*”[tiab] OR “oral literac*”[tiab] OR “nutrition literac*”[tiab] OR “nutritional literac*”[tiab] OR “food literac*”[tiab] OR “media literac*”[tiab] | 'health literacy'/exp OR ((health OR ehealth OR physical OR oral OR nutrition* OR food OR media) NEXT/2 literac*):ti,ab,kw | MH "Health Literacy" OR TI((health OR ehealth OR physical OR oral OR nutrition* OR food OR media) N**1** literac*) OR AB((health OR ehealth OR physical OR oral OR nutrition* OR food OR media) N**1** literac*) OR KW((health OR ehealth OR physical OR oral OR nutrition* OR food OR media) N**1** literac*) | DE "Health Literacy" OR TI((health OR ehealth OR physical OR oral OR nutrition* OR food OR media) N1 literac*) OR AB((health OR ehealth OR physical OR oral OR nutrition* OR food OR media) N1 literac*) OR KW((health OR ehealth OR physical OR oral OR nutrition* OR food OR media) N1 literac*) | TI((health OR ehealth OR physical OR oral OR nutrition* OR food OR media) N1 literac*) OR AB((health OR ehealth OR physical OR oral OR nutrition* OR food OR media) N1 literac*) OR KW((health OR ehealth OR physical OR oral OR nutrition* OR food OR media) N1 literac*) |
| #2 | ((alcohol[tiab] OR drinking[tiab] OR drug*[tiab] OR cigarette*[tiab] OR smoking[tiab] OR tobacco[tiab] OR sexual*[tiab] OR sex[tiab] OR hiv[tiab] OR sun[tiab] OR sunscreen*[tiab] OR uv[tiab] OR “skin protect*”[tiab] OR fitness[tiab] OR “screen time”[tiab] OR “screen use”[tiab] OR hygien*[tiab]) AND literac*[tiab]) | ((alcohol OR drinking OR drug* OR cigarette* OR smoking OR tobacco OR sexual* OR sex OR hiv OR sun OR sunscreen* OR uv OR “skin protect*” OR hiv OR fitness OR “screen time” OR “screen use” OR hygiene) NEAR/6 literac*):ti,ab,kw | TI((alcohol OR drinking OR drug* OR cigarette* OR smoking OR tobacco OR sexual* OR sex OR hiv OR sun OR sunscreen* OR uv OR “skin protect*” OR hiv OR fitness OR “screen time” OR “screen use” OR hygiene) W5 literac*) OR AB ((alcohol OR drinking OR drug* OR cigarette* OR smoking OR tobacco OR sexual* OR sex OR hiv OR sun OR sunscreen* OR uv OR “skin protect*” OR hiv OR fitness OR “screen time” OR “screen use” OR hygiene) W5 literac*) OR KW((alcohol OR drinking OR drug* OR cigarette* OR smoking OR tobacco OR sexual* OR sex OR hiv OR sun OR sunscreen* OR uv OR “skin protect*” OR hiv OR fitness OR “screen time” OR “screen use” OR hygiene) W5 literac*) | TI((alcohol OR drinking OR drug* OR cigarette* OR smoking OR tobacco OR sexual* OR sex OR hiv OR sun OR sunscreen* OR uv OR “skin protect*” OR hiv OR fitness OR “screen time” OR “screen use” OR hygiene) W5 literac*) OR AB ((alcohol OR drinking OR drug* OR cigarette* OR smoking OR tobacco OR sexual* OR sex OR hiv OR sun OR sunscreen* OR uv OR “skin protect*” OR hiv OR fitness OR “screen time” OR “screen use” OR hygiene) W5 literac*) OR KW((alcohol OR drinking OR drug* OR cigarette* OR smoking OR tobacco OR sexual* OR sex OR hiv OR sun OR sunscreen* OR uv OR “skin protect*” OR hiv OR fitness OR “screen time” OR “screen use” OR hygiene) W5 literac*) | TI((alcohol OR drinking OR drug* OR cigarette* OR smoking OR tobacco OR sexual* OR sex OR hiv OR sun OR sunscreen* OR uv OR “skin protect*” OR hiv OR fitness OR “screen time” OR “screen use” OR hygiene) W5 literac*) OR AB ((alcohol OR drinking OR drug* OR cigarette* OR smoking OR tobacco OR sexual* OR sex OR hiv OR sun OR sunscreen* OR uv OR “skin protect*” OR hiv OR fitness OR “screen time” OR “screen use” OR hygiene) W5 literac*) OR KW((alcohol OR drinking OR drug* OR cigarette* OR smoking OR tobacco OR sexual* OR sex OR hiv OR sun OR sunscreen* OR uv OR “skin protect*” OR hiv OR fitness OR “screen time” OR “screen use” OR hygiene) W5 literac*) |
| #3 | child*[tiab] OR adolescen*[tiab] OR schoolchild*[tiab] OR teenage*[tiab] OR teen[tiab] OR teens[tiab] OR youth[tiab] OR youths[tiab] OR “pre-teen*”[tiab] OR boy[tiab] OR boys[tiab] OR girl[tiab] OR girls[tiab] OR “young people”[tiab] OR school*[tiab] OR “afterschool”[tiab] OR “junior high”[tiab] | (child* OR adolescen* OR schoolchild* OR teenage* OR teen OR teens OR youth OR youths OR “pre-teen*” OR boy OR boys OR girl OR girls OR “young people” OR school* OR afterschool OR “junior high”):ti,ab,kw | TI(child* OR adolescen* OR schoolchild* OR teenage* OR teen OR teens OR youth OR youths OR “pre-teen*” OR boy OR boys OR girl OR girls OR “young people” OR “school*” OR afterschool OR “junior high”) OR AB(child* OR adolescen* OR schoolchild* OR teenage* OR teen OR teens OR youth OR youths OR “pre-teen*” OR boy OR boys OR girl OR girls OR “young people” OR “school*” OR afterschool OR “junior high”) OR KW(child* OR adolescen* OR schoolchild* OR teenage* OR teen OR teens OR youth OR youths OR “pre-teen*” OR boy OR boys OR girl OR girls OR “young people” OR “school*” OR afterschool OR “junior high”) | TI(child* OR adolescen* OR schoolchild* OR teenage* OR teen OR teens OR youth OR youths OR “pre-teen*” OR boy OR boys OR girl OR girls OR “young people” OR “school*” OR afterschool OR “junior high”) OR AB(child* OR adolescen* OR schoolchild* OR teenage* OR teen OR teens OR youth OR youths OR “pre-teen*” OR boy OR boys OR girl OR girls OR “young people” OR “school*” OR afterschool OR “junior high”) OR KW(child* OR adolescen* OR schoolchild* OR teenage* OR teen OR teens OR youth OR youths OR “pre-teen*” OR boy OR boys OR girl OR girls OR “young people” OR “school*” OR afterschool OR “junior high”) | TI(child* OR adolescen* OR schoolchild* OR teenage* OR teen OR teens OR youth OR youths OR “pre-teen*” OR boy OR boys OR girl OR girls OR “young people” OR “school*” OR afterschoolOR “junior high”) OR AB(child* OR adolescen* OR schoolchild* OR teenage* OR teen OR teens OR youth OR youths OR “pre-teen*” OR boy OR boys OR girl OR girls OR “young people” OR “school*” Or afterschool OR “junior high”) OR KW(child* OR adolescen* OR schoolchild* OR teenage* OR teen OR teens OR youth OR youths OR “pre-teen*” OR boy OR boys OR girl OR girls OR “young people” OR “school*” OR afterschool OR “junior high”) |
| #4 | “grade 4”[tiab] OR “grade 5”[tiab] OR “grade 6”[tiab] OR “grade 7“[tiab] OR “grade 8”[tiab] OR “grades 4”[tiab] OR “grades 5”[tiab] OR “grades 6”[tiab] OR “grades 7“[tiab] OR “grades 8”[tiab] OR “4th grade*“[tiab] OR “fourth grade*“[tiab] OR “5th grade*“[tiab] OR “fifth grade*“[tiab] OR “6th grade*“[tiab] OR “sixth grade*“[tiab] OR “7th grade*“[tiab] OR “seventh grade*“[tiab] OR “8th grade*“[tiab] OR “eighth grade*“[tiab] | (grade* NEAR/1 (4 OR 5 OR 6 OR 7 OR 8 OR 4th OR 5th OR 6th OR 7th OR 8th OR fourth OR fifth OR sixth OR seventh OR eighth)):ti,ab,kw | TI(grade* W1 (4 OR 5 OR 6 OR 7 OR 8 OR 4th OR 5th OR 6th OR 7th OR 8th OR fourth OR fifth OR sixth OR seventh OR eighth)) OR AB(grade* W1 (4 OR 5 OR 6 OR 7 OR 8 OR 4th OR 5th OR 6th OR 7th OR 8th OR fourth OR fifth OR sixth OR seventh OR eighth)) OR KW(grade* W1 (4 OR 5 OR 6 OR 7 OR 8 OR 4th OR 5th OR 6th OR 7th OR 8th OR fourth OR fifth OR sixth OR seventh OR eighth) | TI(grade* W1 (4 OR 5 OR 6 OR 7 OR 8 OR 4th OR 5th OR 6th OR 7th OR 8th OR fourth OR fifth OR sixth OR seventh OR eighth)) OR AB(grade* W1 (4 OR 5 OR 6 OR 7 OR 8 OR 4th OR 5th OR 6th OR 7th OR 8th OR fourth OR fifth OR sixth OR seventh OR eighth)) OR KW(grade* W1 (4 OR 5 OR 6 OR 7 OR 8 OR 4th OR 5th OR 6th OR 7th OR 8th OR fourth OR fifth OR sixth OR seventh OR eighth) | TI(grade* W1 (4 OR 5 OR 6 OR 7 OR 8 OR 4th OR 5th OR 6th OR 7th OR 8th OR fourth OR fifth OR sixth OR seventh OR eighth)) OR AB(grade* W1 (4 OR 5 OR 6 OR 7 OR 8 OR 4th OR 5th OR 6th OR 7th OR 8th OR fourth OR fifth OR sixth OR seventh OR eighth)) OR KW(grade* W1 (4 OR 5 OR 6 OR 7 OR 8 OR 4th OR 5th OR 6th OR 7th OR 8th OR fourth OR fifth OR sixth OR seventh OR eighth) |
| #5 | "Surveys and Questionnaires"[Mesh] OR survey*[tiab] OR questionnaire*[tiab] OR instrument*[tiab] OR assessment*[tiab] OR measure*[tiab] OR “screening tool*”[tiab] OR psychometric*[tiab] OR valid*[tiab] | 'questionnaire'/exp OR (survey* OR questionnaire* OR instrument* OR assessment* OR measure* OR “screening tool*” OR psychometric* OR valid*):ti,ab,kw | MH "Questionnaires" OR TI(survey* OR questionnaire* OR instrument* OR assessment* OR measure* OR “screening tool*” OR psychometric* OR valid*) OR AB(survey* OR questionnaire* OR instrument* OR assessment* OR measure* OR “screening tool*” OR psychometric* OR valid*) OR KW(survey* OR questionnaire* OR instrument* OR assessment* OR measure* OR “screening tool*” OR psychometric* OR valid*) | DE "Questionnaires" OR TI(survey* OR questionnaire* OR instrument* OR assessment* OR measure* OR “screening tool*” OR psychometric* OR valid*) OR AB(survey* OR questionnaire* OR instrument* OR assessment* OR measure* OR “screening tool*” OR psychometric* OR valid*) OR KW(survey* OR questionnaire* OR instrument* OR assessment* OR measure* OR “screening tool*” OR psychometric* OR valid*) | DE "Questionnaires" OR TI(survey* OR questionnaire* OR instrument* OR assessment* OR measure* OR “screening tool*” OR psychometric* OR valid*) OR AB(survey* OR questionnaire* OR instrument* OR assessment* OR measure* OR “screening tool*” OR psychometric* OR valid*) OR KW(survey* OR questionnaire* OR instrument* OR assessment* OR measure* OR “screening tool*” OR psychometric* OR valid*) |
| #6 | Letter[pt] OR editorial[pt] OR comment[pt] | 'editorial'/it OR 'letter'/it OR 'note'/it |  |  |  |

| Search string combinations | Totaal | Pubmed | Embase | Cinahl | Psycinfo | Eric |
| --- | --- | --- | --- | --- | --- | --- |
| #1 OR #2 |  | 12,770 | [15,824](https://www-embase-com.vu-nl.idm.oclc.org/) | 8,877 | 6,125 | 2,111 |
| #3 OR #4 |  | 1,883,496 | 2,432,437 | 752,685 | 1,128,197 | 765,180 |
| #5 |  | 5,720,211 | 7,105,648 | 1,593,687 | 1,505,538 | 483,435 |
| (#1 OR #2) AND (#3 OR #4) AND #5 |  | 2,045 | 2,440 | 1,102 | 914 | 376 |
| **(#1 OR #2) AND (#3 OR #4) AND #5 NOT #6** | **6,857** | **2,036** | **2,429** | **1,102** | **914** | **376** |
|  |  |  |  |  |  |  |
| Original search outcome | 6,857 | 2,036 | 2,429 | 1,102 | 914 | 376 |
| Outcome after excluding PMIDs | 4,758 |  | 1,111 | 698 | 537 | 376 |
| After removing duplicates in Endnote | 3,955 | 2,035 | 1,022 | 267 | 386 | 245 |

## Search history updated search

For the updated search, we have used the original search elements #1 construct, #2 population and #3 document type and combined this in #4. We have added the exclusion of COVID (#5) in combination with excluded document types from the original search, as this is a disease and health care related context, with specific disease prevention (not fitting with health promotion in general), moreover it was a worldwide crisis which is not representative for everyday life in which children are brought up.

### History PubMed August 8, 2022

| **Search** | **PubMed Query – August 8, 2022** | **Items found** |
| --- | --- | --- |
| **#5** | **#4 NOT (covid[ti] OR Letter[pt] OR editorial[pt] OR comment[pt])** | **2,864** |
| #4 | #1 AND #2 AND #3 | 2,943 |
| #3 | "Surveys and Questionnaires"[Mesh] OR survey*[tiab] OR questionnaire*[tiab] OR instrument*[tiab] OR assessment*[tiab] OR measure*[tiab] OR “screening tool*”[tiab] OR psychometric*[tiab] OR valid*[tiab] | 6,628,972 |
| #2 | child*[tiab] OR adolescen*[tiab] OR schoolchild*[tiab] OR teenage*[tiab] OR teen[tiab] OR teens[tiab] OR youth[tiab] OR youths[tiab] OR “pre-teen*”[tiab] OR boy[tiab] OR boys[tiab] OR girl[tiab] OR girls[tiab] OR “young people”[tiab] OR school*[tiab] OR “afterschool”[tiab] OR “junior high”[tiab] OR “grade 4”[tiab] OR “grade 5”[tiab] OR “grade 6”[tiab] OR “grade 7“[tiab] OR “grade 8”[tiab] OR “grades 4”[tiab] OR “grades 5”[tiab] OR “grades 6”[tiab] OR “grades 7“[tiab] OR “grades 8”[tiab] OR “4th grade*“[tiab] OR “fourth grade*“[tiab] OR “5th grade*“[tiab] OR “fifth grade*“[tiab] OR “6th grade*“[tiab] OR “sixth grade*“[tiab] OR “7th grade*“[tiab] OR “seventh grade*“[tiab] OR “8th grade*“[tiab] OR “eighth grade*“[tiab] | 2,125,041 |
| #1 | "Health Literacy"[Mesh] OR “health literac*”[tiab] OR “ehealth literac*”[tiab] OR “physical literac*”[tiab] OR “oral literac*”[tiab] OR “nutrition literac*”[tiab] OR “nutritional literac*”[tiab] OR “food literac*”[tiab] OR “media literac*”[tiab] OR ((alcohol[tiab] OR drinking[tiab] OR drug*[tiab] OR cigarette*[tiab] OR smoking[tiab] OR tobacco[tiab] OR sexual*[tiab] OR sex[tiab] OR hiv[tiab] OR sun[tiab] OR sunscreen*[tiab] OR uv[tiab] OR “skin protect*”[tiab] OR fitness[tiab] OR “screen time”[tiab] OR “screen use”[tiab] OR hygien*[tiab]) AND literac*[tiab]) | 17,965 |

### History Embase.com August 8, 2022

| **Search** | **Embase.com Query – August 8, 2022** | **Items found** |
| --- | --- | --- |
| **#5** | **#4 NOT (covid:ti OR 'editorial'/it OR 'letter'/it OR 'note'/it)** | **3,445** |
| #4 | #1 AND #2 AND #3 | 3,516 |
| #3 | 'questionnaire'/exp OR (survey* OR questionnaire* OR instrument* OR assessment* OR measure* OR “screening tool*” OR psychometric* OR valid*):ti,ab,kw | 8,280,943 |
| #2 | (child* OR adolescen* OR schoolchild* OR teenage* OR teen OR teens OR youth OR youths OR “pre-teen*” OR boy OR boys OR girl OR girls OR “young people” OR school* OR afterschool OR “junior high”):ti,ab,kw OR (grade* NEAR/1 (4 OR 5 OR 6 OR 7 OR 8 OR 4th OR 5th OR 6th OR 7th OR 8th OR fourth OR fifth OR sixth OR seventh OR eighth)):ti,ab,kw | 2,768,017 |
| #1 | 'health literacy'/exp OR ((health OR ehealth OR physical OR oral OR nutrition* OR food OR media) NEXT/2 literac*):ti,ab,kw OR ((alcohol OR drinking OR drug* OR cigarette* OR smoking OR tobacco OR sexual* OR sex OR hiv OR sun OR sunscreen* OR uv OR “skin protect*” OR hiv OR fitness OR “screen time” OR “screen use” OR hygiene) NEAR/6 literac*):ti,ab,kw | 22,770 |

### History CINAHL (Ebsco) August 8, 2022

| **Search** | **CINAHL (Ebsco) Query – August 8, 2022** | **Items found** |
| --- | --- | --- |
| **S5** | **S4 NOT TI (covid)** | **1,376** |
| S4 | S1 AND S2 AND S3 | 1,393 |
| S3 | MH "Questionnaires" OR TI(survey* OR questionnaire* OR instrument* OR assessment* OR measure* OR “screening tool*” OR psychometric* OR valid*) OR AB(survey* OR questionnaire* OR instrument* OR assessment* OR measure* OR “screening tool*” OR psychometric* OR valid*) OR KW(survey* OR questionnaire* OR instrument* OR assessment* OR measure* OR “screening tool*” OR psychometric* OR valid*) | 1,735,526 |
| S2 | TI(child* OR adolescen* OR schoolchild* OR teenage* OR teen OR teens OR youth OR youths OR “pre-teen*” OR boy OR boys OR girl OR girls OR “young people” OR “school*” OR afterschool OR “junior high”) OR AB(child* OR adolescen* OR schoolchild* OR teenage* OR teen OR teens OR youth OR youths OR “pre-teen*” OR boy OR boys OR girl OR girls OR “young people” OR “school*” OR afterschool OR “junior high”) OR KW(child* OR adolescen* OR schoolchild* OR teenage* OR teen OR teens OR youth OR youths OR “pre-teen*” OR boy OR boys OR girl OR girls OR “young people” OR “school*” OR afterschool OR “junior high”) OR TI(grade* W1 (4 OR 5 OR 6 OR 7 OR 8 OR 4th OR 5th OR 6th OR 7th OR 8th OR fourth OR fifth OR sixth OR seventh OR eighth)) OR AB(grade* W1 (4 OR 5 OR 6 OR 7 OR 8 OR 4th OR 5th OR 6th OR 7th OR 8th OR fourth OR fifth OR sixth OR seventh OR eighth)) OR KW(grade* W1 (4 OR 5 OR 6 OR 7 OR 8 OR 4th OR 5th OR 6th OR 7th OR 8th OR fourth OR fifth OR sixth OR seventh OR eighth)) | 809,089 |
| S1 | MH "Health Literacy" OR TI((health OR ehealth OR physical OR oral OR nutrition* OR food OR media) N**1** literac*) OR AB((health OR ehealth OR physical OR oral OR nutrition* OR food OR media) N**1** literac*) OR KW((health OR ehealth OR physical OR oral OR nutrition* OR food OR media) N**1** literac*) OR TI((alcohol OR drinking OR drug* OR cigarette* OR smoking OR tobacco OR sexual* OR sex OR hiv OR sun OR sunscreen* OR uv OR “skin protect*” OR hiv OR fitness OR “screen time” OR “screen use” OR hygiene) W5 literac*) OR AB ((alcohol OR drinking OR drug* OR cigarette* OR smoking OR tobacco OR sexual* OR sex OR hiv OR sun OR sunscreen* OR uv OR “skin protect*” OR hiv OR fitness OR “screen time” OR “screen use” OR hygiene) W5 literac*) OR KW((alcohol OR drinking OR drug* OR cigarette* OR smoking OR tobacco OR sexual* OR sex OR hiv OR sun OR sunscreen* OR uv OR “skin protect*” OR hiv OR fitness OR “screen time” OR “screen use” OR hygiene) W5 literac*) | 11,174 |

### History APA PsycInfo (Ebsco) August 8, 2022

| **Search** | **APA PsycInfo (Ebsco) Query – August 8, 2022** | **Items found** |
| --- | --- | --- |
| **S5** | **S4 NOT TI (covid)** | **1,180** |
| S4 | S1 AND S2 AND S3 | 1,187 |
| S3 | DE "Questionnaires" OR TI(survey* OR questionnaire* OR instrument* OR assessment* OR measure* OR “screening tool*” OR psychometric* OR valid*) OR AB(survey* OR questionnaire* OR instrument* OR assessment* OR measure* OR “screening tool*” OR psychometric* OR valid*) OR KW(survey* OR questionnaire* OR instrument* OR assessment* OR measure* OR “screening tool*” OR psychometric* OR valid*) | 1,673,161 |
| S2 | TI(child* OR adolescen* OR schoolchild* OR teenage* OR teen OR teens OR youth OR youths OR “pre-teen*” OR boy OR boys OR girl OR girls OR “young people” OR “school*” OR afterschool OR “junior high”) OR AB(child* OR adolescen* OR schoolchild* OR teenage* OR teen OR teens OR youth OR youths OR “pre-teen*” OR boy OR boys OR girl OR girls OR “young people” OR “school*” OR afterschool OR “junior high”) OR KW(child* OR adolescen* OR schoolchild* OR teenage* OR teen OR teens OR youth OR youths OR “pre-teen*” OR boy OR boys OR girl OR girls OR “young people” OR “school*” OR afterschool OR “junior high”) OR TI(grade* W1 (4 OR 5 OR 6 OR 7 OR 8 OR 4th OR 5th OR 6th OR 7th OR 8th OR fourth OR fifth OR sixth OR seventh OR eighth)) OR AB(grade* W1 (4 OR 5 OR 6 OR 7 OR 8 OR 4th OR 5th OR 6th OR 7th OR 8th OR fourth OR fifth OR sixth OR seventh OR eighth)) OR KW(grade* W1 (4 OR 5 OR 6 OR 7 OR 8 OR 4th OR 5th OR 6th OR 7th OR 8th OR fourth OR fifth OR sixth OR seventh OR eighth)) | 1,231,077 |
| S1 | DE "Health Literacy" OR TI((health OR ehealth OR physical OR oral OR nutrition* OR food OR media) N1 literac*) OR AB((health OR ehealth OR physical OR oral OR nutrition* OR food OR media) N1 literac*) OR KW((health OR ehealth OR physical OR oral OR nutrition* OR food OR media) N1 literac*) OR TI((alcohol OR drinking OR drug* OR cigarette* OR smoking OR tobacco OR sexual* OR sex OR hiv OR sun OR sunscreen* OR uv OR “skin protect*” OR hiv OR fitness OR “screen time” OR “screen use” OR hygiene) W5 literac*) OR AB ((alcohol OR drinking OR drug* OR cigarette* OR smoking OR tobacco OR sexual* OR sex OR hiv OR sun OR sunscreen* OR uv OR “skin protect*” OR hiv OR fitness OR “screen time” OR “screen use” OR hygiene) W5 literac*) OR KW((alcohol OR drinking OR drug* OR cigarette* OR smoking OR tobacco OR sexual* OR sex OR hiv OR sun OR sunscreen* OR uv OR “skin protect*” OR hiv OR fitness OR “screen time” OR “screen use” OR hygiene) W5 literac*) | 7,824 |

### History ERIC (Ebsco) August 8, 2022

| **Search** | **ERIC (Ebsco) Query – August 8, 2022** | **Items found** |
| --- | --- | --- |
| **S5** | **S4 NOT TI (covid)** | **437** |
| S4 | S1 AND S2 AND S3 | 439 |
| 4S3 | DE "Questionnaires" OR TI(survey* OR questionnaire* OR instrument* OR assessment* OR measure* OR “screening tool*” OR psychometric* OR valid*) OR AB(survey* OR questionnaire* OR instrument* OR assessment* OR measure* OR “screening tool*” OR psychometric* OR valid*) OR KW(survey* OR questionnaire* OR instrument* OR assessment* OR measure* OR “screening tool*” OR psychometric* OR valid*) | 511,629 |
| S2 | TI(child* OR adolescen* OR schoolchild* OR teenage* OR teen OR teens OR youth OR youths OR “pre-teen*” OR boy OR boys OR girl OR girls OR “young people” OR “school*” OR afterschool OR “junior high”) OR AB(child* OR adolescen* OR schoolchild* OR teenage* OR teen OR teens OR youth OR youths OR “pre-teen*” OR boy OR boys OR girl OR girls OR “young people” OR “school*” Or afterschool OR “junior high”) OR KW(child* OR adolescen* OR schoolchild* OR teenage* OR teen OR teens OR youth OR youths OR “pre-teen*” OR boy OR boys OR girl OR girls OR “young people” OR “school*” OR afterschool OR “junior high”) OR TI(grade* W1 (4 OR 5 OR 6 OR 7 OR 8 OR 4th OR 5th OR 6th OR 7th OR 8th OR fourth OR fifth OR sixth OR seventh OR eighth)) OR AB(grade* W1 (4 OR 5 OR 6 OR 7 OR 8 OR 4th OR 5th OR 6th OR 7th OR 8th OR fourth OR fifth OR sixth OR seventh OR eighth)) OR KW(grade* W1 (4 OR 5 OR 6 OR 7 OR 8 OR 4th OR 5th OR 6th OR 7th OR 8th OR fourth OR fifth OR sixth OR seventh OR eighth)) | 800,840 |
| S1 | TI((health OR ehealth OR physical OR oral OR nutrition* OR food OR media) N1 literac*) OR AB((health OR ehealth OR physical OR oral OR nutrition* OR food OR media) N1 literac*) OR KW((health OR ehealth OR physical OR oral OR nutrition* OR food OR media) N1 literac*) OR TI((alcohol OR drinking OR drug* OR cigarette* OR smoking OR tobacco OR sexual* OR sex OR hiv OR sun OR sunscreen* OR uv OR “skin protect*” OR hiv OR fitness OR “screen time” OR “screen use” OR hygiene) W5 literac*) OR AB ((alcohol OR drinking OR drug* OR cigarette* OR smoking OR tobacco OR sexual* OR sex OR hiv OR sun OR sunscreen* OR uv OR “skin protect*” OR hiv OR fitness OR “screen time” OR “screen use” OR hygiene) W5 literac*) OR KW((alcohol OR drinking OR drug* OR cigarette* OR smoking OR tobacco OR sexual* OR sex OR hiv OR sun OR sunscreen* OR uv OR “skin protect*” OR hiv OR fitness OR “screen time” OR “screen use” OR hygiene) W5 literac*) | 2,434 |

Explanation of selection through ASReview:

ASReview is a selection tool for reviews based on active learning. It prioritizes found publications based on inclusion and exclusion criteria which are entered by the researcher in this case all included studies from the original search and related excluded studies from the original search. Based on large language models the tool prioritizes the publications in which similar descriptions are found as included publications. We have entered the 20 publications from the original search as included publications and we entered 20 publications which we excluded based on the various reasons for exclusion mentioned in the flowchart. The tool prioritized the publications based on our initial search and selection. The most relevant publications were presented first and is adapted by each decision the reviewer makes, to present the potentially most relevant articles first. Articles were screened for relevance based on the same criteria as the original search, following the stopping rule for screening; after screening 150 papers, stop when finding 30 irrelevant papers (Van den Brand, S.A.G.E., & van de Schoot, R., 2021). To describe the complete process and our stopping point: we uploaded 1,456 new records in total, screened 217 titles and abstracts and stopped at 51 irrelevant records after the last relevant. A total of 15 were identified as relevant in the updated search and included for fulltext review. In the figure the selection procedure is presented with relevant (included) records on the y-axis and the number of reviewed records of the x-axis

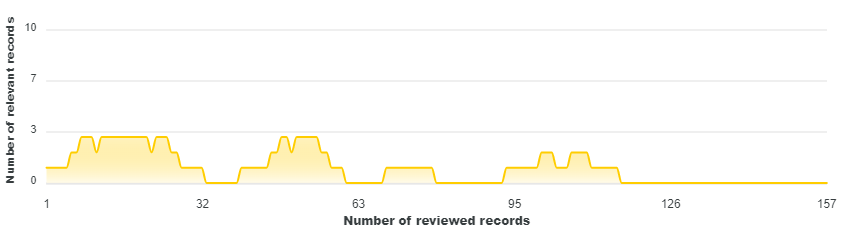

Supplement: Supplementary file 4 — Supplementary Material 4: Search strategies [file 12889_2024_17955_MOESM4_ESM.docx]
